# Effects of high-intensity interval training and moderate-intensity continuous training on body composition and glucose and lipid metabolism in college students: a systematic review and meta-analysis

**DOI:** 10.3389/fendo.2026.1894767

**Published:** 2026-07-15

**Authors:** Haoyu Sun, Vitaliy V. Khramov, Jiandong Li, Mingyi Wang

**Affiliations:** 1 School of Sports and Technology, Guangzhou College of Applied Science and Technology, Zhaoqing, China; 2 Faculty of Physical Education and Sport, Yanka Kupala State University of Grodno, Grodno, Belarus; 3 Department of Sports Management, Guangzhou Cadre and Talent Health Management Center, Guangzhou, China

**Keywords:** body composition, college students, glucose metabolism, high-intensity interval training, lipid metabolism, moderate-intensity continuous training

## Abstract

**Background:**

This study systematically evaluated high-intensity interval training (HIIT) versus moderate-intensity continuous training (MICT) for body composition and glucose and lipid metabolism markers in college students.

**Methods:**

This PRISMA 2020–based systematic review searched PubMed, Cochrane Library, Web of Science, Embase, China National Knowledge Infrastructure (CNKI), and VIP Database (VIP) from inception to March 10, 2026. Restricted maximum likelihood (REML) random-effects models pooled mean differences (MDs) with 95% confidence intervals (CIs), and heterogeneity was assessed using Cochran’s Q and I^2^. Subgroup, meta-regression, sensitivity, publication bias, RoB 2.0, and GRADE analyses were conducted.

**Results:**

A total of 20 randomized controlled trials (RCTs) involving 745 college students were included, with 377 in HIIT and 368 in MICT. Compared with MICT, HIIT significantly reduced body weight (MD = -1.23 kg, 95% CI -1.94 to -0.52, P = 0.002), body fat percentage (MD = -1.21%, 95% CI -2.12 to -0.30, P = 0.013), waist-to-hip ratio (MD = -0.01, 95% CI -0.02 to -0.00, P = 0.014), waist circumference (MD = -1.33 cm, 95% CI -2.40 to -0.26, P = 0.023), and fat mass (MD = -0.99 kg, 95% CI -1.66 to -0.33, P = 0.010). No significant differences were found for body mass index (BMI), hip circumference, muscle mass, total cholesterol (TC), triglycerides (TG), high-density lipoprotein cholesterol (HDL-C), low-density lipoprotein cholesterol (LDL-C), fasting blood glucose (FBG), or fasting insulin (FINS). Subgroup analyses suggested sex might influence body weight and BMI effects, but most analyses showed no clear moderation. Meta-regression detected no significant moderation by duration or weekly HIIT volume. Evidence for body composition outcomes was mostly moderate, low, or very low; evidence for glucose and lipid metabolism outcomes was very low.

**Conclusion:**

Compared with MICT, HIIT may provide greater benefits for selected body composition measures in college students, particularly body weight, body fat percentage, waist circumference, waist-to-hip ratio, and fat mass. However, HIIT did not consistently improve routine glucose and lipid metabolism markers. Given the limited evidence quality, small number of studies, and uncertain moderator findings from subgroup and meta-regression analyses, larger randomized controlled trials with standardized protocols are needed.

**Systematic Review Registration:**

https://www.crd.york.ac.uk/PROSPERO/, identifier CRD420261396573.

## Introduction

Physical inactivity and the obesity epidemic together create major challenges for the global prevention and control of chronic diseases. The World Health Organization considers regular exercise an essential public health strategy for improving health and reducing the risk of chronic diseases. It recommends that adults engage in at least 150 minutes of moderate-intensity aerobic activity or 75 minutes of vigorous-intensity aerobic activity each week (1). However, physical inactivity remains a major global problem, with a prevalence rate of 31.3% in 2022, affecting about 1.8 billion people (2). Meanwhile, the prevalence of overweight and obesity continued to grow. In the same year, about 2.5 billion adults worldwide were classified as overweight, with around 890 million categorized as obese (3, 4). Given the close link between excessive fat buildup and problems in glucose and lipid metabolism, as well as cardiovascular disease and other non-communicable illnesses, it is vital to identify effective, quick, and scalable exercise interventions. These interventions are crucial for enhancing body composition and metabolic health across the population.

College students experience a vital transition from adolescence to adulthood, during which changes in their learning environment, daily routines, and levels of independence can greatly influence their physical activity, sedentary habits, and weight status. Prior research shows that physical inactivity and prolonged periods of sedentary behavior are common among college students (5, 6). Weight gain after college enrollment indicates that this period is a key opportunity for applying weight management strategies and lifestyle interventions (7, 8). Additionally, the levels of physical activity among college students are closely connected to their weight status and overall health. This further highlights the need for exercise intervention studies targeting this population (9). Despite growing evidence on health behavior interventions among college students, there is no consensus on which training method is most effective for improving body composition and metabolic indicators (10).

MICT is a commonly used type of aerobic exercise in traditional workout plans. It features a steady intensity, good sustainability, and is easy to implement (11). In contrast, HIIT alternates high-intensity exercise with intermittent recovery, thereby providing significant physiological stimuli in a shorter time and inducing cardiorespiratory and metabolic adaptations (12, 13). However, HIIT protocols were heavily influenced by factors such as training intensity, interval duration, recovery mode, training frequency, and intervention duration. These variables may have played a role in the differences seen in results across various studies (14).

Existing evidence generally supports HIIT’s potential to improve cardiorespiratory fitness and certain cardiometabolic outcomes. Systematic reviews focusing on populations with lifestyle-related cardiometabolic risks suggest that HIIT can be an effective training method for enhancing cardiometabolic health (15, 16). Regarding glucose metabolism, previous meta-analyses indicate that HIIT may help improve glycemic control and insulin sensitivity (17). However, when the research shifts to body composition indicators such as body weight, BMI, body fat percentage, waist circumference, and fat mass, the advantages of HIIT over MICT become less consistent. Some evidence supports HIIT’s potential to reduce abdominal and visceral fat (18), and low-volume HIIT also has practical value because of its shorter training duration (19). Systematic reviews directly comparing HIIT and MICT suggest that differences in their effects on body composition may be small. The advantages of HIIT may not always appear as larger absolute effects but could instead be in time efficiency or specific outcomes (20–22). Consequently, uncertainty remains regarding the relative effects of HIIT and MICT on body composition and glucose and lipid metabolism. Clarifying these effects is particularly important for college students, to whom existing evidence may not fully apply.

Although college students are often grouped with general adult populations, their baseline metabolic status may range from apparently healthy to overweight, obesity, or normal-weight obesity. By contrast, participants in obesity or diabetes cohorts commonly have established metabolic abnormalities and greater scope for improvement (15, 16). Consequently, findings from clinical populations may not be directly generalizable to college students. Previous HIIT-versus-MICT meta-analyses have mainly pooled general adults or focused on populations with overweight, obesity, or cardiometabolic disease (20–22), but the comparative effects of these interventions on body composition and glucose and lipid metabolism specifically in college students remain unclear. Studies in this population also vary in weight status, sex, intervention duration, training frequency, and exercise protocol, which may further modify these outcomes. Beyond physiological responses, campus implementation may also depend on exercise enjoyment, acceptability, and long-term adherence (23).

Accordingly, this systematic review and meta-analysis aimed to address this population-specific evidence gap by synthesizing direct comparisons of HIIT and MICT in college students for body composition outcomes (body weight, BMI, body fat percentage, waist-to-hip ratio, waist circumference, hip circumference, fat mass, and muscle mass) and glucose and lipid metabolism markers (blood lipids, blood glucose, and insulin), while exploring potential effect modification by weight status, sex, intervention duration, and training frequency. This population-specific synthesis may help inform campus health promotion and student weight-management strategies while identifying evidence gaps relevant to the future refinement of HIIT exercise prescriptions.

## Materials and methods

### Study design

This meta-analysis was conducted following the PRISMA 2020 statement (24). This study was registered with the International Prospective Register of Systematic Reviews (PROSPERO; registration number: CRD420261396573).

### Search strategy

The search strategy was developed following the recommendations of the Cochrane Handbook for literature searches in systematic reviews and the reporting requirements of PRISMA-S (25, 26). Six databases, including Web of Science, Cochrane Library, CNKI, VIP, Embase, and PubMed, were systematically searched for relevant studies published from the inception of each database to March 10, 2026. The search strategy combined controlled vocabulary and free-text terms, and the search fields and expressions were adjusted according to each database’s retrieval rules. The main search terms included “high-intensity interval training,” “interval training,” “moderate-intensity continuous training,” “continuous training,” “college students,” and “students.” Additionally, the reference lists of the included studies and relevant reviews were manually screened. The detailed search strategies for each database are provided in **Supplementary File S1**.

### Study inclusion criteria

The inclusion and exclusion criteria were established based on the PICOS framework. The research question and eligibility criteria were predefined for participants, interventions, comparators, outcomes, and study design. Studies were included only if they met all of the following criteria: the study design was a RCT; the participants were college students aged 18–26 years, regardless of sex or weight status; the intervention was HIIT and the comparator was MICT; at least one predefined continuous outcome related to body composition or glucose and lipid metabolism was reported; and the sample size, mean, and standard deviation for both groups were available or could be calculated. The predefined outcomes included body weight, BMI, body fat percentage, waist-to-hip ratio, waist circumference, hip circumference, fat mass, muscle mass, TC, TG, HDL-C, LDL-C, FBG, and FINS. Studies were excluded if they were duplicate publications, review articles, conference abstracts without sufficient data, or lacked valid data for effect-size calculation.

### Data extraction

The retrieved records were imported into EndNote 21 for Mac (Clarivate) for reference management and duplicate removal. Two researchers independently screened titles, abstracts, and full texts according to predefined inclusion and exclusion criteria, resolving any disagreements through a third researcher. For studies with missing or incompletely reported data, the original authors were contacted initially. When no response was received, data were calculated based on the available information in the articles. The extracted information included the first author, publication year, study design, sample size, participants’ age and sex, intervention duration, HIIT and MICT protocols, outcome measures, and the pre- and post-intervention means and standard deviations for each outcome in both groups. To facilitate subgroup and meta-regression analyses, weight status, sex, intervention duration, training frequency, exercise duration per session, and weekly HIIT training volume were systematically extracted and coded.

### Risk of bias (quality) assessment

Two researchers independently assessed the risk of bias and the quality of evidence in the included studies. The Cochrane RoB 2.0 tool was used to evaluate the risk of bias in RCTs (27). Judgments were made across five domains: the randomization process, deviations from intended interventions, missing outcome data, measurement of outcomes, and selection of reported results, resulting in an overall risk-of-bias assessment. The GRADE approach was used to evaluate the quality of evidence for each outcome. The assessment domains included risk of bias, inconsistency, indirectness, imprecision, and publication bias, with the certainty of evidence classified as high, moderate, low, or very low (28). A third researcher resolved disagreements.

### Data synthesis

All statistical analyses were conducted in R software (version 4.5.0; R Foundation for Statistical Computing) using the meta and metafor packages (29). Because the measurement units of each outcome were consistent across studies, continuous variables were reported as MDs with 95% CIs (30). A random-effects model was used for pooling, with the between-study variance τ^2^ estimated by REML. The Hartung-Knapp method was applied to adjust the confidence intervals of the pooled effects (31, 32). All quantitative syntheses were performed using pre- to post-intervention change values. For multiple intervention arms sharing the same control group, the sample sizes, means, and standard deviations were combined following the formulas recommended in the Cochrane Handbook before including them in the analysis (30). When change values and their standard deviations were not reported, they were estimated using the difference between pre- and post-intervention means and the method recommended in the Cochrane Handbook, respectively, with a correlation coefficient of 0.5. To assess whether the assumption about the correlation coefficient affected the stability of the pooled effects, sensitivity analyses were additionally conducted by setting r to 0.25 and 0.75 (30). Between-study heterogeneity was evaluated using Cochran’s Q test and the I^2^ statistic and interpreted according to the commonly used thresholds proposed by Higgins et al., where I^2^ values of 25%, 50%, and 75% indicate low, moderate, and high heterogeneity, respectively (33). When significant heterogeneity was observed, leave-one-out sensitivity analysis was performed to assess the robustness of the results. Publication bias was evaluated using funnel plots along with Egger’s test. Formal testing was carried out when the number of included studies was ≥10, while results were interpreted only exploratorily when the number of studies was <10 (32, 34). Except for heterogeneity tests, a two-sided P value of less than 0.05 was considered statistically significant.

### Subgroup analysis

To determine whether participant characteristics and intervention protocols affected the effects of HIIT and MICT, we performed subgroup analyses focusing on body composition indicators and glucose- and lipid-metabolism-related markers. Body composition indicators were categorized based on weight status (normal weight, overweight, and obesity), sex (male and female), intervention duration (≤8 weeks and >8 weeks), and training frequency (≤3 sessions/week and >3 sessions/week). Weight status was mainly determined by participant characteristics, inclusion criteria, and baseline BMI reported in the original studies. Because of the limited number of studies on glucose and lipid metabolism indicators, these outcomes were mostly stratified by weight status and sex. Differences between subgroups were evaluated using interaction tests, and the interaction P value was reported. In this study, each subgroup needed to include at least two studies; otherwise, the pooled effect estimate was not calculated. Results were considered exploratory when fewer than 10 studies were available for subgroup difference testing or when the distribution of subgroup levels was significantly unbalanced.

### Meta-regression analyses

To further investigate potential sources of between-study heterogeneity and the moderating effects of intervention characteristics, we conducted univariable random-effects meta-regression analyses for outcomes reported in ≥10 studies. Intervention duration (weeks) and weekly HIIT training volume (per 10 min/week) were entered separately as continuous moderators. Weekly HIIT training volume was calculated by multiplying prescribed session duration, including work and recovery intervals but excluding warm-up and cool-down, by sessions per week.

## Results

### Literature search results

**Figure 1:**
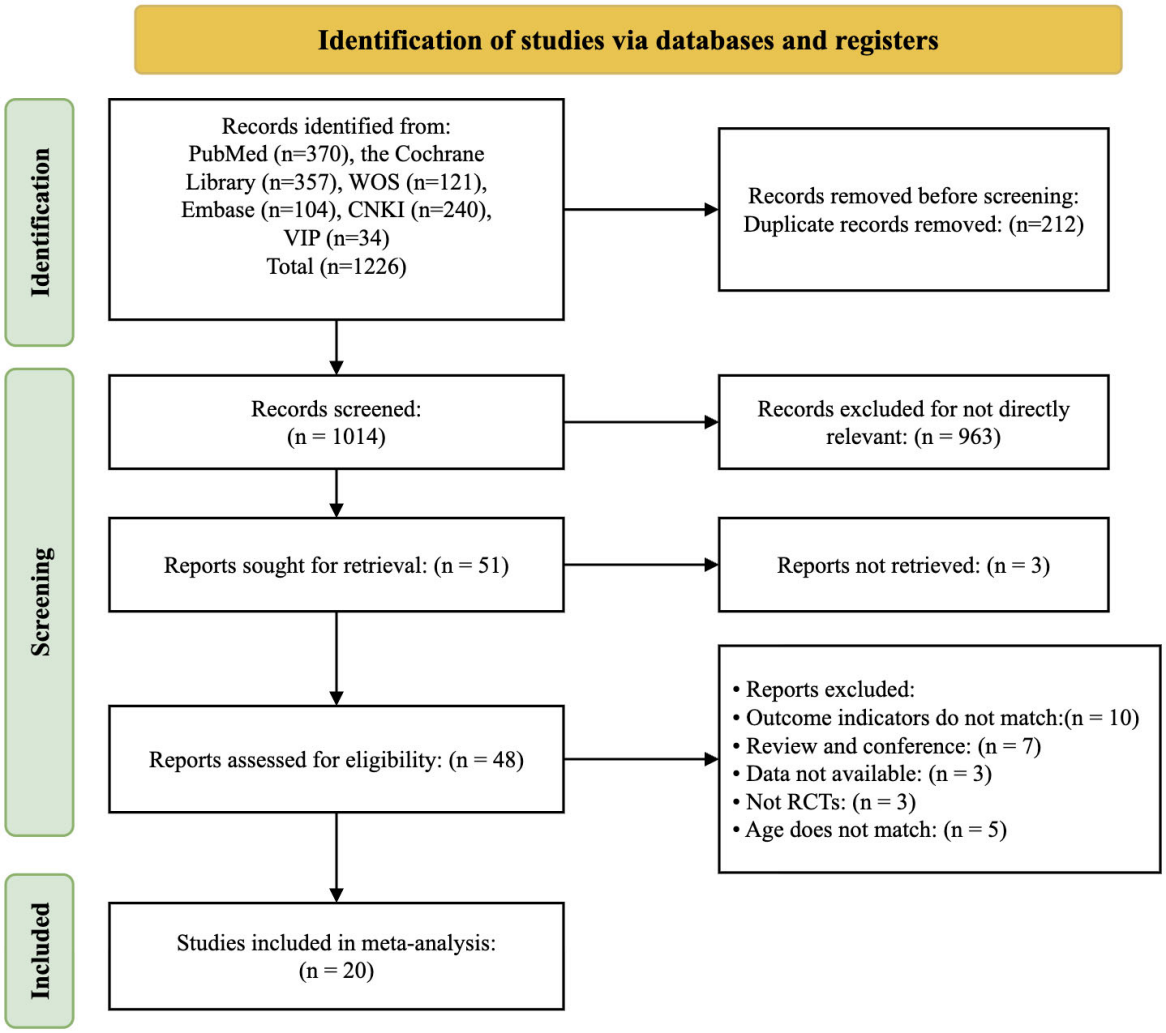
PRISMA flow diagram of study selection.

According to the predefined search strategy, a total of 1,226 relevant records were retrieved from PubMed, Cochrane Library, Web of Science, Embase, CNKI, and the VIP Database. After removing 212 duplicate records, 1,014 records were screened based on their titles and abstracts, leading to the exclusion of 963 records that were not directly relevant to the research topic. Subsequently, 51 full-text articles were sought; however, 3 were excluded due to the unavailability of the full texts. After assessing the eligibility of the remaining 48 full-text articles, 28 were excluded for the following reasons: inconsistent outcome indicators (n = 10), review or conference articles (n = 7), unavailable data (n = 3), non-RCT (n = 3), and ineligible participants (n = 5). In the end, 20 articles were included in the meta-analysis (35–54). The detailed process for selecting studies is shown in **Figure 1**.

### Basic characteristics of the included literature

A total of 20 studies published between 2016 and 2025 were included, involving 745 college students: 377 in the HIIT group and 368 in the MICT group. The participants were mostly aged 18 to 23 years, although four studies did not specify age details. The sample included male, female, and mixed-sex participants. Based on baseline BMI, participants were distributed across weight categories, including normal weight, overweight, obesity, and normal-weight obesity. The duration of interventions ranged from 4 to 16 weeks, with training frequency varying from 2 to 6 sessions per week. HIIT protocols mainly included running or treadmill exercises, cycle ergometer workouts, swimming, body-weight exercise circuits, combat aerobics, and Tabata training. In contrast, MICT protocols mainly involved continuous running, cycling, swimming, and continuous exercise circuits. The outcomes assessed included body composition measures such as body weight, BMI, body fat percentage, waist and hip circumferences, waist-to-hip ratio, fat mass, and muscle mass, as well as indicators related to glucose and lipid metabolism, including TC, TG, HDL-C, LDL-C, FBG, and FINS. The key features of the included studies are shown in **Table 1**.

**Table 1:** Characteristics of the included studies.

Study	Sample size	Age	Sex/BMI	Duration/frequency	HIIT protocol	MICT protocol	Outcomes
Ru X (52).	13/13	18-22	Male; BMI: 28.72/29.42	8 weeks; 5 sessions/week	Body-weight exercise circuit, 4 x 30 s; 85%-95% HRmax; 10–12 s between exercises and 35 s between sets	Running, 60%-70% HRmax, 30–35 min	①②③④⑤⑥
Zhou L.J (47).	15/15	NR	Male; BMI: 27.49/26.87	12 weeks; 6 sessions/week	Combat aerobics, 4 x 4 min, 16 min in total; 85%-95% HRpeak; 2-min between-set rest	Running, 60%-75% HRpeak, 32 min	①②③⑥⑦⑧
Shen Q.P (43).	40/40	19 ± 0.52	Female; BMI: 20.11/20.58	10 weeks; 2 sessions/week	Exercise circuit plus 20-s sprint run; 85% VO2max; about 30 min/session	Exercise circuit plus 20-s sprint run; 65%-75% VO2max; continuous exercise >=30 min	②
Zhai Q (44).	36/36	NR	Male; BMI: 23.20/23.19	8 weeks; 3 sessions/week	8 x 400 m running, completed within 110 s; 110-s between-set rest	Continuous running, 60%-70% HRmax, 30 min	②
Yang R.Z (45).	12/12	20 ± 2	Male; BMI: 26.6/26.8	12 weeks; 4 sessions/week	Swimming, 8 x 50 m; >=85% HRmax; 2 min exercise/1 min rest, 30 min	Swimming, continuous 400 m; 60%-80% HRmax, 30 min	①②③⑦⑨⑩⑬⑭
Chen J.M (40).	12/12	20.57 ± 1.08	Female; BMI: 25.76/25.70	4 weeks; 3 sessions/week	Tabata body-weight circuit, 8 x 5; 70%-94% HRmax; 20 s work/10 s rest	Continuous running, 50%-69% HRmax, 40 min	①②③
Zhao J (38).	19/18	NR	Female; BMI: 31.11/31.17	12 weeks; 5 sessions/week	Cycle ergometer; 5 x 5 min at 90%-95% HRpeak, with 5 x 3 min recovery at 50%-60% HRpeak	Continuous cycling, 60%-70% HRpeak, 40 min	①②⑦⑧
Hu M (41).	15/15	21.2 ± 1.4	Female; BMI: 25.5/25.8	12 weeks; 3 sessions/week	Cycle ergometer, 90% VO2peak; 4 min work/3 min recovery	Continuous cycling, 60% VO2peak, 65 min	①②③⑦
Liu H.F (35).	20/20	20-23	Female; BMI: 28.76/28.82	12 weeks; 4 sessions/week	Cycle ergometer, 90% VO2max; 1 min high intensity/1 min interval, 15 repetitions	Continuous cycling, 50% VO2max, 30 min	①②③④⑤⑥⑨⑩⑪⑫⑬
Sun S (37).	14/14	21.2 ± 1.4	Female; BMI: 26.3/26.5	12 weeks; 3 sessions/week	Cycle ergometer, 90% VO2peak; 4 min work/3 min passive recovery, until 200–300 kJ	Continuous cycling, 60% VO2peak, until 200–300 kJ (about 52–69 min)	①②⑬⑭
Song X (48).	20/20	21.00-22.20	Mixed; BMI: 29.03-29.85	8 weeks; 3 sessions/week	Treadmill running, 4 x 4 min high-intensity running (85%-90% HRmax) plus 3 min low-intensity recovery (50%-60% HRmax), 28 min	Continuous treadmill running, 60%-70% HRmax, 35 min	①②③④⑤⑥
Shi W.X (49).	21/22	18-22	Male; BMI: 30.21/30.21	8 weeks; 3–4 sessions/week	Exercise circuit, 80%-95% HRmax; progressive intensity, 10–30 min	Aerobic walking/running, 60%-70% HRmax, 45 min	①②④⑩⑪⑫
Gao Y.M (36).	17/17	21.6 ± 1.4	Mixed; BMI: 27.13/27.84	12 weeks; 5 sessions/week	Treadmill, 4 min at 85% VO2max plus 2 min recovery at 50% VO2max, 5 sets	Treadmill, 60% VO2max, 40 min	①②③④⑤⑥⑨⑩⑪⑫
Zhu X (51).	20/9	19.5-21.0	Female; BMI: 27.1/27.4/25.1	12 weeks; 3–4 sessions/week	Cycle ergometer, two HIIT arms: 90% VO2max, 4 min/3 min rest x 5–7 repetitions; 120% VO2max, 1 min/1.5 min rest x 16–21 repetitions; both until 200 kJ	Cycle ergometer, 60% VO2max, until 200 kJ (about 51–61 min)	①②③⑦⑧
Lan C (46).	12/13	20.77-21.33	Mixed; BMI: 22.31/22.83	8 weeks; 3 sessions/week	Treadmill, 85%-95% HRmax; 4 min high intensity plus 3 min low intensity x 4, 28 min	Continuous running, 64%-76% HRmax, 45 min	③⑦⑧
Sun J (39).	12/13	21.71 ± 1.44	Male; BMI: 30.85/30.54	12 weeks; 5 sessions/week	Treadmill, 4 min at 85% VO2max plus 2 min recovery at 50% VO2max, 5 sets	Treadmill, 60% VO2max, 40 min	①②③④⑤⑥⑨⑩⑪⑫⑬⑭
Yang W.L (50).	16/16	20.4-21.8	Female; BMI: 28.52/27.79	12 weeks; 4 sessions/week	Treadmill, 3 min at 85% VO2max plus 2 min recovery at 50% VO2max, 6 sets, 30 min	Treadmill, 60% VO2max, 50 min	①②③④⑤⑥⑨⑩⑪⑫⑬⑭
Xiao T (42).	23/22	20.3-21.0	Female; BMI: 26.2/26.1	12 weeks; 3 sessions/week	Cycle ergometer, 90% VO2max; 4 min work/3 min interval, until 300 kJ (about 35.3 ± 4.1 min)	Cycle ergometer, 60% VO2max, until 300 kJ (about 69.2 ± 6.1 min)	①②③⑦
Bulqini A (53).	10/10	19-22	Sex not reported; BMI: 20.34/21.65	4 weeks; 3 sessions/week	15–17 s high intensity (100%-120% MAS) plus 15 s low intensity (70% MAS), 10 repetitions/set x 4 sets, 3-min between-set rest	Continuous exercise at 70% MAS, 40–60 min	②⑦
Guo X.F (54).	30/31	NR	Female; BMI: 22.91/22.85	16 weeks; 3 sessions/week	Treadmill, 85% HRVO2max; 3 min running plus 2 min interval x 5, 25 min	Continuous exercise at 60% HRVO2max, 50 min	②③

Sample size and BMI are reported as HIIT/MICT. ① body weight; ② body mass index; ③ body fat percentage; ④ waist circumference; ⑤ hip circumference; ⑥ waist-to-hip ratio; ⑦ fat mass; ⑧ muscle mass; ⑨ total cholesterol; ⑩ triglycerides; ⑪ high-density lipoprotein cholesterol; ⑫ low-density lipoprotein cholesterol; ⑬ fasting blood glucose; ⑭ fasting insulin. BMI, body mass index; HIIT, high-intensity interval training; MICT, moderate-intensity continuous training; HRmax, maximum heart rate; HRpeak, peak heart rate; VO2max, maximal oxygen uptake; VO2peak, peak oxygen uptake; MAS, maximal aerobic speed; HRVO2max, heart rate corresponding to maximal oxygen uptake.

### Risk of bias of included studies

**Figure 2:**
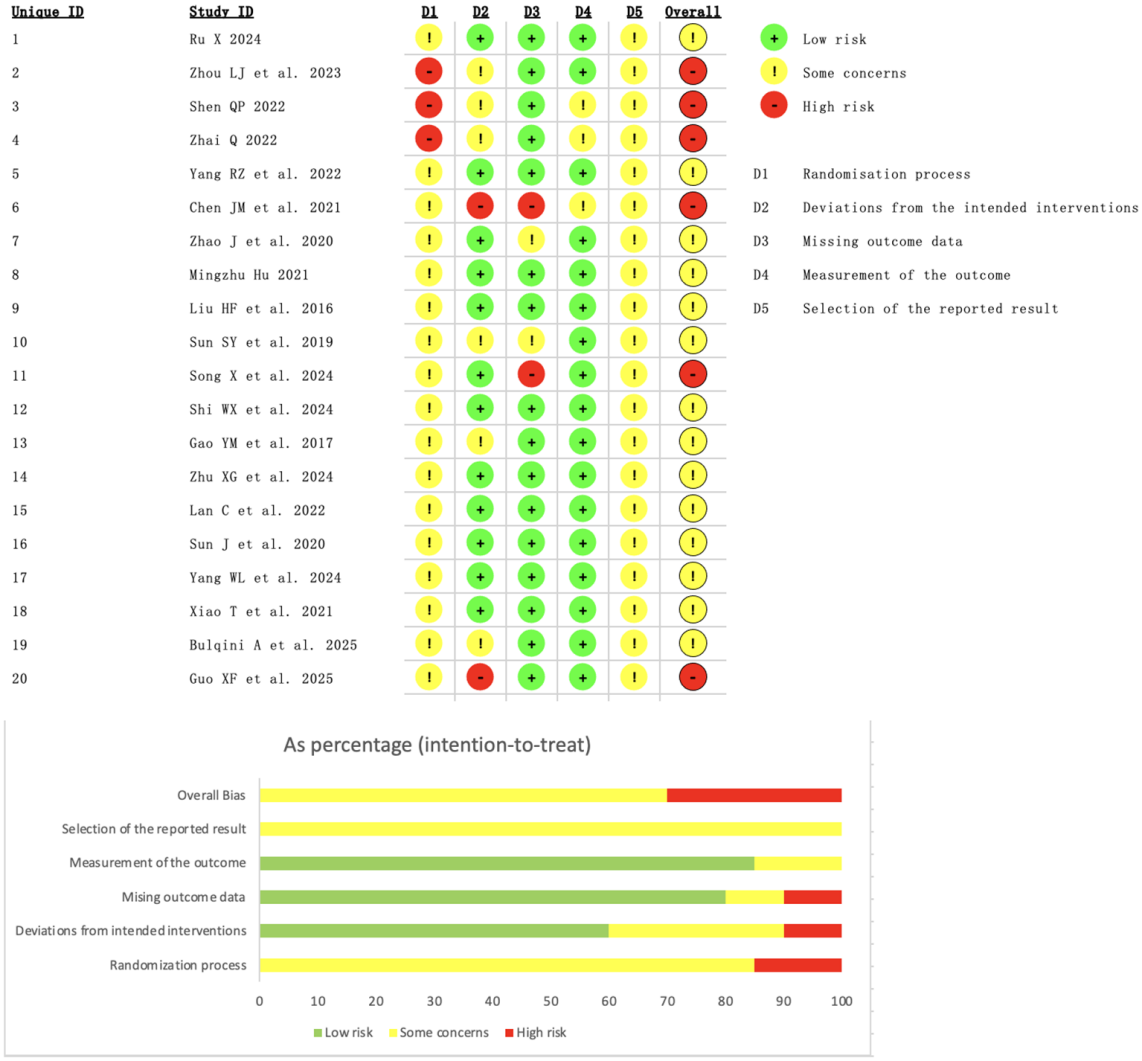
Risk of bias assessment of the included studies using the RoB 2 tool.

The risk of bias in the 20 included RCTs was evaluated using the RoB 2.0 tool (**Figure 2**). Of the studies, 18 were rated as having a low risk of missing outcome data, while 2 were classified as high risk. Regarding outcome measurement, 17 studies were considered to have a low risk, whereas 3 studies raised some concerns, suggesting that the included studies were generally reliable in this regard. Regarding deviations from intended interventions, 12 studies were rated as low risk, 6 as having some concerns, and 2 as high risk. Conversely, the randomization process identified 17 studies with concerns, and 3 were rated as high risk. All 20 studies were found to have some concerns regarding the selection of reported results. Overall, the included studies showed relatively strong performance in handling missing outcome data, outcome measurement, and certain aspects of intervention implementation; however, the main sources of bias were found in the randomization process and selective outcome reporting.

The GRADE approach was used to assess the quality of evidence for each outcome. The quality of evidence across the included outcomes ranged from moderate to very low. Specifically, evidence related to body composition outcomes was mainly rated as moderate, low, or very low. In contrast, all outcomes linked to glucose and lipid metabolism were classified as very low-quality evidence. The main reasons for downgrading included a high risk of bias, significant heterogeneity, and imprecision due to small sample sizes or 95% CIs crossing the line of no effect. For outcomes with fewer than 10 studies, publication bias was not used as a reason to downgrade the evidence; instead, funnel plots and Egger’s tests were used only as exploratory tools. The certainty of evidence for each outcome is specified in the relevant results sections (**Tables 2**, **3**), with the overall evidence profile included in **Supplementary File S2**.

**Table 2:** Meta-Analysis Results of the Effects of HIIT and MICT on Body Composition-Related Indicators.

Outcome	k	n	MD (95% CI)	p-value	I^2^ (%)	GRADE
Body weight (kg)	15	487	-1.23 (-1.94, -0.52)	0.002*	0	⊕⊕⊕⊖
Body mass index (kg/m^2^)	19	720	-0.31 (-0.62, 0.01)	0.055	29.6	⊕⊕⊖⊖
Body fat percentage (%)	14	465	-1.21 (-2.12, -0.30)	0.013*	77.8	⊕⊕⊖⊖
Waist-to-hip ratio	7	227	-0.01 (-0.02, -0.00)	0.014*	0	⊕⊕⊕⊖
Waist circumference (cm)	7	240	-1.33 (-2.40, -0.26)	0.023*	0	⊕⊕⊕⊖
Hip circumference (cm)	6	197	-0.24 (-1.08, 0.60)	0.491	0	⊕⊖⊖⊖
Fat mass (kg)	8	240	-0.99 (-1.66, -0.33)	0.010*	0	⊕⊕⊕⊖
Muscle mass (kg)	4	121	1.02 (-1.13, 3.16)	0.228	43.7	⊕⊖⊖⊖

MD, mean difference; CI, confidence interval. Except for muscle mass, a negative MD indicated a greater reduction in the HIIT group than in the MICT group. * indicated statistical significance (P < 0.05). The GRADE certainty of evidence was represented by ⊕/⊖, with ⊕⊕⊕⊕、⊕⊕⊕⊖、⊕⊕⊖⊖, and ⊕⊖⊖⊖ indicating high-, moderate-, low-, and very low-quality evidence, respectively.

**Table 3:** Meta-Analysis Results of the Effects of HIIT and MICT on Glucose and Lipid Metabolism Indicators.

Outcome	k	n	MD (95% CI)	p-value	I^2^ (%)	GRADE
Total cholesterol (mmol/L)	5	155	0.03 (-0.37, 0.42)	0.860	41.5	⊕⊖⊖⊖
Triglycerides (mmol/L)	6	198	0.002 (-0.13, 0.14)	0.971	0	⊕⊖⊖⊖
HDL-C (mmol/L)	5	174	0.02 (-0.04, 0.08)	0.475	0	⊕⊖⊖⊖
LDL-C (mmol/L)	5	174	-0.03 (-0.08, 0.03)	0.225	0	⊕⊖⊖⊖
Fasting blood glucose (mmol/L)	5	149	0.002 (-0.408, 0.411)	0.992	78.7	⊕⊖⊖⊖
Fasting insulin (μIU/mL)	4	109	-1.05 (-3.60, 1.50)	0.280	37.0	⊕⊖⊖⊖

MD, mean difference; CI, confidence interval. HDL-C, high-density lipoprotein cholesterol; LDL-C, low-density lipoprotein cholesterol. Except for HDL-C, a negative MD indicated a greater reduction in the HIIT group than in the MICT group. The GRADE certainty of evidence was represented by ⊕/⊖, with ⊕⊕⊕⊕, ⊕⊕⊕⊖, ⊕⊕⊖⊖, and ⊕⊖⊖⊖ indicating high-, moderate-, low-, and very low-quality evidence, respectively.

## Meta-analysis results

### Effects of HIIT and MICT on body composition-related indicators in college students

Eight outcomes related to body composition were evaluated (**Table 2**). Specifically, body weight, BMI, body fat percentage, waist-to-hip ratio, waist circumference, hip circumference, fat mass, and muscle mass were included in 15 trials (n = 487), 19 trials (n = 720), 14 trials (n = 465), 7 trials (n = 227), 7 trials (n = 240), 6 trials (n = 197), 8 trials (n = 240), and 4 trials (n = 121), respectively. Compared to MICT, HIIT significantly decreased body weight (MD = -1.23 kg, 95% CI -1.94 to -0.52; P = 0.002; I^2^ = 0.0%), body fat percentage (MD = -1.21%, 95% CI -2.12 to -0.30; P = 0.013; I^2^ = 77.8%), waist-to-hip ratio (MD = -0.01, 95% CI -0.02 to -0.00; P = 0.014; I^2^ = 0.0%), waist circumference (MD = -1.33 cm, 95% CI -2.40 to -0.26; P = 0.023; I^2^ = 0.0%), and fat mass (MD = -0.99 kg, 95% CI -1.66 to -0.33; P = 0.010; I^2^ = 0.0%). No statistically significant differences were observed between the two groups in BMI, hip circumference, or muscle mass. The quality assessment of the evidence indicated that the certainty was moderate for body weight, waist-to-hip ratio, waist circumference, and fat mass; low for BMI and body fat percentage; and very low for hip circumference and muscle mass. The forest plots for each outcome are provided in **Supplementary File** (**Supplementary Figures 1–8**).

To assess the robustness of the results, leave-one-out sensitivity analyses were performed for BMI, body fat percentage, and muscle mass. The findings showed that the result for body fat percentage was relatively stable. Specifically, after excluding the study by Song et al. (2024) (48), the pooled effect remained statistically significant (MD = -0.80%, 95% CI -1.37 to -0.24, P = 0.009), and heterogeneity dropped to 0.0%. In contrast, the pooled results for BMI changed from non-significant to significant after excluding studies by Gao et al. (2017) (36), Yang (2024) (50), Xiao (2021) (42), and Guo et al. (2025) (54). Additionally, the finding for muscle mass was also not sufficiently stable after excluding Zhao et al. (2020) (38); the pooled effect became significant (MD = 0.54, 95% CI 0.08 to 0.99, P = 0.037), and heterogeneity decreased to 0.0%. These findings indicate that the result for body fat percentage is relatively robust, while the conclusions regarding BMI and muscle mass should be interpreted with caution. The sensitivity analysis results are detailed in **Supplementary File** (**Supplementary Figures 47–49**).

### Effects of HIIT and MICT on glucose and lipid metabolism-related indicators in college students

This study assessed six outcomes related to glucose and lipid metabolism (**Table 3**), including four indicators of lipid metabolism and two indicators of glucose metabolism. Specifically, TC, TG, HDL-C, LDL-C, FBG, and FINS were measured across five trials (n = 155), six trials (n = 198), five trials (n = 174), five trials (n = 174), five trials (n = 149), and four trials (n = 109), respectively. Compared to MICT, the effects of HIIT on TC, TG, HDL-C, LDL-C, FBG, and FINS were not statistically significant (P > 0.05), and the GRADE certainty of evidence for all outcomes was rated as very low. The forest plots for each outcome are in **Supplementary File** (**Supplementary Figures 9–14**).

For outcomes showing high heterogeneity, such as TC, FBG, and FINS, leave-one-out sensitivity analyses were performed, as detailed in **Supplementary File** (**Supplementary Figures 50–52**). The statistical conclusions for all outcomes stayed consistent after these analyses. Notably, for FBG, heterogeneity dropped to 0.0% when the study by Yang et al. (2022) (45). Therefore, these results should be viewed with caution.

## Subgroup analysis results

### Body composition indicators

Subgroup analyses were performed on seven body composition indicators (**Table 4**). For body weight, the only statistically significant difference between subgroups was observed when stratified by sex (P for interaction < 0.001), with a notable decrease mainly in the male subgroup (P < 0.001). Similarly, regarding BMI, a significant difference between subgroups was observed only in the sex-stratified analysis (P for interaction = 0.017), with the effect mainly evident in the male subgroup (P = 0.024). Significant reductions in body fat percentage were observed in subgroups of students with obesity, female students, those with an intervention duration exceeding 8 weeks, and a training frequency of more than 3 sessions per week; however, tests for differences between subgroups across all stratification factors did not reach statistical significance. Although some subgroups showed significant reductions in waist-to-hip ratio and waist circumference, the tests for differences between these subgroups were also not statistically significant. For hip circumference, the combined effects across all subgroups also did not reach statistical significance. Although the test for differences between subgroups stratified by sex showed a statistically significant result (P for interaction = 0.011), this finding should be interpreted with caution. Significant reductions in fat mass were observed in subgroups of students with obesity, those with an intervention duration of more than 8 weeks, and those with a training frequency of more than 3 sessions per week. However, tests for differences between subgroups across all stratification factors did not reach statistical significance. The forest plots for each subgroup analysis are included in **Supplementary File** (**Supplementary Figure 15–36**).

**Table 4:** Subgroup Analysis Results for Body Composition Indicators.

Outcome	Subgroup factor	Subgroup level	k	MD (95% CI)	p-value	I^2^ (%)	P for interaction
Body weight (kg)	Weight status	Overweight	7	-0.59 (-1.94, 0.76)	0.323	0.0	0.231
		Obesity	8	-1.42 (-2.41, -0.43)	0.011*	2.0	
	Sex	Male	6	-2.14 (-2.86, -1.42)	<0.001*	0.0	<0.001
		Female	9	-0.57 (-1.30, 0.17)	0.112	0.0	
	Intervention duration	≤ 8 weeks	4	-1.23 (-1.92, -0.54)	0.011*	0.0	0.677
		> 8 weeks	11	-1.02 (-2.05, 0.02)	0.053	2.7	
	Training frequency	≤ 3 sessions/week	5	-0.96 (-1.86, -0.06)	0.042*	0.0	0.430
		> 3 sessions/week	10	-1.42 (-2.52, -0.32)	0.017*	0.0	
BMI (kg/m^2^)	Weight status	Overweight	7	-0.39 (-1.23, 0.45)	0.304	60.9	0.992
		Obesity	8	-0.34 (-0.78, 0.09)	0.106	7.5	
		Normal weight	3	-0.36 (-0.91, 0.19)	0.105	0.0	
	Sex	Male	7	-0.75 (-1.37, -0.14)	0.024*	39.8	0.017
		Female	11	-0.09 (-0.36, 0.17)	0.451	0.0	
	Intervention duration	≤ 8 weeks	6	-0.33 (-0.50, -0.17)	0.003*	0.0	0.817
		> 8 weeks	13	-0.28 (-0.75, 0.18)	0.212	51.7	
	Training frequency	≤ 3 sessions/week	9	-0.21 (-0.44, 0.01)	0.062	0.0	0.462
		> 3 sessions/week	10	-0.42 (-1.03, 0.18)	0.147	54.0	
Body fat percentage (%)	Weight status	Overweight	6	-0.63 (-1.37, 0.12)	0.083	0.0	0.087
		Obesity	6	-1.99 (-3.89, -0.09)	0.043*	82.6	
	Sex	Male	4	-1.44 (-2.94, 0.05)	0.054	0.0	0.675
		Female	9	-1.79 (-3.39, -0.20)	0.032*	87.1	
	Intervention duration	≤ 8 weeks	4	-1.59 (-5.80, 2.62)	0.315	87.1	0.605
		> 8 weeks	10	-0.89 (-1.58, -0.20)	0.017*	2.4	
	Training frequency	≤ 3 sessions/week	6	-1.10 (-3.35, 1.16)	0.267	90.6	0.688
		> 3 sessions/week	8	-1.47 (-2.12, -0.81)	0.001*	0.0	
Waist-to-hip ratio	Sex	Male	4	-0.02 (-0.03, -0.01)	0.007*	0.0	0.433
		Female	3	-0.01 (-0.04, 0.02)	0.247	0.0	
	Intervention duration	≤ 8 weeks	2	-0.01 (-0.04, 0.02)	0.117	0.0	0.698
		> 8 weeks	5	-0.01 (-0.02, 0.00)	0.087	0.0	
Waist circumference (cm)	Sex	Male	4	-1.30 (-2.43, -0.18)	0.034*	0.0	0.384
		Female	3	-1.80 (-3.72, 0.12)	0.056	0.0	
	Intervention duration	≤ 8 weeks	3	-1.17 (-2.25, -0.08)	0.044*	0.0	0.737
		> 8 weeks	4	-1.45 (-3.96, 1.06)	0.164	0.0	
Hip circumference (cm)	Sex	Male	2	-1.13 (-6.92, 4.66)	0.244	0.0	0.011
		Female	2	0.09 (-1.95, 2.14)	0.662	0.0	
	Intervention duration	≤ 8 weeks	2	-0.06 (-3.20, 3.07)	0.842	0.0	0.400
		> 8 weeks	4	-0.67 (-2.84, 1.49)	0.396	0.0	
Fat mass (kg)	Weight status	Overweight	4	-0.47 (-1.59, 0.66)	0.419	0.0	0.375
		Obesity	2	-1.11 (-1.98, -0.24)	0.012*	0.0	
	Sex	Male	2	-1.06 (-2.49, 0.37)	0.067	0.0	0.314
		Female	4	-0.67 (-1.86, 0.52)	0.172	0.0	
	Intervention duration	≤ 8 weeks	2	-1.59 (-23.16, 19.98)	0.522	43.9	0.676
		> 8 weeks	6	-0.87 (-1.44, -0.30)	0.011*	0.0	
	Training frequency	≤ 3 sessions/week	4	-0.76 (-3.06, 1.55)	0.374	29.6	0.614
		> 3 sessions/week	4	-1.13 (-1.64, -0.62)	0.006*	0.0	

P for interaction indicated the P value for the test of between-subgroup differences. MD, mean difference; CI, confidence interval. * indicated that the within-subgroup difference was statistically significant (P < 0.05).

### Glucose and lipid metabolism indicators

Subgroup analyses were conducted on six indicators of glucose and lipid metabolism. The results showed that TC, TG, HDL-C, LDL-C, and FINS did not demonstrate significant effects in any of the analyzed subgroups. Additionally, the subgroup difference tests were not statistically significant (see **Table 5**). Regarding FBG, stratification by weight status showed no significant effects within subgroups or differences between them. Although the test for differences between subgroups stratified by sex was statistically significant (P for interaction < 0.001), the combined effects in both male (P = 0.150) and female (P = 0.155) subgroups did not reach significance. The forest plots for each subgroup analysis are included in **Supplementary File** (**Supplementary Figures 37–46**).

**Table 5:** Subgroup Analysis Results for Glucose and Lipid Metabolism Indicators.

Outcome	Subgroup factor	Subgroup level	k	MD (95% CI)	p-value	I^2^ (%)	P for interaction
TC (mmol/L)	Weight status	Overweight	2	0.06 (-0.84, 0.97)	0.545	0.0	0.632
		Obesity	3	-0.08 (-1.30, 1.15)	0.808	69.7	
	Sex	Male	2	-0.25 (-5.77, 5.27)	0.666	75.9	0.361
		Female	2	0.16 (-1.55, 1.88)	0.437	0.0	
TG (mmol/L)	Weight status	Overweight	2	0.00 (-1.19, 1.20)	0.968	0.0	0.974
		Obesity	4	0.00 (-0.23, 0.23)	0.992	22.2	
	Sex	Male	3	-0.11 (-0.60, 0.38)	0.440	36.8	0.180
		Female	2	0.04 (-0.02, 0.11)	0.071	0.0	
HDL-C (mmol/L)	Sex	Male	2	0.03 (-0.33, 0.39)	0.480	0.0	0.870
		Female	2	0.02 (-0.57, 0.61)	0.726	0.0	
LDL-C (mmol/L)	Sex	Male	2	-0.05 (-0.45, 0.36)	0.387	0.0	0.650
		Female	2	-0.02 (-0.52, 0.48)	0.668	0.0	
FBG (mmol/L)	Weight status	Overweight	2	-0.05 (-4.94, 4.84)	0.914	94.4	0.825
		Obesity	3	0.03 (-0.19, 0.25)	0.578	0.0	
	Sex	Male	2	-0.42 (-1.70, 0.86)	0.150	0.0	<0.001
		Female	3	0.22 (-0.20, 0.63)	0.155	19.7	
FINS (μIU/mL)	Weight status	Overweight	2	-1.27 (-14.57, 12.03)	0.439	15.5	0.955
		Obesity	2	-1.38 (-23.91, 21.14)	0.578	68.4	
	Sex	Male	2	-1.62 (-25.02, 21.77)	0.540	55.1	0.714
		Female	2	-0.85 (-14.09, 12.39)	0.565	54.2	

P for interaction indicated the P value for the test of between-subgroup differences. MD, mean difference; CI, confidence interval. TC: Total cholesterol. TG: triglycerides. HDL-C: high-density lipoprotein cholesterol. LDL-C: low-density lipoprotein cholesterol. FBG: fasting blood glucose. FINS: fasting insulin.

### Meta-regression analyses

**Figure 3:**
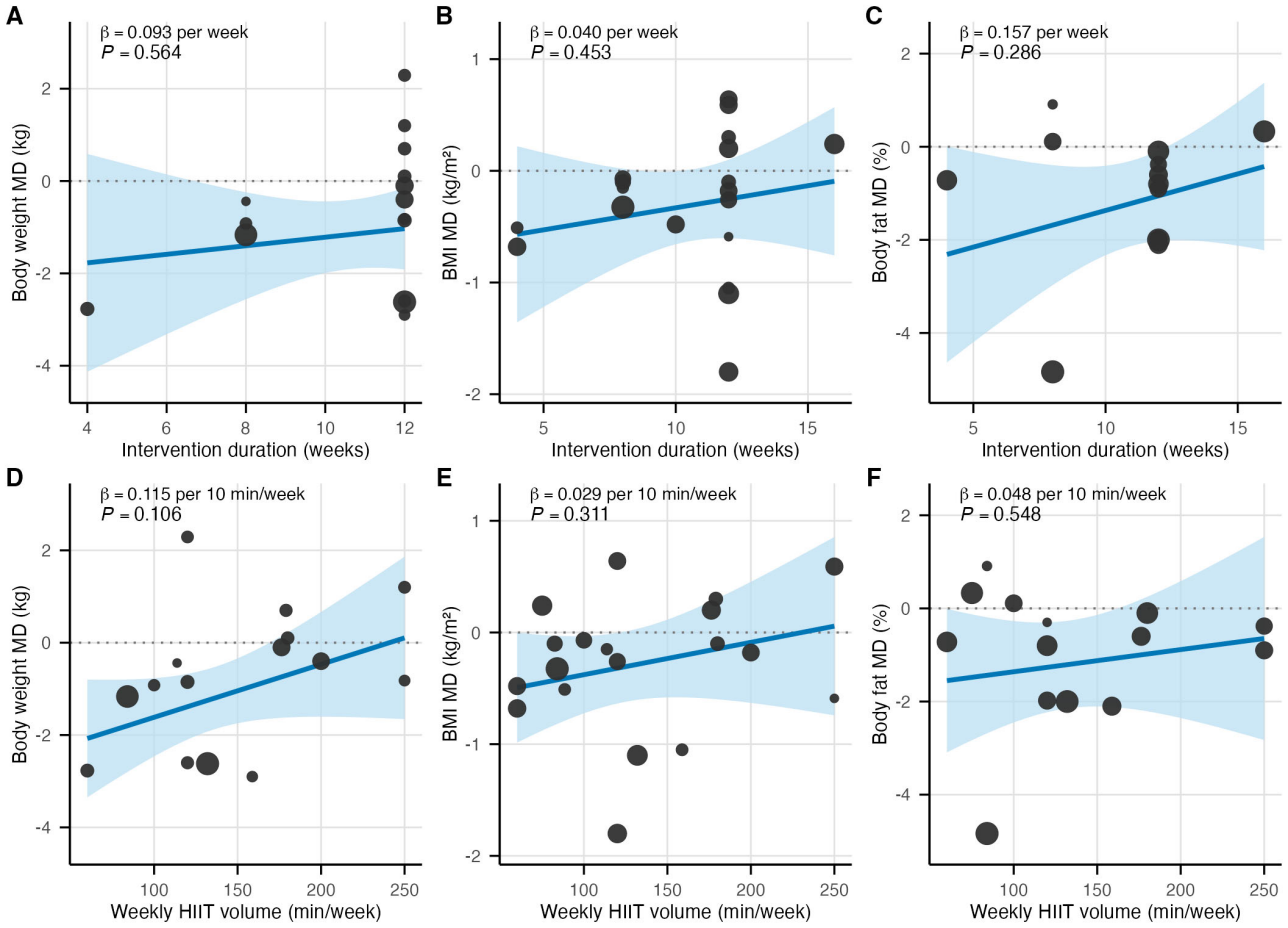
Meta-regression of intervention duration and weekly HIIT volume. **(A–C)** show intervention duration, and panels **(D–F)** show weekly HIIT volume. Bubble size reflects study weight; lines and shaded areas represent fitted effects and 95% CIs.

We conducted separate meta-regression analyses for body weight, BMI, and body fat percentage. The results showed that intervention duration did not significantly moderate the comparative effects of HIIT versus MICT on body weight (β = 0.093, 95% CI -0.25 to 0.43, P = 0.564), BMI (β = 0.040, 95% CI -0.07 to 0.15, P = 0.453), or body fat percentage (β = 0.157, 95% CI -0.15 to 0.46, P = 0.286). Weekly HIIT training volume also had no significant moderating effect on body weight (β = 0.115, 95% CI -0.03 to 0.26, P = 0.106), BMI (β = 0.029, 95% CI -0.03 to 0.09, P = 0.311), or body fat percentage (β = 0.048, 95% CI -0.12 to 0.22, P = 0.548) (**Figure 3**).

### Publication bias

**Figure 4:**
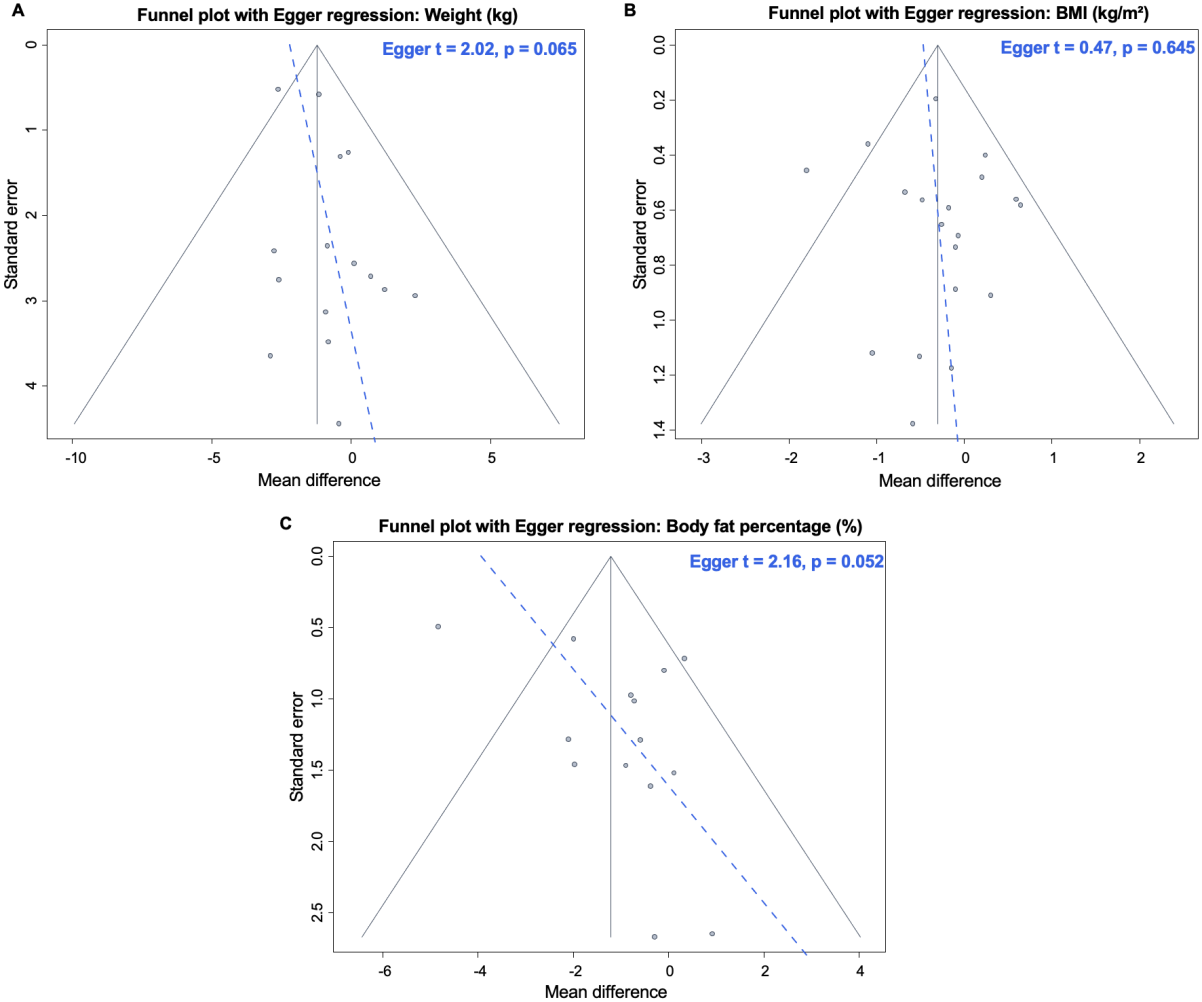
Funnel plots for body weight, BMI, and body fat percentage outcomes. **(A)** body weight; **(B)** BMI; **(C)** body fat percentage.

For outcomes with ten or more included studies, no significant publication bias was indicated for body weight (t = 2.015, P = 0.065), BMI (t = 0.469, P = 0.645), or body fat percentage (t = 2.160, P = 0.052) (**Figure 4**). In contrast, for the remaining outcomes, which included fewer than ten studies, the results of Egger’s test were used solely as exploratory references (**Supplementary Figure 53**). Among these outcomes, Egger’s test showed a statistically significant result for TG (t = -4.833, P = 0.008), indicating the possible presence of small-study effects (k = 6) or publication bias. No significant publication bias was found for other indicators, including waist-to-hip ratio, waist circumference, hip circumference, fat mass, muscle mass, TC, HDL-C, LDL-C, FBG, and FINS.

### Sensitivity analysis

Correlation coefficient sensitivity analyses indicated that, except for BMI, the statistical conclusions for the other 13 indicators—including body weight, body fat percentage, waist-to-hip ratio, waist circumference, hip circumference, fat mass, muscle mass, TC, TG, HDL-C, LDL-C, FBG, and FINS—remained consistent across three settings of r = 0.25, 0.50, and 0.75. BMI reached statistical significance when r was set at 0.25 (MD = -0.34, 95% CI -0.65 to -0.03; P = 0.033) but did not reach significance at r = 0.50 (P = 0.055) or r = 0.75 (P = 0.076), indicating that the BMI result was somewhat sensitive to the correlation coefficient assumption. Detailed data are provided in **Supplementary File S3**.

## Discussion

This study offers a thorough, systematic comparison of how HIIT and MICT affect body composition and indicators of glucose and lipid metabolism among college students. A total of 745 college students were included in the analysis, which examined 14 outcomes: 8 body composition indicators and 6 indicators of glucose and lipid metabolism. Compared to MICT, HIIT led to significant reductions in body weight, body fat percentage, waist-to-hip ratio, waist circumference, and fat mass. However, the differences in BMI, hip circumference, and muscle mass were not statistically significant. Regarding glucose and lipid metabolism, no significant differences were observed between HIIT and MICT for TC, TG, HDL-C, LDL-C, FBG, or FINS. Subgroup analyses suggested that sex might influence certain body composition outcomes, with notable differences in body weight and BMI when separated by sex; however, most subgroup differences in body composition and glucose and lipid metabolism measures were not statistically significant. Meta-regression further showed that neither intervention duration nor weekly HIIT training volume significantly moderated the effects on body weight, BMI, or body fat percentage. In summary, the additional benefits of HIIT over MICT mainly appeared in fat-related body composition and abdominal measurements. At the same time, no consistent advantages were observed for BMI, muscle mass, or routine glucose and lipid metabolism indicators.

### Body composition-related indicators

This study found that, among college students, HIIT significantly decreased body weight (MD = -1.23 kg, P = 0.002), body fat percentage (MD = -1.21%, P = 0.013), waist circumference (MD = -1.33 cm, P = 0.023), waist-to-hip ratio (MD = -0.01, P = 0.014), and fat mass (MD = -0.99 kg, P = 0.010) compared to MICT. In contrast, BMI showed only a borderline significant trend (MD = -0.31 kg/m^2^, P = 0.055), and the differences between groups in hip circumference and muscle mass were not statistically significant. These findings suggest that the advantages of HIIT over MICT are not consistently reflected across all body composition measures, but are mainly evident in measures related to fat mass and abdominal fat distribution.

This finding agrees with previous research. A meta-analysis by Wewege et al. (2017) (21) found that, among adults with overweight and obesity, both HIIT and MICT improved body composition; however, HIIT did not consistently demonstrate advantages across all body composition measures. Similarly, the meta-analysis by Su et al. (2019) (55) indicated that HIIT and MICT produced generally similar improvements in body composition metrics, including body weight, BMI, and body fat percentage. Notably, each HIIT session lasted about 9.7 minutes less than an MICT session, suggesting that HIIT’s main advantage may be its time efficiency rather than superior fat loss. Unlike these earlier studies, the current research focuses on college students, a group characterized by a younger age and relatively healthy metabolic function. As a result, changes in body composition might precede shifts in metabolic indicators such as blood glucose and lipids. Additionally, because some participants had baseline BMI or body fat levels that were not significantly abnormal, changes in general or nonspecific indicators such as BMI, hip circumference, and muscle mass may have been less sensitive compared to changes in fat mass, body fat percentage, and waist circumference.

BMI has inherent measurement limitations because it does not distinguish between fat mass, lean mass, and changes in body fluid (56, 57). When fat mass decreases while muscle mass remains stable or slightly increases, reductions in body weight and BMI may be partially offset. In contrast, metrics such as body fat percentage, fat mass, waist circumference, and waist-to-hip ratio offer a more direct reflection of changes in body composition and fat distribution (58). Okorodudu et al. (2010) (59) observed that BMI has limited sensitivity in detecting high levels of body fat, and Ross et al. (2020) (60) further emphasized that waist circumference is a superior indicator of abdominal fat accumulation and related metabolic risk, which cannot be fully reflected by BMI alone.

From a physiological perspective, these changes may be explained by increased post-exercise energy expenditure, enhanced fat mobilization, and adaptations in skeletal muscle oxidative metabolism. High-intensity exercise can sustain elevated energy expenditure during recovery (61), while low-volume HIIT promotes mitochondrial and metabolic adaptations in skeletal muscle (13). At the cellular level, repeated energetic stress may activate AMPK-related signaling. Broader evidence suggests that AMPK-mTOR crosstalk coordinates mitochondrial remodeling, autophagy, and proteostasis, while also regulating transcriptional programs related to cellular growth and metabolism (71, 72). These responses may enhance oxidative capacity and tissue remodeling. However, body composition responses also depend on total training volume, intervention duration, dietary intake, initial body weight, and adherence. Therefore, our findings suggest a potential advantage of HIIT for selected fat-related and abdominal measures rather than superiority across all body composition outcomes. Future studies should standardize training-dose reporting, assess adherence, and include direct measures of visceral fat to clarify the comparative effects of HIIT and MICT.

From a dietary perspective, the interpretation of body composition outcomes should consider the influence of energy balance and nutrient intake (73, 74). Previous research indicates that the balance between total energy intake and energy expenditure underlies changes in body weight and fat mass, while dietary composition, particularly protein intake, may influence the extent of fat loss and fat-free mass preservation during weight loss (74, 75). Meta-analytic evidence further indicates that, under energy-restricted conditions, higher-protein diets produce greater reductions in fat mass and better preserve fat-free mass than standard-protein diets (75, 76). However, dietary intake was not consistently assessed or controlled across the included trials, and participant-level dietary data were unavailable. Therefore, the potential influence of differences in energy or macronutrient intake on body composition outcomes could not be excluded. This limitation may have introduced residual confounding into the comparison of HIIT and MICT. Future studies should incorporate systematic dietary assessments and, where feasible, control or statistically adjust for total energy and macronutrient intake.

Subgroup analyses suggested that sex may influence the relative effects of HIIT versus MICT on body weight and BMI. However, most subgroup differences in body composition and glucose and lipid metabolism were not statistically significant, and several subgroups included only 2–4 studies. These findings should therefore be interpreted cautiously. Meta-regression showed that neither intervention duration nor weekly HIIT training volume significantly moderated the effects on body weight, BMI, or body fat percentage. However, the limited number of studies and the inability to examine exercise intensity, modality, and work-to-rest ratio preclude firm conclusions regarding potential moderators or dose-response relationships.

### Glucose metabolism-related indicators

This meta-analysis shows that, compared to MICT, HIIT does not have significant benefits for glucose metabolism-related indicators. Specifically, the differences between groups in FBG (MD = 0.00 mmol/L, P = 0.992) and FINS (MD = -1.05 μIU/mL, P = 0.280) were not statistically significant. Previous research on the effects of HIIT and MICT on glucose metabolism has shown inconsistent results. This inconsistency may result from differences in training protocols, intervention duration, exercise dosage, and baseline metabolic status across study populations. In individuals with type 2 diabetes or prediabetes, some studies suggest that HIIT or interval training can provide greater benefits than continuous training for improving glucose metabolism. For example, Karstoft et al. (2014) (62) demonstrated that interval walking training significantly improved insulin sensitivity and peripheral glucose disposal compared to continuous walking training in patients with type 2 diabetes. Furthermore, the meta-analysis conducted by Liubaoerjijin et al. (2016) (63) indicated that higher-intensity exercise may lead to greater improvements in glycated hemoglobin (HbA1c) among patients with type 2 diabetes. Conversely, other studies have suggested that HIIT does not consistently outperform MICT in glucose metabolism. De Nardi et al. (2018) (64) found no significant differences between HIIT and MICT in glucose metabolism indicators, such as HbA1c, in their meta-analysis of individuals with prediabetes and type 2 diabetes. Similarly, Mateo-Gallego et al. (2022) (65) reported that while HIIT improved blood glucose levels, insulin, and the homeostatic model assessment of insulin resistance (HOMA-IR) in diabetic patients, it did not show a consistent benefit over MICT.

From a glucose-regulation perspective, repeated high-intensity contractions may transiently activate AMPK, while short-term HIIT can increase skeletal muscle GLUT4 content and improve glucose control (13, 66). Beyond skeletal muscle, amino acid-Rab1A-mTORC1-PDX1 signaling regulates insulin expression and beta-cell function, linking nutrient sensing to systemic glucose control (77, 78). These pathways provide a possible biological explanation for HIIT-related adaptations in glucose regulation. However, this interpretation remains tentative because the included studies did not directly assess the relevant molecular signaling pathways.

These mechanistic adaptations may not necessarily lead to significant short-term reductions in FBG or FINS, especially among young populations with normal baseline glycemic regulation. Unlike those with metabolic disorders, most college students included in this study did not show clear abnormalities in glucose metabolism. Therefore, the absence of significant differences in this study does not mean that HIIT is ineffective at affecting glucose metabolism; rather, static measures such as FBG and FINS may not be sensitive enough to detect early or mild metabolic changes in college students. Subgroup analyses showed a notable difference in FBG levels between sexes; however, within-subgroup effects in both males and females did not reach statistical significance. Additionally, FINS did not show significant differences across subgroups defined by weight status or sex. Therefore, the current results do not provide enough evidence to support a clear moderating role of sex or weight status on the effects of HIIT and MICT on glucose metabolism. Considering the limited number of studies exploring glucose metabolism outcomes and the relatively high overall heterogeneity observed for FBG, these subgroup findings should be interpreted cautiously. Future research should seek to clarify participants’ baseline glucose metabolic status and include more sensitive indicators, such as the homeostasis model assessment of insulin resistance (HOMA-IR), the oral glucose tolerance test (OGTT), or continuous glucose monitoring, to better assess the differential effects of HIIT and MICT on glucose metabolism among college students.

### Lipid metabolism-related indicators

This meta-analysis found that, compared to MICT, HIIT did not show consistent benefits in lipid metabolism-related indicators. Specifically, the differences between groups in TC (MD = 0.03 mmol/L, P = 0.860), TG (MD = 0.00 mmol/L, P = 0.971), HDL-C (MD = 0.02 mmol/L, P = 0.475), and LDL-C (MD = -0.03 mmol/L, P = 0.225) were not statistically significant. This finding aligns with most studies that have directly compared HIIT and MICT. The systematic review and meta-analysis by Wood et al. (2019) (67) indicated that HIIT was not superior to MICT in improving blood lipid markers, including TC, TG, and LDL-C. Similarly, Su et al. (2019) (55) found in a meta-analysis of overweight or obese adults, both HIIT and MICT could improve certain cardiometabolic risk factors; however, the differences between the two training modalities regarding blood lipid indicators were inconsistent. Notably, Song et al. (2024) (48) observed that HIIT may produce more favorable effects on certain biochemical markers in college students with obesity, indicating that the impact of HIIT on lipid metabolism could be more significant in students with baseline obesity or higher metabolic risk. Therefore, the lack of significant differences between groups in the present study may be due to variations in baseline blood lipid levels, weight status, and the degree of metabolic risk among the participants included in the analysis.

**Figure 5:**
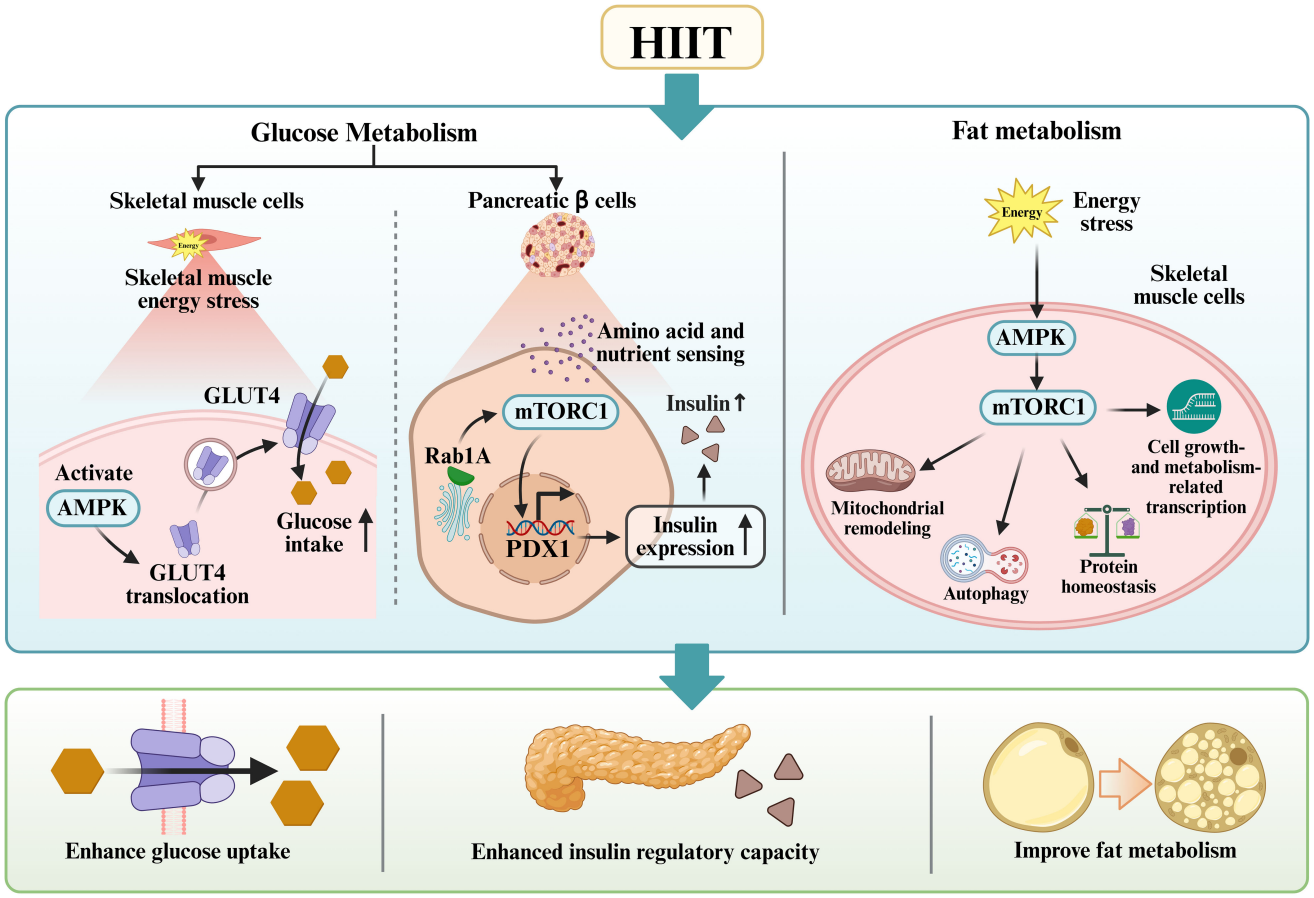
Proposed molecular mechanisms by which HIIT may influence glucose regulation and lipid metabolism.

The effects of exercise on lipid metabolism are not solely determined by exercise intensity; they are also influenced by overall training volume, energy expenditure, the degree of body fat reduction, and adaptations in lipoprotein metabolism. Kraus et al. (2002) (68) demonstrated that varying exercise volumes and intensities produce distinct effects on lipoprotein indicators, with a combination of higher volume and intensity more likely to lead to positive lipid changes. Exercise training may affect blood lipid levels by promoting fat oxidation, increasing lipoprotein lipase activity, improving lipoprotein particle metabolism, and decreasing fat mass (69). In this study, HIIT reduced body fat percentage, fat mass, and waist circumference, suggesting improvements in fat tissue and energy use. However, these changes in body composition may not have led to significant short-term differences in TC, TG, LDL-C, or HDL-C. Notably, increasing HDL-C usually requires a certain amount of exercise, and the improvement in HDL-C through aerobic activity is strongly linked to the total volume of exercise (70). Therefore, among college students, if baseline blood lipid levels are relatively normal, the intervention duration is short, or the total training volume is insufficient, HIIT may have limited ability to produce more significant changes in routine blood lipid indicators compared to MICT, even though it effectively improves fat-related body composition. Proposed molecular pathways through which HIIT may influence glucose regulation and lipid metabolism are summarized in **Figure 5**. Subgroup analyses did not show a significant moderating effect of weight status or sex on the lipid metabolism responses to HIIT and MICT. This indicates that current evidence is insufficient to confirm distinct differences in blood lipid responses across college student subgroups. Future research should examine the timing of blood lipid changes and the properties of lipoprotein subclasses to understand better how these two training methods differently affect lipid metabolism.

## Limitations

This study has several limitations. First, few studies were available for certain outcomes, particularly FBG, FINS, and some blood lipid indicators. This may have reduced the stability of the pooled estimates and the statistical power of subgroup analyses and publication bias tests. Second, some studies had small samples, while dietary control, daily physical activity, and lifestyle management were inconsistently reported. These factors may have introduced residual confounding into body composition and glucose and lipid metabolism outcomes. Third, subgroup analyses were based on study-level aggregate data, and some subgroups contained few studies with uneven distributions. Although intervention duration and weekly HIIT volume were examined using meta-regression, exercise intensity, modality, and work-rest ratio could not be modeled reliably because of inconsistent reporting and definitions. Therefore, the subgroup and meta-regression findings should be considered exploratory. Fourth, most studies did not adequately report baseline glycemic status, insulin resistance, or dyslipidemia; therefore, subgroup analyses by baseline metabolic status could not be performed. This may have obscured differences in glucose and lipid responses between metabolically healthy and metabolically impaired students. Finally, the study population was limited to college students, restricting the applicability of the findings to other age groups, populations with established metabolic diseases, and longer-term interventions.

## Conclusions

This study systematically compared the effects of HIIT and MICT on body composition and indicators of glucose and lipid metabolism in college students. Compared with MICT, HIIT was associated with greater reductions in body weight, body fat percentage, waist circumference, waist-to-hip ratio, and fat mass. No significant between-group differences were observed for BMI, hip circumference, muscle mass, FBG, FINS, TC, TG, HDL-C, or LDL-C. These findings suggest that any additional benefit of HIIT may be limited to selected adiposity-related outcomes. However, the subgroup and meta-regression findings were exploratory and did not provide robust evidence of effect modification, particularly because several subgroups included few studies. Current evidence remains insufficient to establish the superiority of HIIT for routine glucose and lipid metabolism indicators. Future research should standardize training-dose reporting and include baseline metabolic status, dietary control, adherence, and more sensitive metabolic markers.

## Data Availability

The original contributions presented in the study are included in the article/**Supplementary Material**. Further inquiries can be directed to the corresponding author.
