# Supplementary material for: Effects of high-intensity interval training and moderate-intensity continuous training on body composition and glucose and lipid metabolism in college students: a systematic review and meta-analysis
Source: Front Endocrinol (Lausanne). 2026 Jul 15;17:1894767. doi: 10.3389/fendo.2026.1894767 (PMC13414765; doi:10.3389/fendo.2026.1894767)

**Supplementary Material**

**Supplementary Table S1. Literature search strategy.**

| **Database** | **Literature Search Strategy** |
| --- | --- |
| PubMed | #1 ("High-Intensity Interval Training"[Mesh] OR "high-intensity interval training"[tiab] OR "high intensity interval training"[tiab] OR HIIT[tiab] OR "sprint interval training"[tiab] OR "high-intensity intermittent exercise"[tiab] OR "high-intensity intermittent training"[tiab] OR "aerobic interval training"[tiab] OR "interval training"[tiab]) #2 ("Endurance Training"[Mesh] OR "moderate-intensity continuous training"[tiab] OR "moderate intensity continuous training"[tiab] OR "moderate-intensity continuous exercise"[tiab] OR "moderate intensity continuous exercise"[tiab] OR MICT[tiab] OR "moderate continuous training"[tiab] OR "continuous aerobic training"[tiab] OR "continuous exercise"[tiab] OR "continuous training"[tiab] OR "aerobic continuous training"[tiab] OR "endurance training"[tiab]) #3 ("Students"[Mesh] OR "Young Adult"[Mesh] OR "Universities"[Mesh] OR student*[tiab] OR "college student*"[tiab] OR "university student*"[tiab] OR undergraduate*[tiab] OR "young adult*"[tiab] OR universit*[tiab]) #4 #1 AND #2 AND #3 |
| Web of Science | #1 TS=("high-intensity interval training" OR "high intensity interval training" OR HIIT OR "sprint interval training" OR "high-intensity intermittent exercise" OR "high-intensity intermittent training" OR "aerobic interval training" OR "interval training") #2 TS=("moderate-intensity continuous training" OR "moderate intensity continuous training" OR "moderate-intensity continuous exercise" OR "moderate intensity continuous exercise" OR MICT OR "moderate continuous training" OR "continuous aerobic training" OR "continuous exercise" OR "continuous training" OR "aerobic continuous training" OR "endurance training") #3 TS=(student* OR "college student*" OR "university student*" OR undergraduate* OR "young adult*" OR universit*) #4 #1 AND #2 AND #3 |
| Embase | #1 ('high-intensity interval training':ti,ab,kw OR 'high intensity interval training':ti,ab,kw OR HIIT:ti,ab,kw OR 'sprint interval training':ti,ab,kw OR 'high-intensity intermittent training':ti,ab,kw OR 'high-intensity intermittent exercise':ti,ab,kw OR 'aerobic interval training':ti,ab,kw OR 'interval training':ti,ab,kw) #2 ('moderate-intensity continuous training':ti,ab,kw OR 'moderate intensity continuous training':ti,ab,kw OR 'moderate-intensity continuous exercise':ti,ab,kw OR 'moderate intensity continuous exercise':ti,ab,kw OR MICT:ti,ab,kw OR 'moderate continuous training':ti,ab,kw OR 'continuous aerobic training':ti,ab,kw OR 'aerobic continuous training':ti,ab,kw OR 'continuous exercise':ti,ab,kw OR 'endurance training':ti,ab,kw) #3 ('college student':ti,ab,kw OR 'college students':ti,ab,kw OR 'university student':ti,ab,kw OR 'university students':ti,ab,kw OR undergraduate*:ti,ab,kw OR 'young adult':ti,ab,kw OR 'young adults':ti,ab,kw OR student*:ti,ab,kw) #4 #1 AND #2 AND #3 |
| Cochrane Library | #1 [mh "High-Intensity Interval Training"] OR "high-intensity interval training":ti,ab,kw OR "high intensity interval training":ti,ab,kw OR HIIT:ti,ab,kw OR "sprint interval training":ti,ab,kw OR "high-intensity intermittent exercise":ti,ab,kw OR "high-intensity intermittent training":ti,ab,kw OR "aerobic interval training":ti,ab,kw OR "interval training":ti,ab,kw #2 [mh "Endurance Training"] OR "moderate-intensity continuous training":ti,ab,kw OR "moderate intensity continuous training":ti,ab,kw OR "moderate-intensity continuous exercise":ti,ab,kw OR "moderate intensity continuous exercise":ti,ab,kw OR MICT:ti,ab,kw OR "moderate continuous training":ti,ab,kw OR "continuous aerobic training":ti,ab,kw OR "continuous exercise":ti,ab,kw OR "continuous training":ti,ab,kw OR "aerobic continuous training":ti,ab,kw OR "endurance training":ti,ab,kw #3 [mh Students] OR [mh "Young Adult"] OR [mh Universities] OR student*:ti,ab,kw OR (college NEXT student*):ti,ab,kw OR (university NEXT student*):ti,ab,kw OR undergraduate*:ti,ab,kw OR (young NEXT adult*):ti,ab,kw OR universit*:ti,ab,kw #4 #1 AND #2 AND #3 |
| CNKI | #1 高强度间歇 + HIIT + 间歇训练 #2 中等持续 + MICT + 持续训练 #3 大学生 + 高校学生 + 青年 + 学生 #4 #1 AND #2 AND #3 |
| VIP | #1 高强度间歇 + HIIT + 间歇训练 #2 中等持续 + MICT + 持续训练 #3 大学生 + 高校学生 + 青年 + 学生 #4 #1 AND #2 AND #3 |

Note: All databases were searched from inception to March 10, 2026.

**Supplementary Table S2. GRADE evidence quality assessment.**

| **Section** | **Outcome** | **Study Limitations** | **Inconsistencies** | **Indirectness** | **Imprecision** | **Publication Bias** | **GRADE** |
| --- | --- | --- | --- | --- | --- | --- | --- |
| Body morphology outcomes | Body weight | Downgrade 1 level^A | Not downgraded | Not downgraded | Not downgraded | Not downgraded | Moderate |
| Body morphology outcomes | BMI | Downgrade 1 level^A | Not downgraded | Not downgraded | Downgrade 1 level^C | Not downgraded | Low |
| Body morphology outcomes | Body fat percentage | Downgrade 1 level^A | Downgrade 1 level^B | Not downgraded | Not downgraded | Not downgraded | Low |
| Body morphology outcomes | Waist-to-hip ratio | Downgrade 1 level^A | Not downgraded | Not downgraded | Not downgraded | Not assessed^F | Moderate |
| Body morphology outcomes | Waist circumference | Downgrade 1 level^A | Not downgraded | Not downgraded | Not downgraded | Not assessed^F | Moderate |
| Body morphology outcomes | Hip circumference | Downgrade 1 level^A | Not downgraded | Not downgraded | Downgrade 2 levels^D | Not assessed^F | Very low |
| Body morphology outcomes | Fat mass | Downgrade 1 level^A | Not downgraded | Not downgraded | Not downgraded | Not assessed^F | Moderate |
| Body morphology outcomes | Muscle mass | Downgrade 1 level^A | Not downgraded | Not downgraded | Downgrade 2 levels^D | Not assessed^F | Very low |
| Glycolipid metabolism outcomes | Total cholesterol (TC) | Downgrade 1 level^A | Not downgraded | Not downgraded | Downgrade 2 levels^D | Not assessed^F | Very low |
| Glycolipid metabolism outcomes | Triglycerides (TG) | Downgrade 1 level^A | Not downgraded | Not downgraded | Downgrade 2 levels^D | Not assessed^F | Very low |
| Glycolipid metabolism outcomes | High-density lipoprotein cholesterol (HDL-C) | Downgrade 1 level^A | Not downgraded | Not downgraded | Downgrade 2 levels^D | Not assessed^F | Very low |
| Glycolipid metabolism outcomes | Low-density lipoprotein cholesterol (LDL-C) | Downgrade 1 level^A | Not downgraded | Not downgraded | Downgrade 2 levels^D | Not assessed^F | Very low |
| Glycolipid metabolism outcomes | Fasting blood glucose (FBG) | Downgrade 1 level^A | Downgrade 1 level^B | Not downgraded | Downgrade 2 levels^D | Not assessed^F | Very low |
| Glycolipid metabolism outcomes | Fasting insulin (FINS) | Downgrade 1 level^A | Not downgraded | Not downgraded | Downgrade 2 levels^D | Not assessed^F | Very low |

Notes.- ^A Most included studies were rated as some concerns or high risk of bias in the RoB 2 assessment.
- ^B Statistical heterogeneity was substantial (I² >= 50%).
- ^C The 95% confidence interval crossed the line of no effect.
- ^D The 95% confidence interval crossed the line of no effect and the total sample size was limited (<200 participants).
- ^E Clear evidence of publication bias or small-study effects from formal assessment.
- ^F Fewer than 10 studies were available; funnel plot and Egger's test were considered underpowered and were not used as a formal basis for downgrading.

**Supplementary Table S3. Sensitivity analysis of pooled effects under different assumed correlation coefficients.**

| **Outcome** | **r = 0.25** | **r = 0.50 (primary analysis)** | **r = 0.75** | **Change in statistical conclusion** |
| --- | --- | --- | --- | --- |
| Body weight (kg) | -1.37 (-2.04, -0.69) P < 0.001; I² = 0.0% | -1.23 (-1.94, -0.52) P = 0.002; I² = 0.0% | -1.02 (-1.77, -0.27) P = 0.012; I² = 17.8% | No |
| BMI (kg/m²) | -0.34 (-0.65, -0.03) P = 0.033; I² = 3.9% | -0.31 (-0.62, 0.01) P = 0.055; I² = 29.6% | -0.28 (-0.59, 0.03) P = 0.076; I² = 59.4% | Yes |
| Body fat percentage (%) | -1.28 (-2.22, -0.33) P = 0.012; I² = 74.6% | -1.21 (-2.12, -0.30) P = 0.013; I² = 77.8% | -1.12 (-1.98, -0.25) P = 0.016; I² = 81.6% | No |
| Waist-to-hip ratio | -0.01 (-0.02, -0.00) P = 0.010; I² = 0.0% | -0.01 (-0.02, -0.00) P = 0.014; I² = 0.0% | -0.01 (-0.02, -0.00) P = 0.039; I² = 0.0% | No |
| Waist circumference (cm) | -1.33 (-2.35, -0.31) P = 0.019; I² = 0.0% | -1.33 (-2.40, -0.26) P = 0.023; I² = 0.0% | -1.34 (-2.47, -0.21) P = 0.027; I² = 0.0% | No |
| Hip circumference (cm) | -0.18 (-0.93, 0.57) P = 0.561; I² = 0.0% | -0.24 (-1.08, 0.59) P = 0.491; I² = 0.0% | -0.36 (-1.32, 0.61) P = 0.389; I² = 0.0% | No |
| Fat mass (kg) | -1.01 (-1.62, -0.40) P = 0.006; I² = 0.0% | -0.99 (-1.66, -0.33) P = 0.010; I² = 0.0% | -0.99 (-1.78, -0.20) P = 0.021; I² = 28.4% | No |
| Muscle mass (kg) | 0.96 (-0.96, 2.88) P = 0.210; I² = 16.9% | 1.02 (-1.13, 3.16) P = 0.228; I² = 43.7% | 0.98 (-1.35, 3.31) P = 0.275; I² = 70.7% | No |
| Total cholesterol (TC, mmol/L) | 0.04 (-0.32, 0.41) P = 0.757; I² = 13.4% | 0.03 (-0.37, 0.42) P = 0.860; I² = 41.5% | -0.02 (-0.47, 0.43) P = 0.918; I² = 69.8% | No |
| Triglycerides (TG, mmol/L) | 0.00 (-0.14, 0.14) P = 0.987; I² = 0.0% | 0.00 (-0.13, 0.14) P = 0.971; I² = 0.0% | 0.00 (-0.12, 0.13) P = 0.926; I² = 38.3% | No |
| High-density lipoprotein cholesterol (HDL-C, mmol/L) | 0.02 (-0.04, 0.08) P = 0.467; I² = 0.0% | 0.02 (-0.04, 0.08) P = 0.475; I² = 0.0% | 0.02 (-0.04, 0.08) P = 0.495; I² = 0.0% | No |
| Low-density lipoprotein cholesterol (LDL-C, mmol/L) | -0.03 (-0.08, 0.03) P = 0.225; I² = 0.0% | -0.03 (-0.08, 0.03) P = 0.225; I² = 0.0% | -0.03 (-0.08, 0.03) P = 0.227; I² = 0.0% | No |
| Fasting blood glucose (FBG, mmol/L) | 0.01 (-0.40, 0.43) P = 0.946; I² = 70.1% | 0.00 (-0.41, 0.41) P = 0.992; I² = 78.7% | -0.01 (-0.41, 0.39) P = 0.946; I² = 87.3% | No |
| Fasting insulin (FINS, μIU/mL) | -0.94 (-3.39, 1.50) P = 0.308; I² = 11.4% | -1.05 (-3.60, 1.50) P = 0.280; I² = 37.0% | -1.18 (-3.84, 1.48) P = 0.254; I² = 62.7% | No |

Note: r denotes the assumed pre-post correlation coefficient used to estimate the standard deviation of change scores; r = 0.50 was used in the primary analysis. MD, mean difference; CI, confidence interval; I², heterogeneity statistic. For studies that directly reported the standard deviation of change scores or lacked complete pre- and post-intervention standard deviations, the reported/current change-score standard deviations were retained. Change in statistical conclusion indicates whether the judgment based on P < 0.05 differed from that of the primary analysis with r = 0.50.

**Supplementary Figures**

**Main analysis forest plots. Body morphology-related indicators S1-S8.**

**Supplementary Figure S1. Forest plot for the effect of HIIT on body weight.**


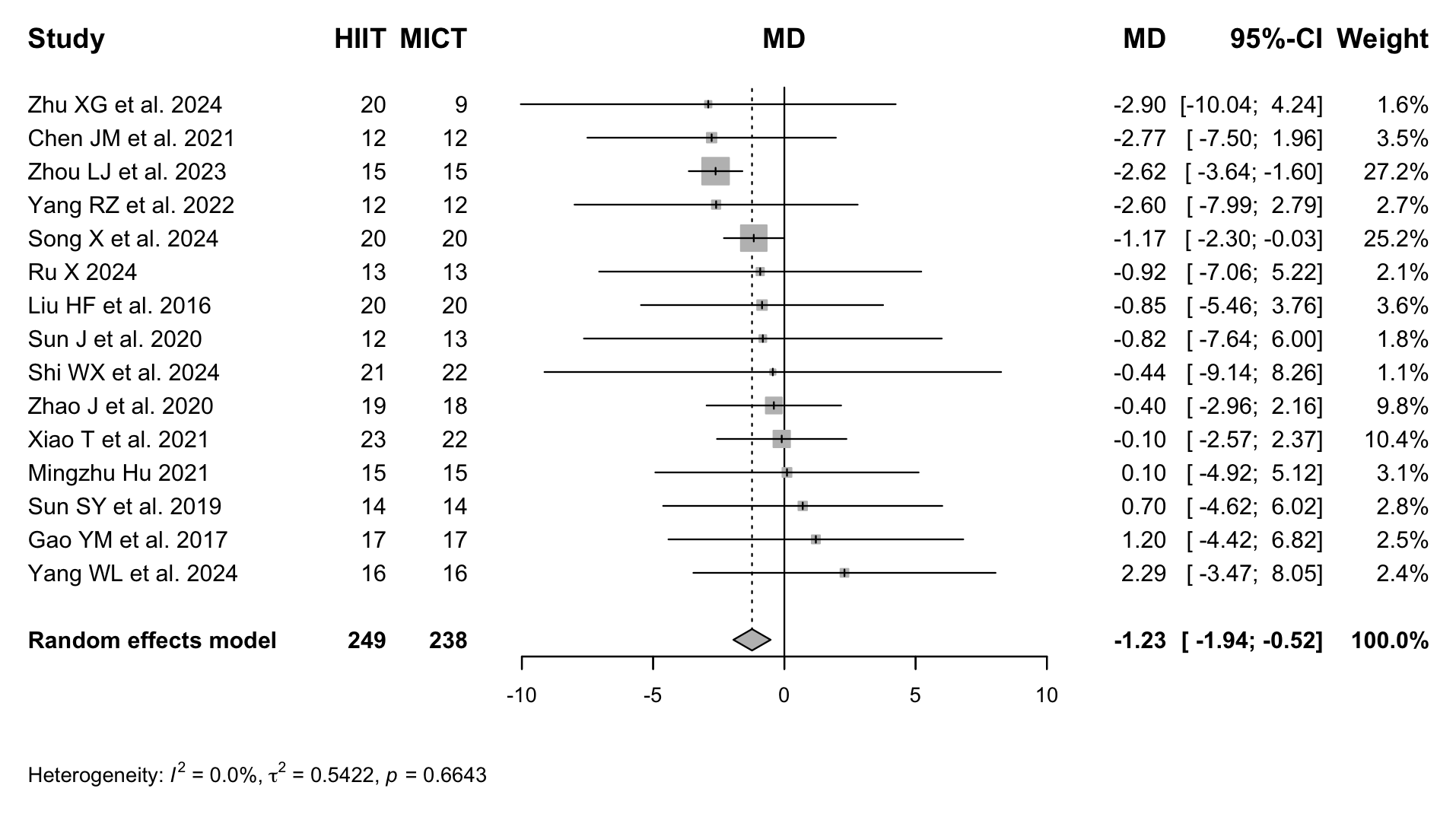


**Supplementary Figure S2. Forest plot for the effect of HIIT on body mass index (BMI).**


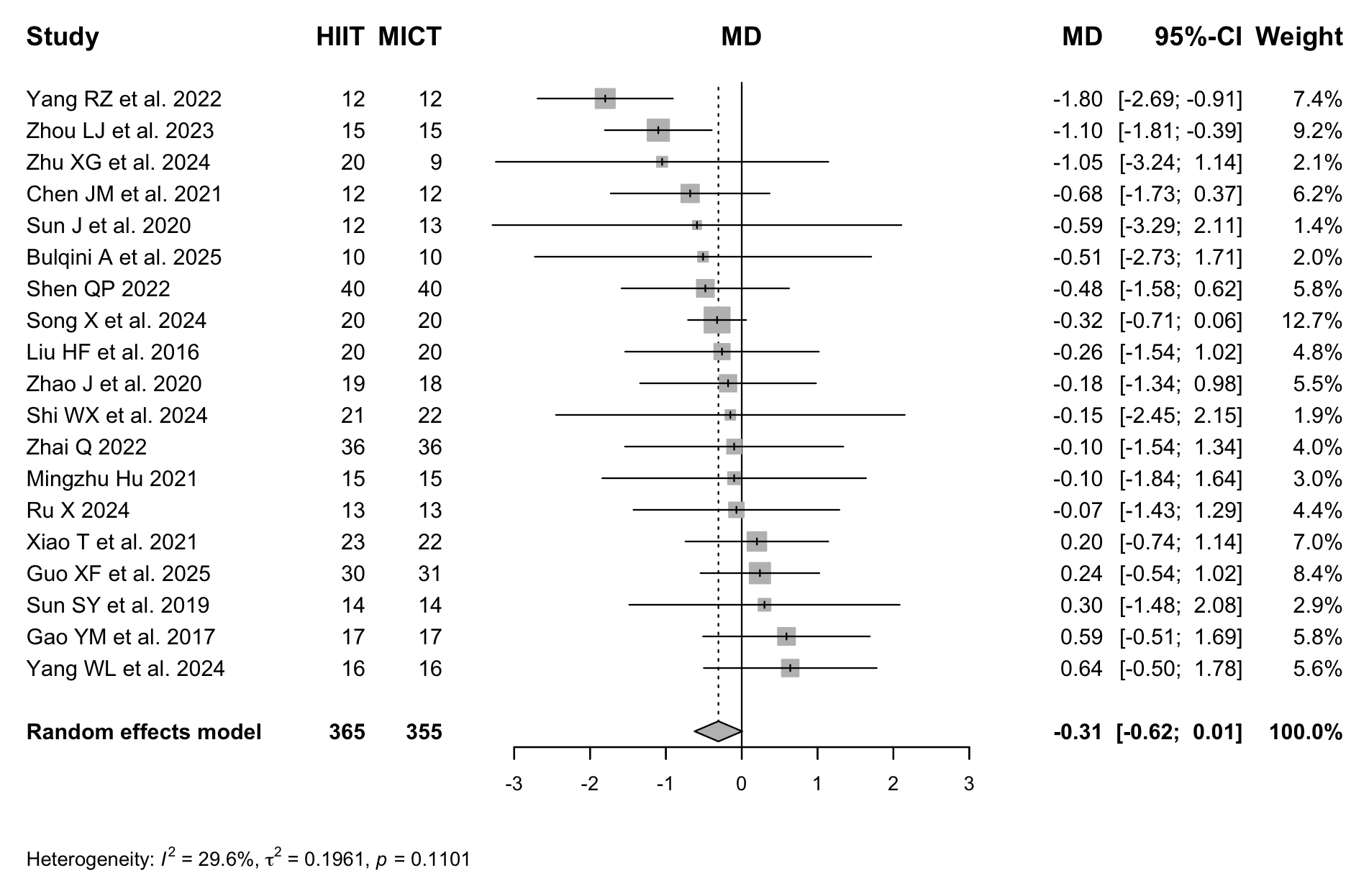


**Supplementary Figure S3. Forest plot for the effect of HIIT on body fat percentage.**


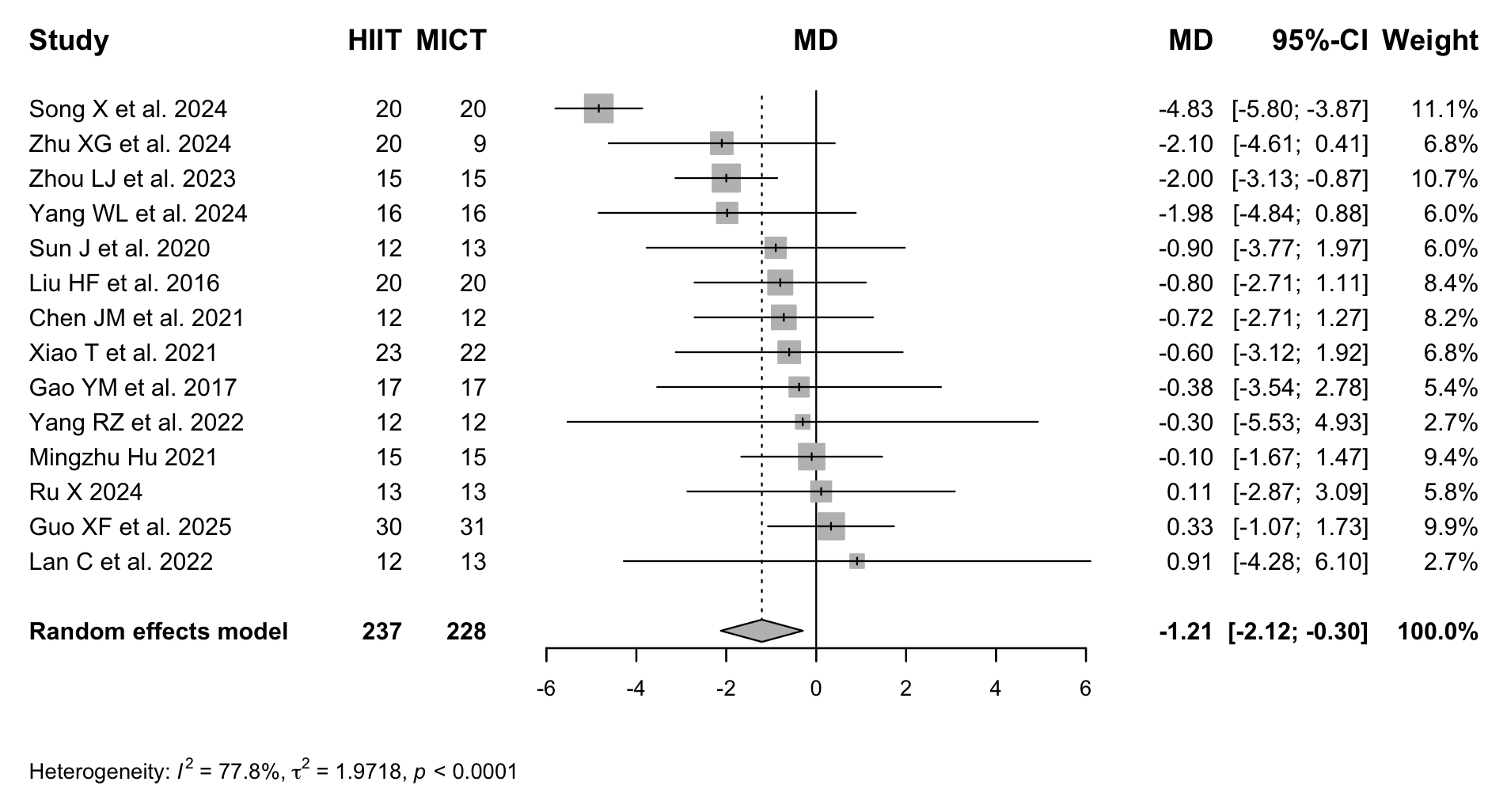


**Supplementary Figure S4. Forest plot for the effect of HIIT on fat mass.**


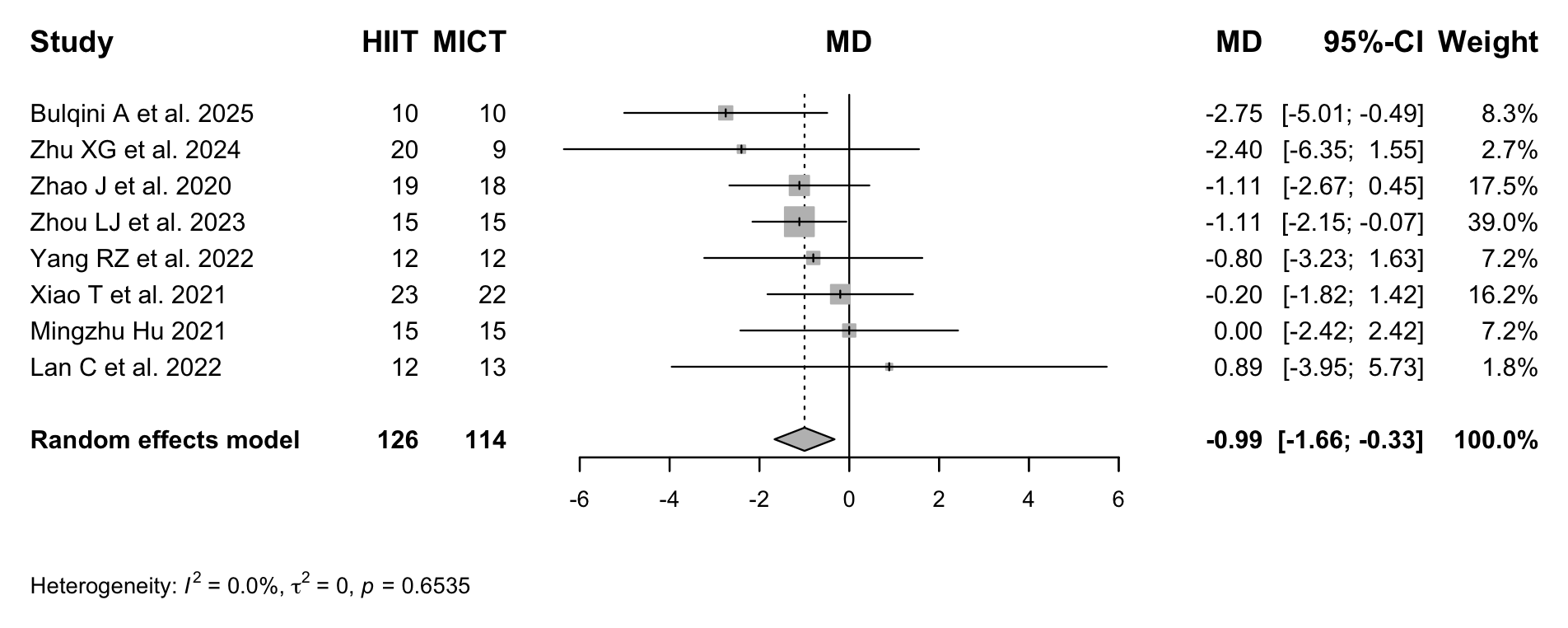


**Supplementary Figure S5. Forest plot for the effect of HIIT on muscle mass.**


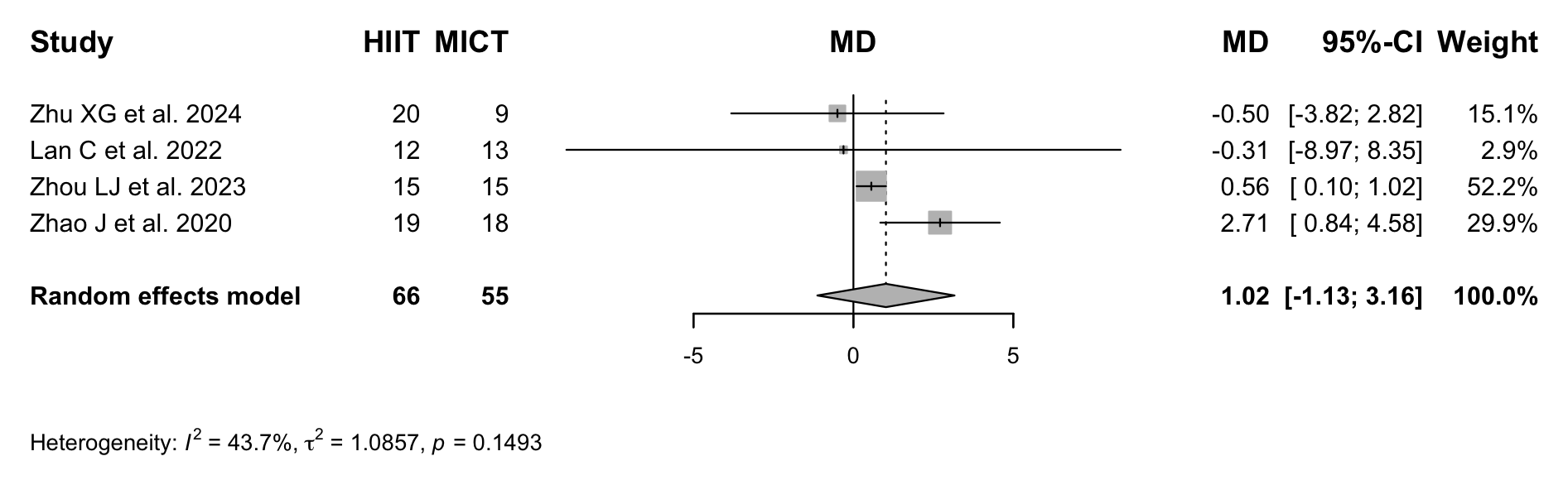


**Supplementary Figure S6. Forest plot for the effect of HIIT on waist circumference.**


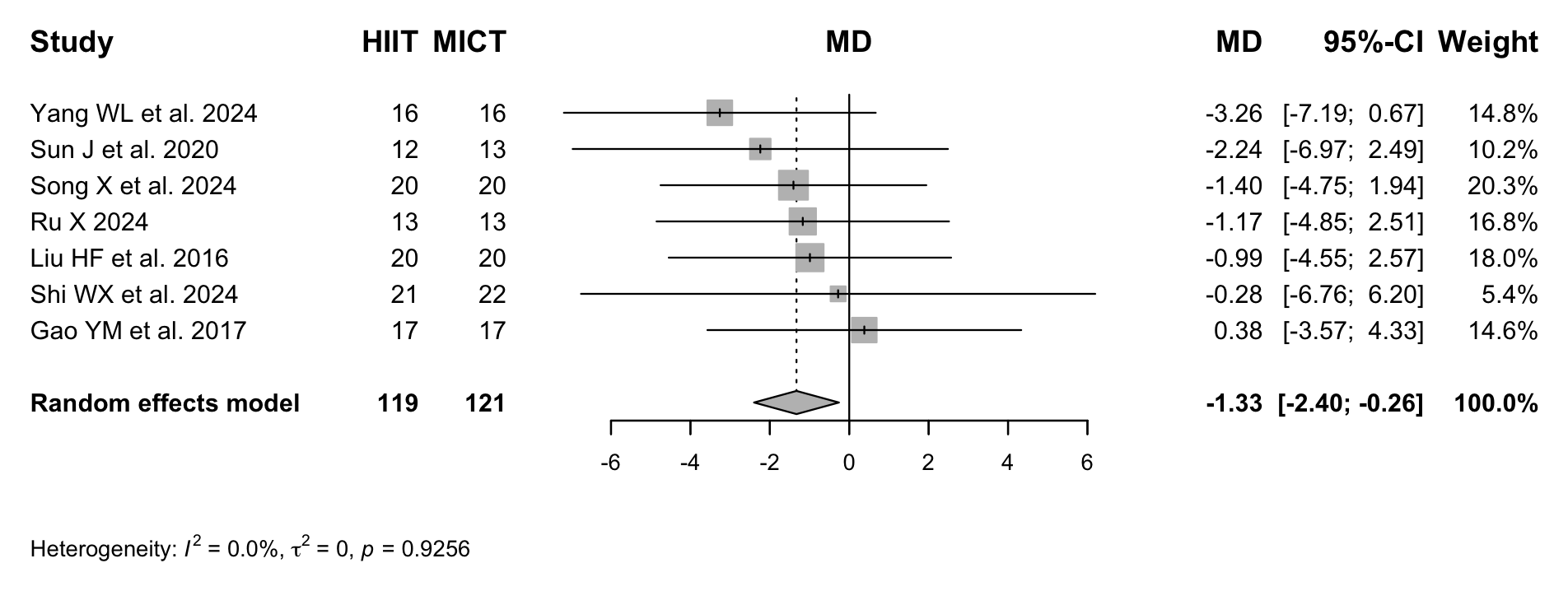


**Supplementary Figure S7. Forest plot for the effect of HIIT on hip circumference.**


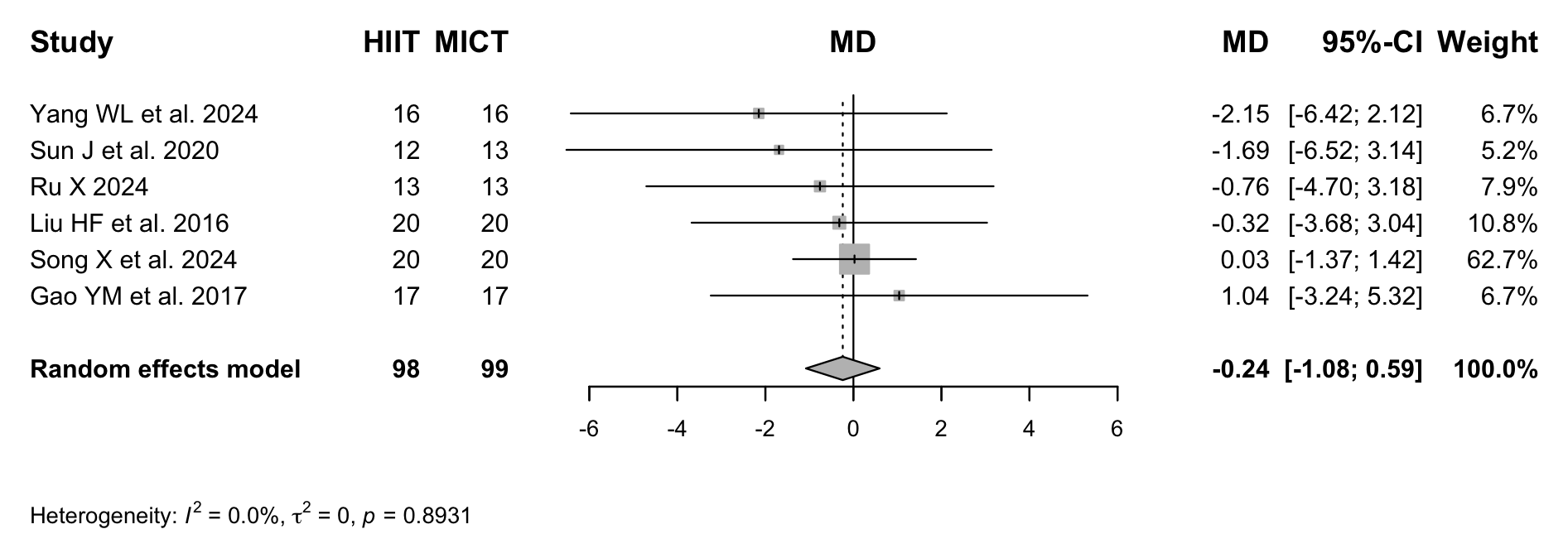


**Supplementary Figure S8. Forest plot for the effect of HIIT on waist-to-hip ratio (WHR).**


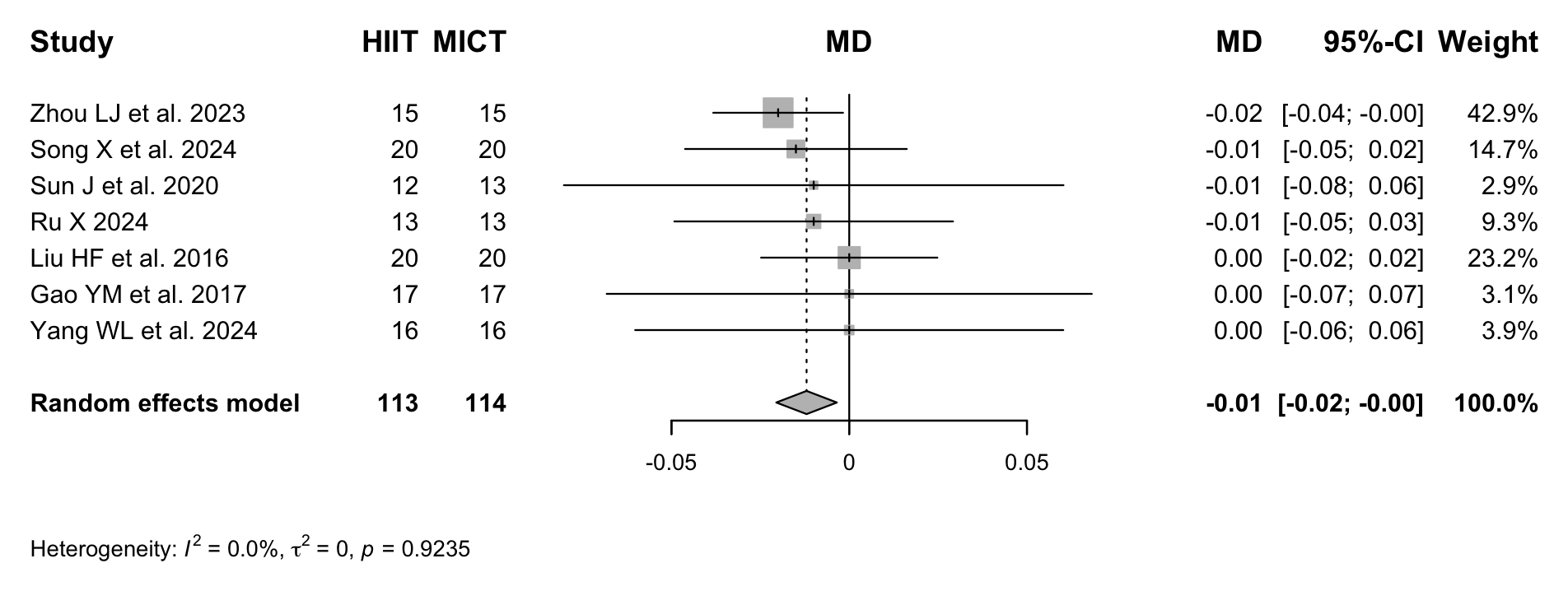


**Main analysis forest plots. Glucose and lipid metabolism-related indicators S9-S14.**

**Supplementary Figure S9. Forest plot for the effect of HIIT on fasting blood glucose (FBG).**


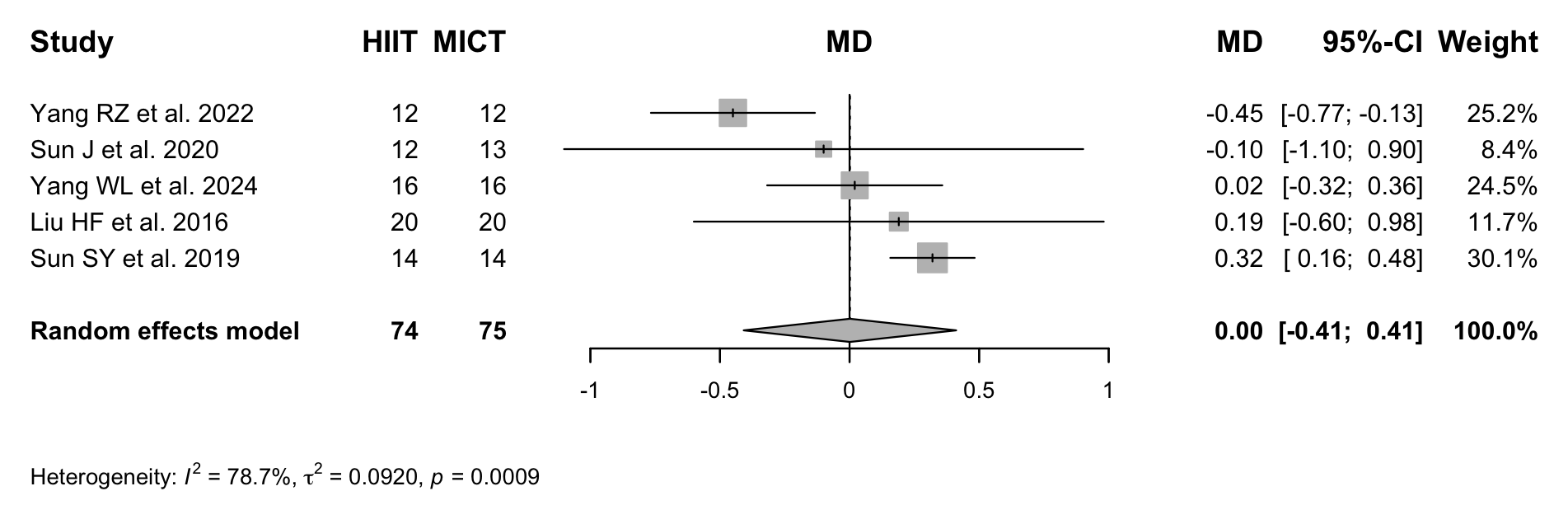


**Supplementary Figure S10. Forest plot for the effect of HIIT on fasting insulin (FINS).**


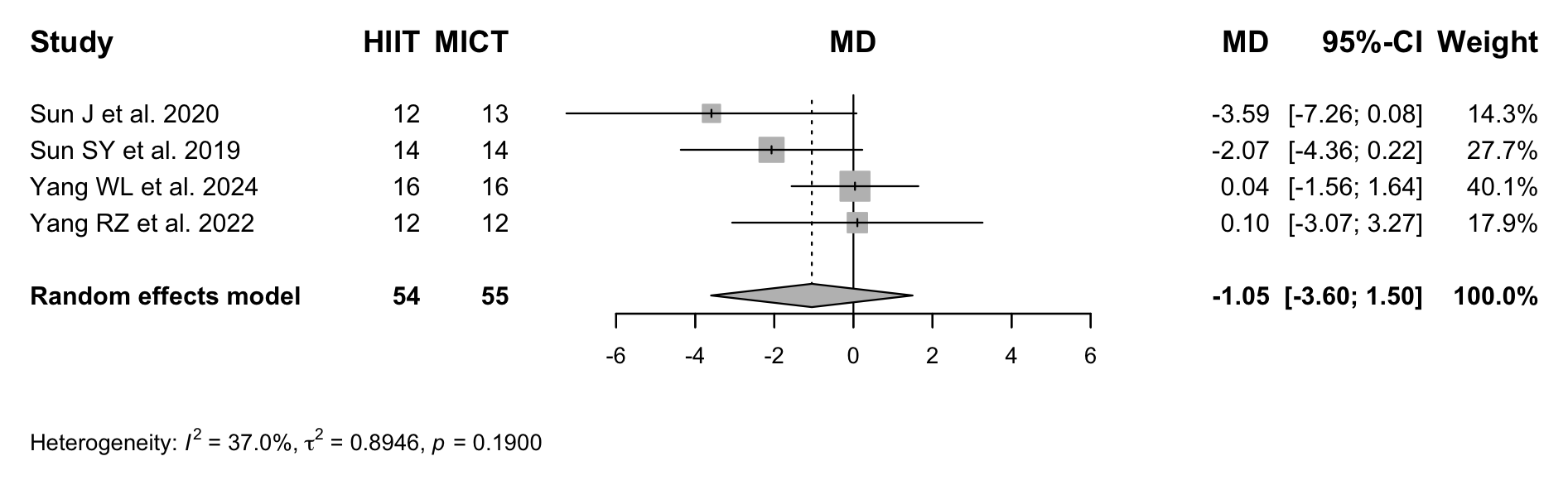


**Supplementary Figure S11. Forest plot for the effect of HIIT on total cholesterol (TC).**


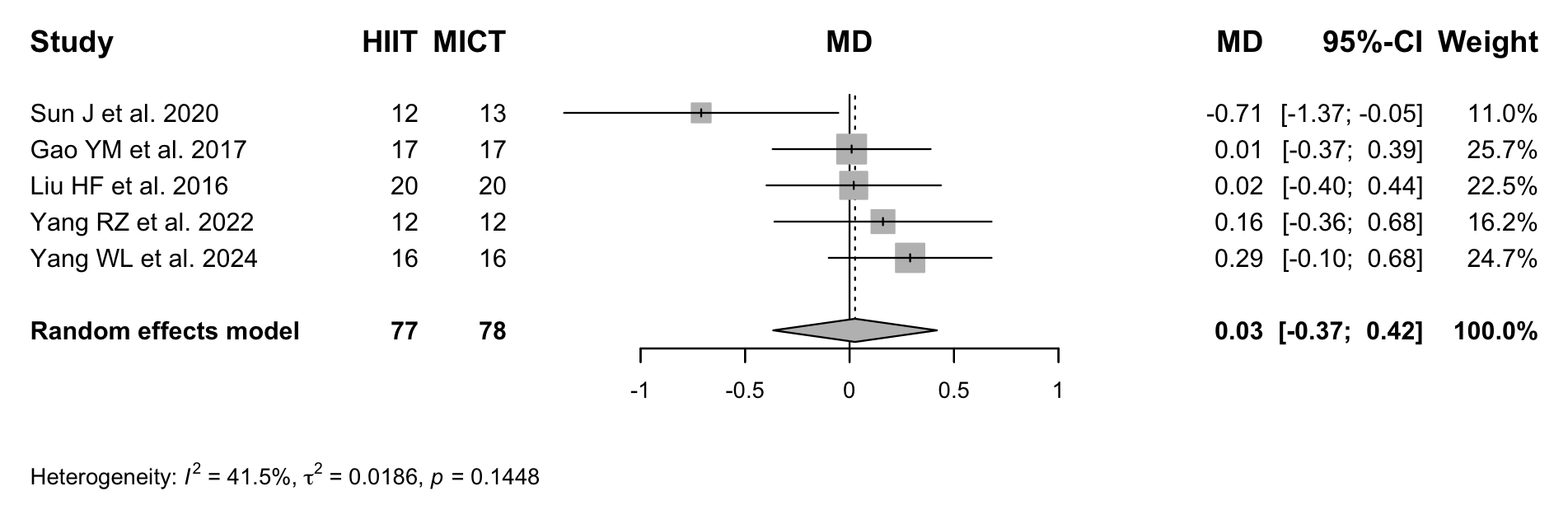


**Supplementary Figure S12. Forest plot for the effect of HIIT on high-density lipoprotein cholesterol (HDL-C).**


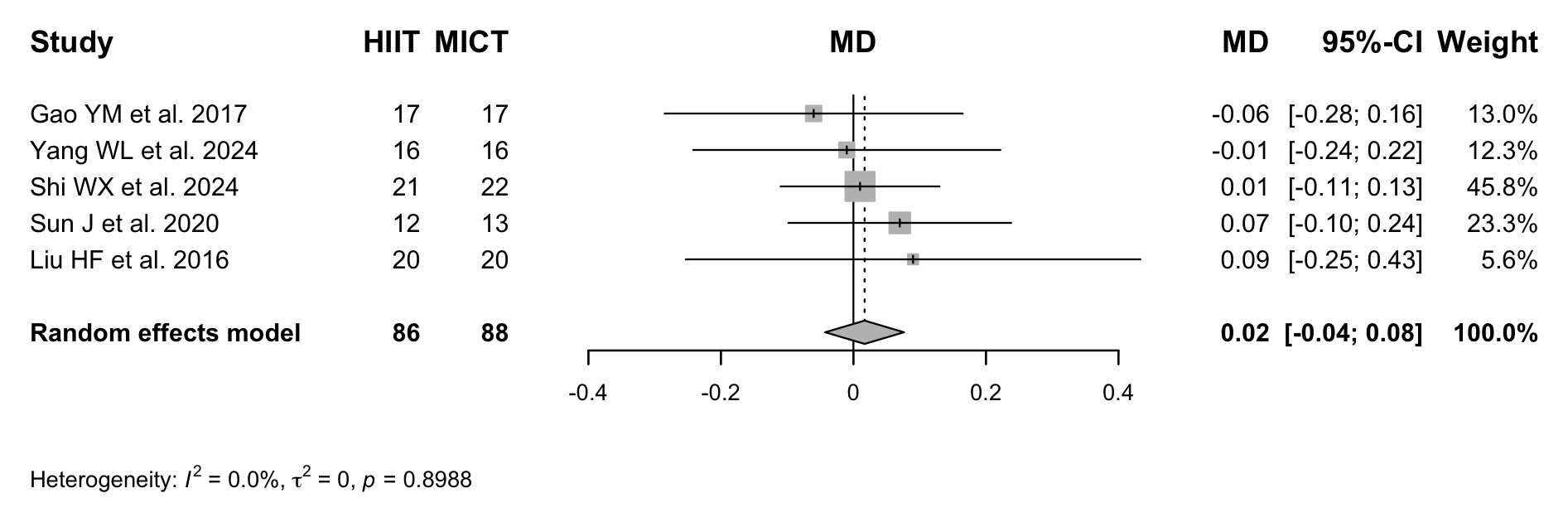


**Supplementary Figure S13. Forest plot for the effect of HIIT on low-density lipoprotein cholesterol (LDL-C).**


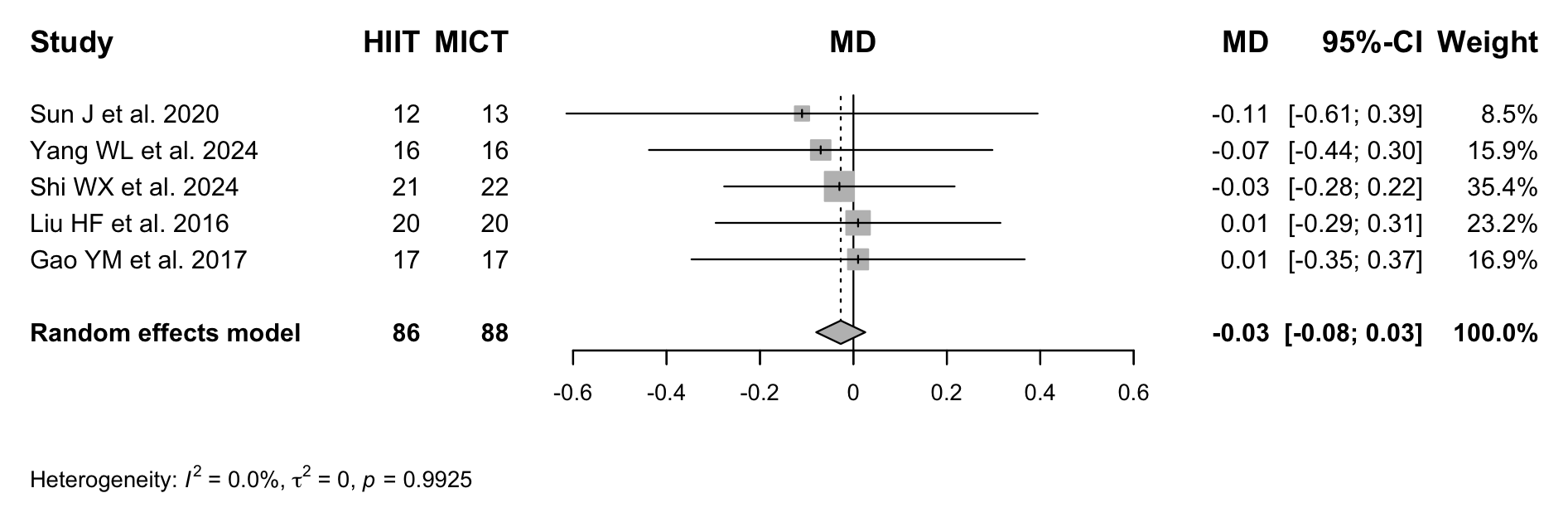


**Supplementary Figure S14. Forest plot for the effect of HIIT on triglycerides (TG).**


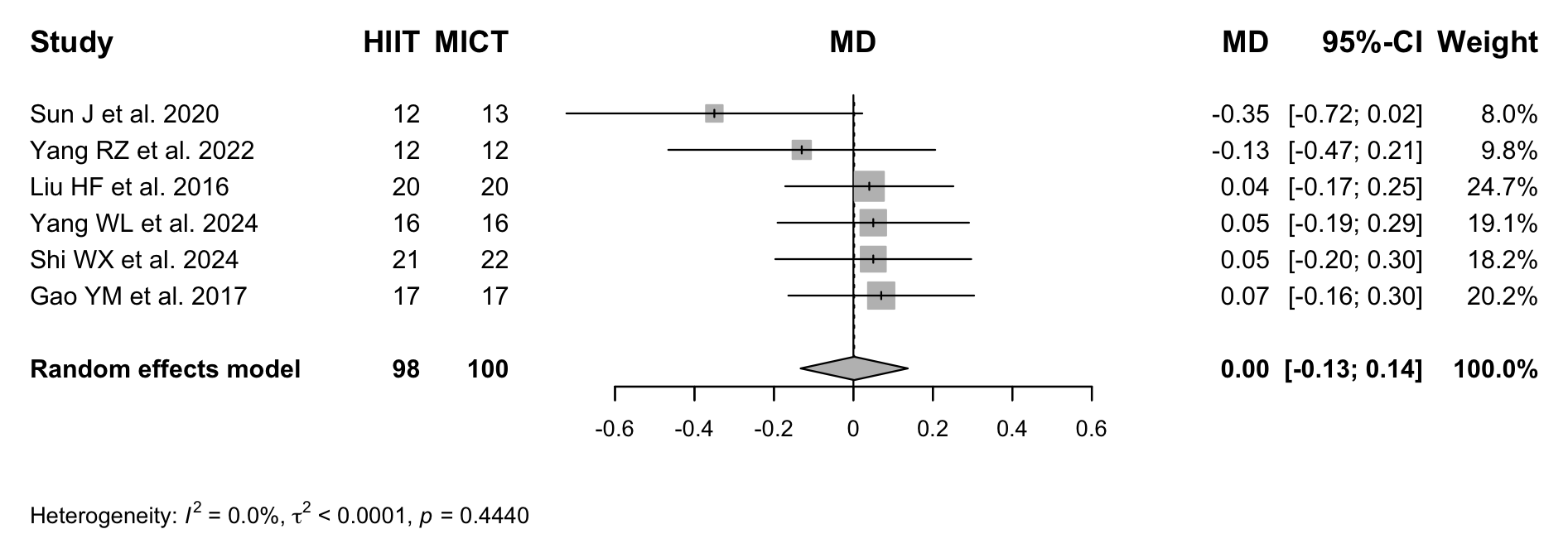


**Subgroup analyses: body morphology outcomes (S15-S36)**

**Supplementary Figure S15. Subgroup analysis of the effect of HIIT on body weight by sex.**


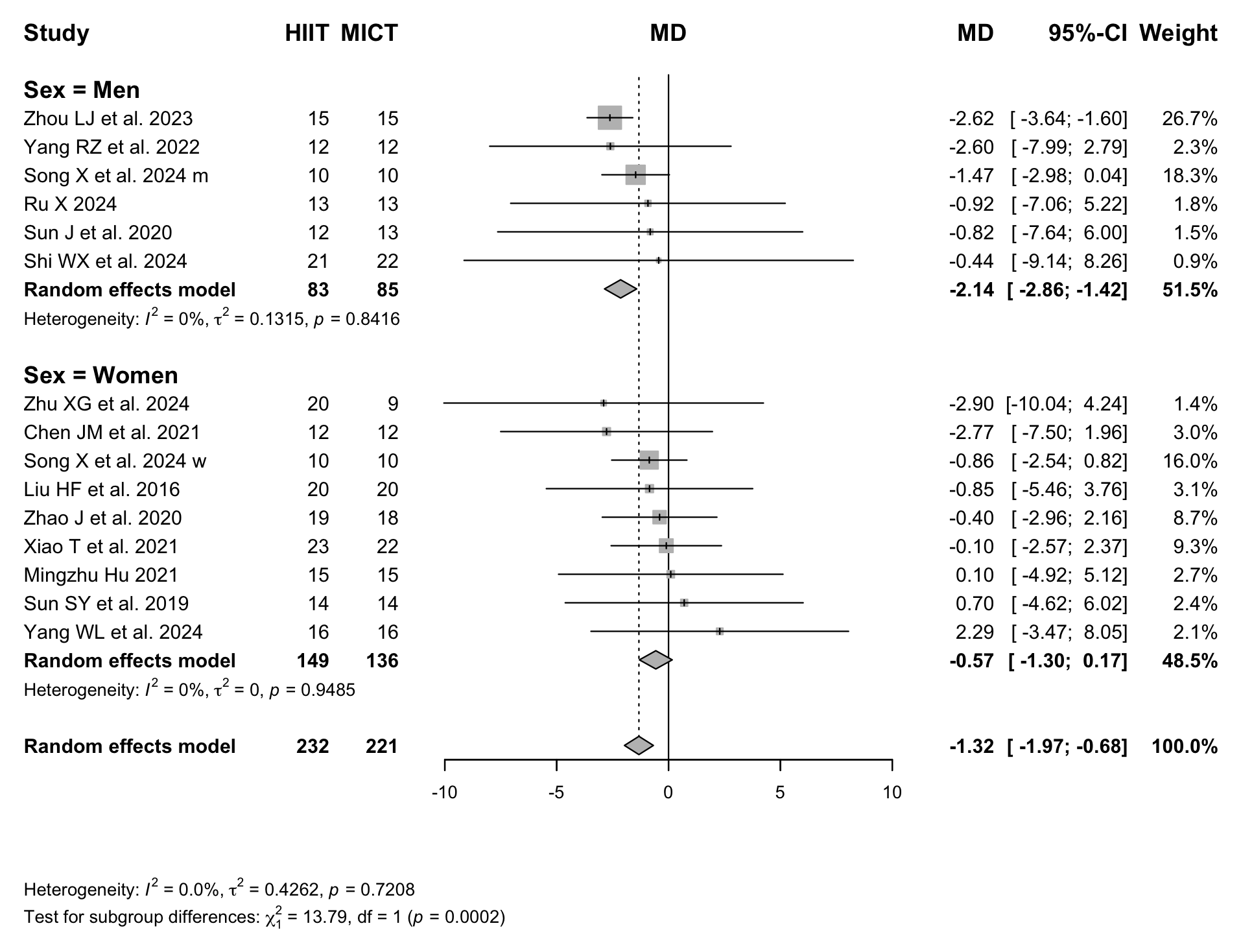


**Supplementary Figure S16. Subgroup analysis of the effect of HIIT on body weight by weight status.**


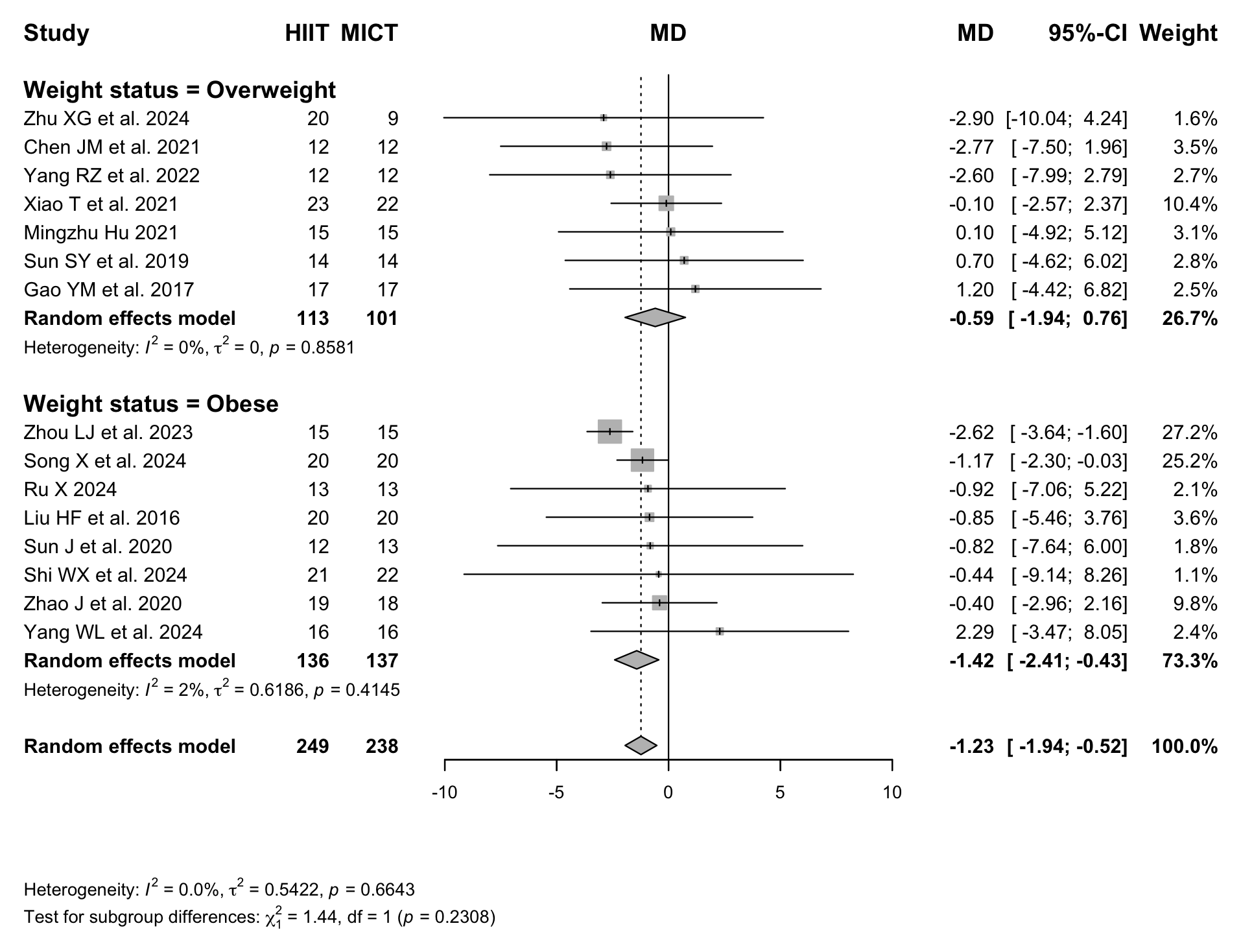


**Supplementary Figure S17. Subgroup analysis of the effect of HIIT on body weight by intervention duration.**


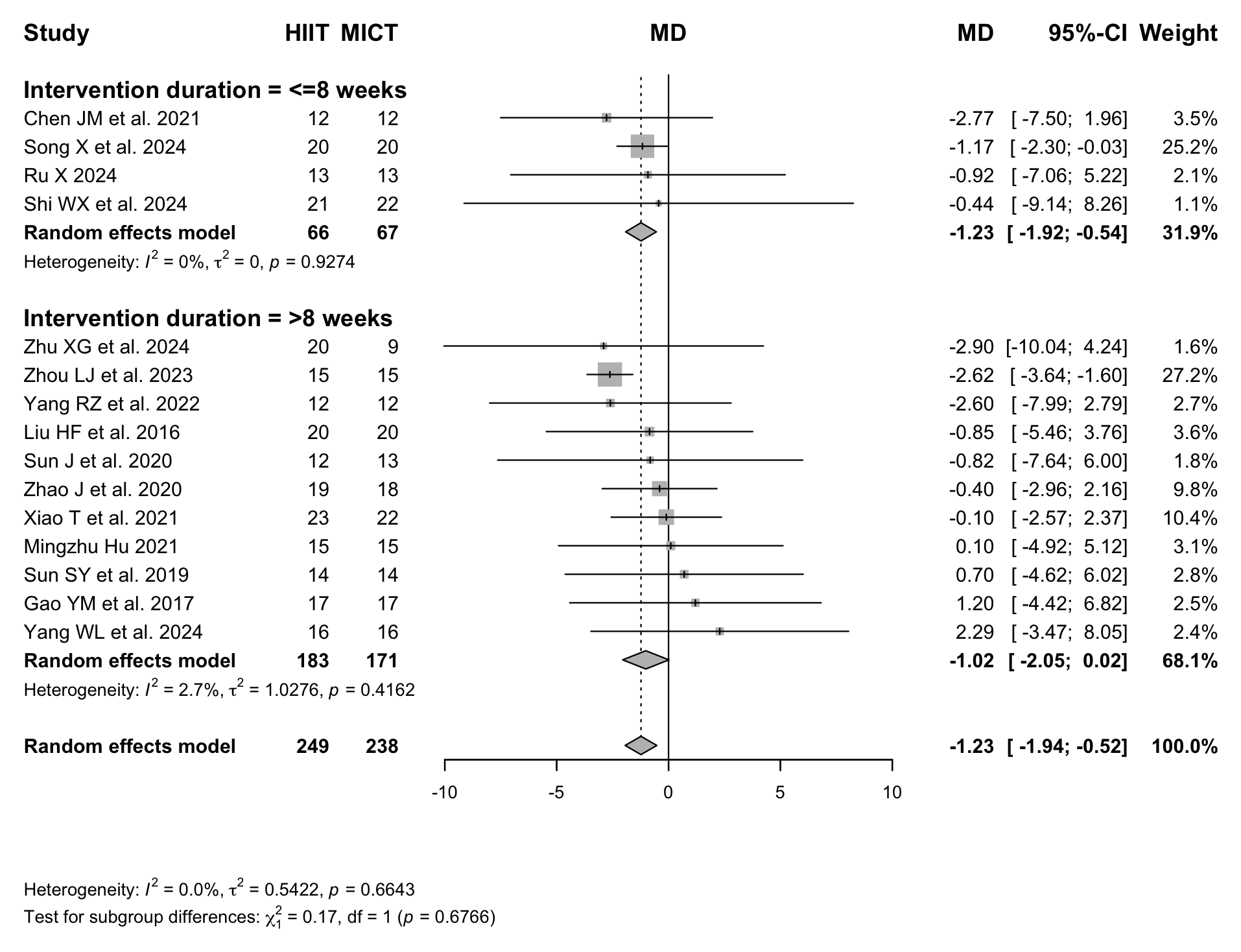


**Supplementary Figure S18. Subgroup analysis of the effect of HIIT on body weight by training frequency.**


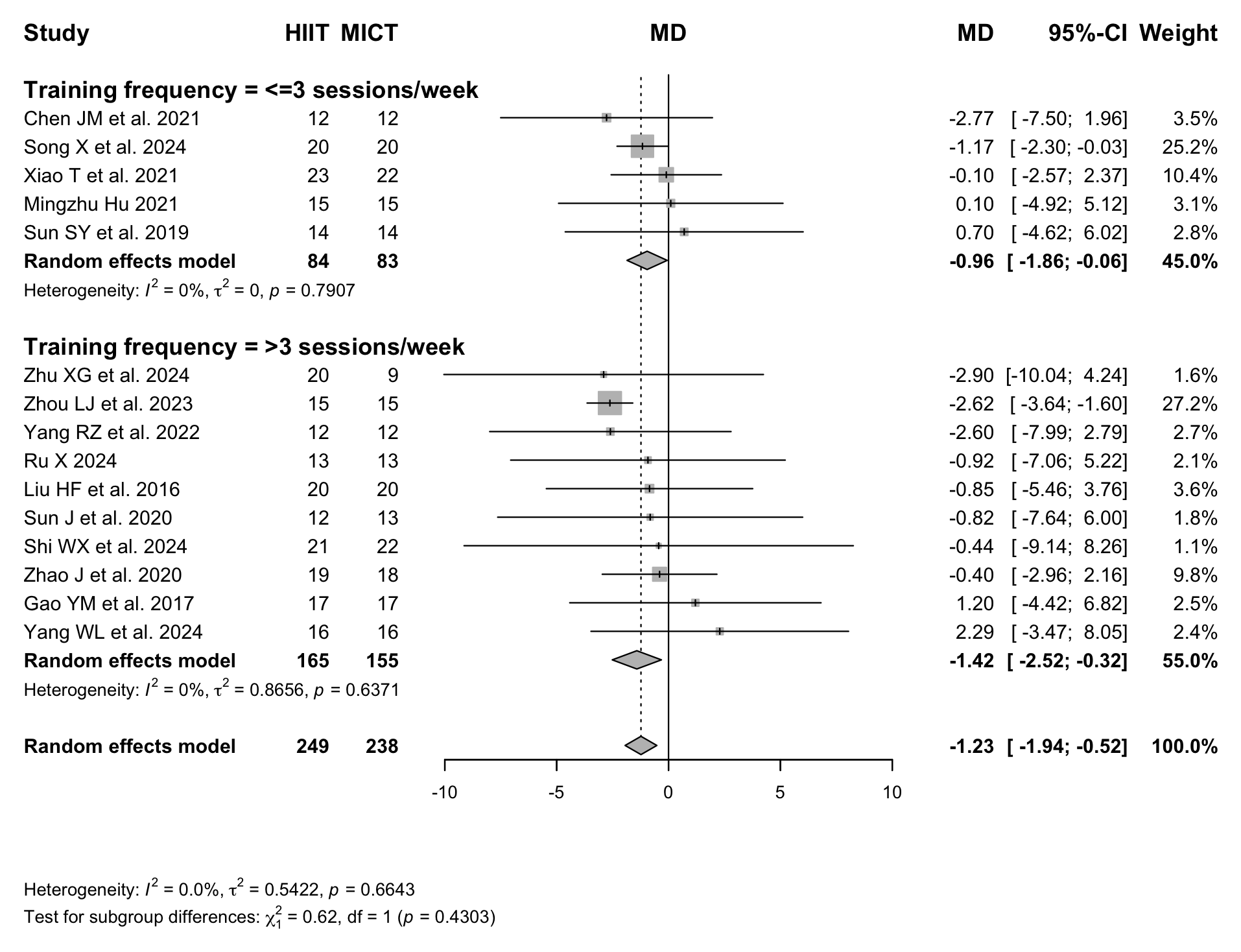


**Supplementary Figure S19. Subgroup analysis of the effect of HIIT on body mass index (BMI) by sex.**


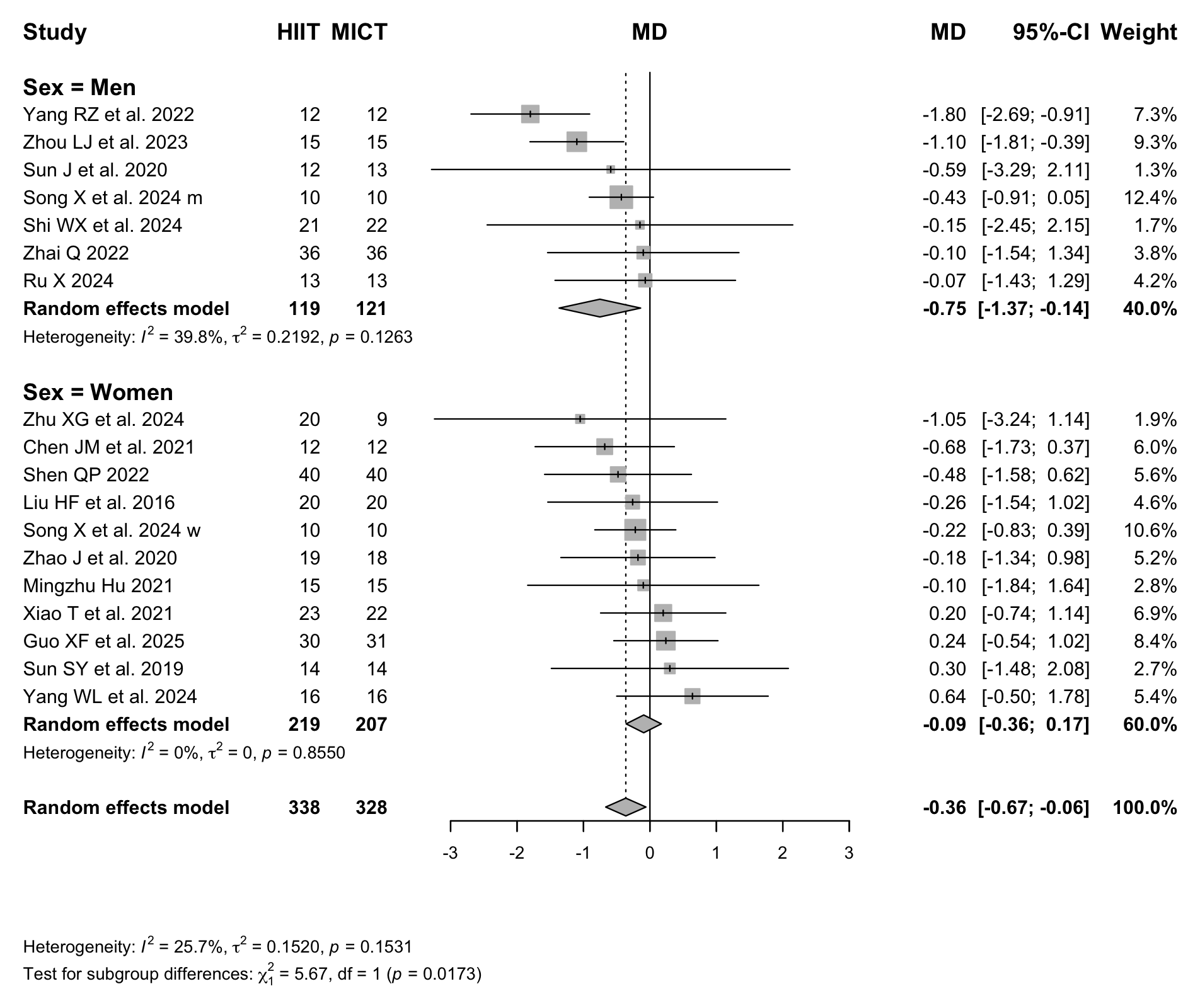


**Supplementary Figure S20. Subgroup analysis of the effect of HIIT on body mass index (BMI) by weight status.**


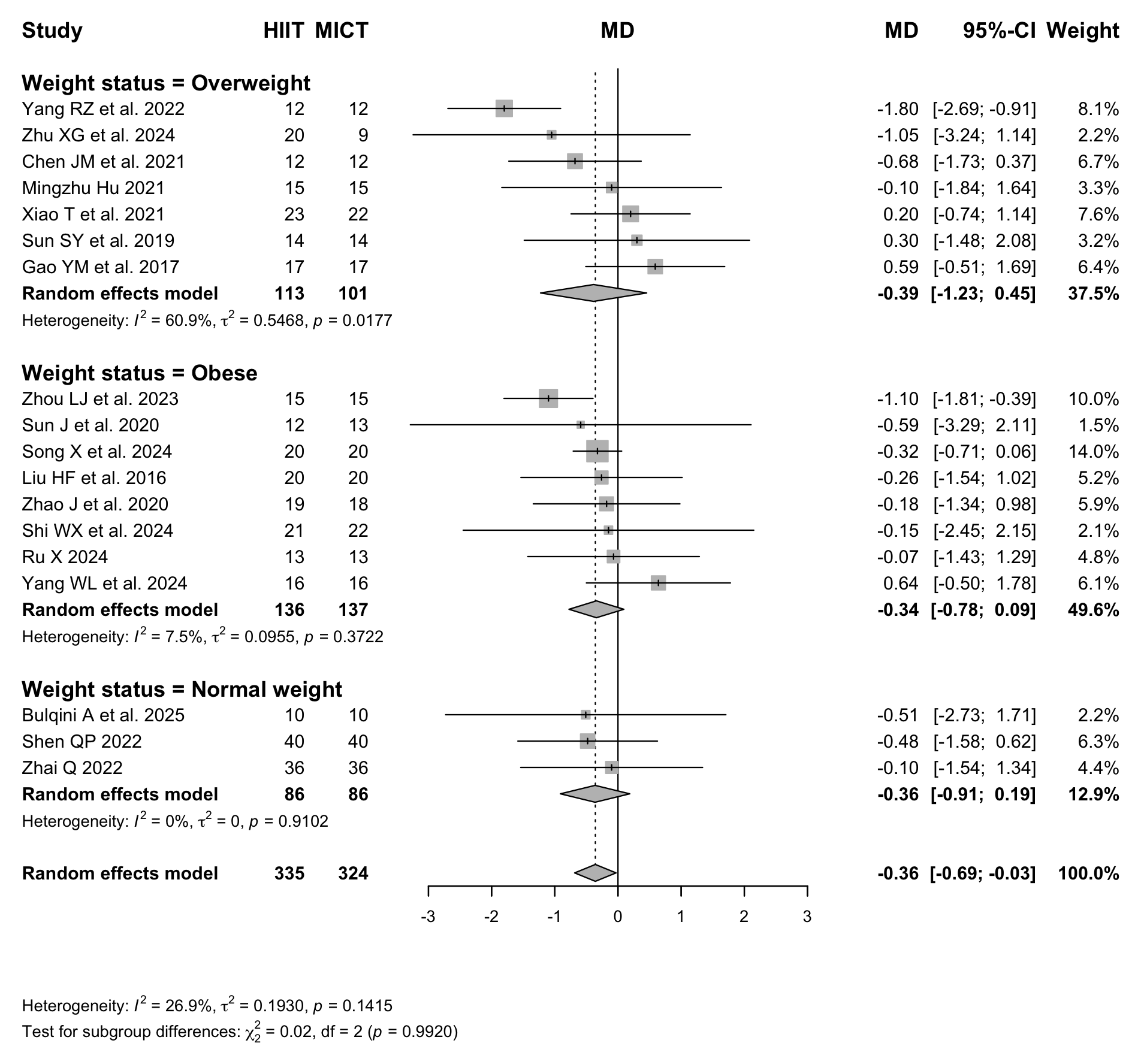


**Supplementary Figure S21. Subgroup analysis of the effect of HIIT on body mass index (BMI) by intervention duration.**


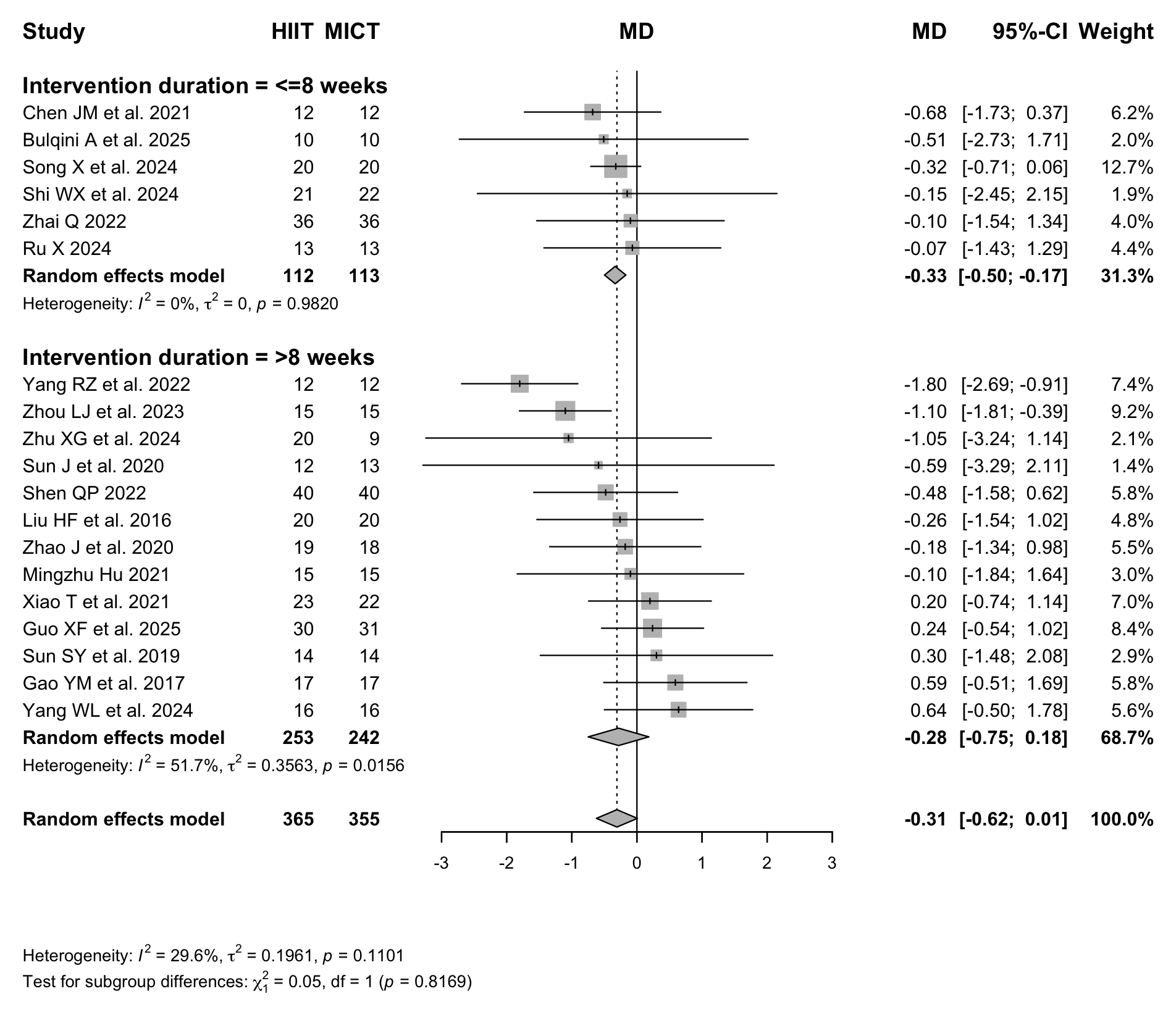


**Supplementary Figure S22. Subgroup analysis of the effect of HIIT on body mass index (BMI) by training frequency.**


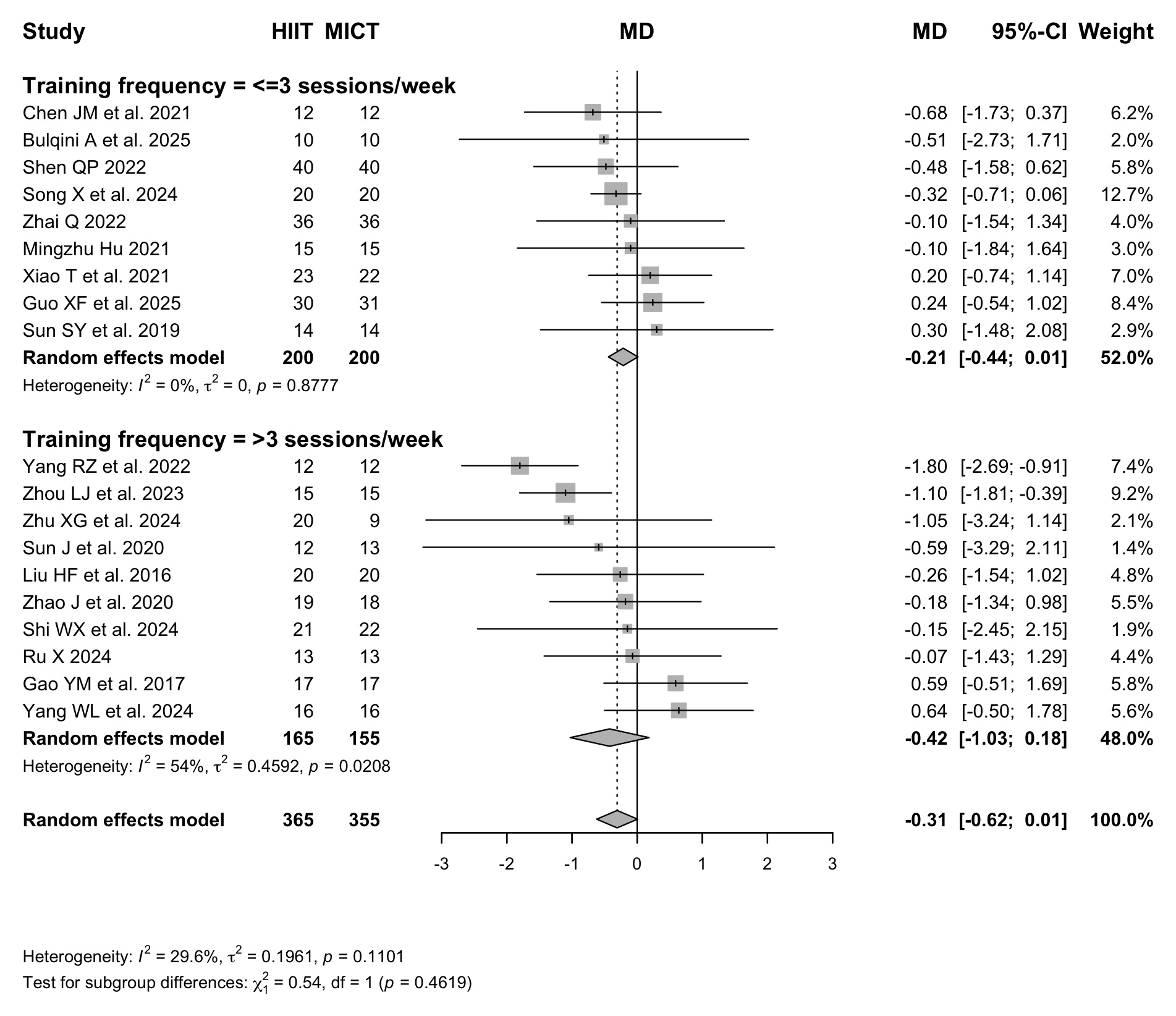


**Supplementary Figure S23. Subgroup analysis of the effect of HIIT on body fat percentage by sex.**


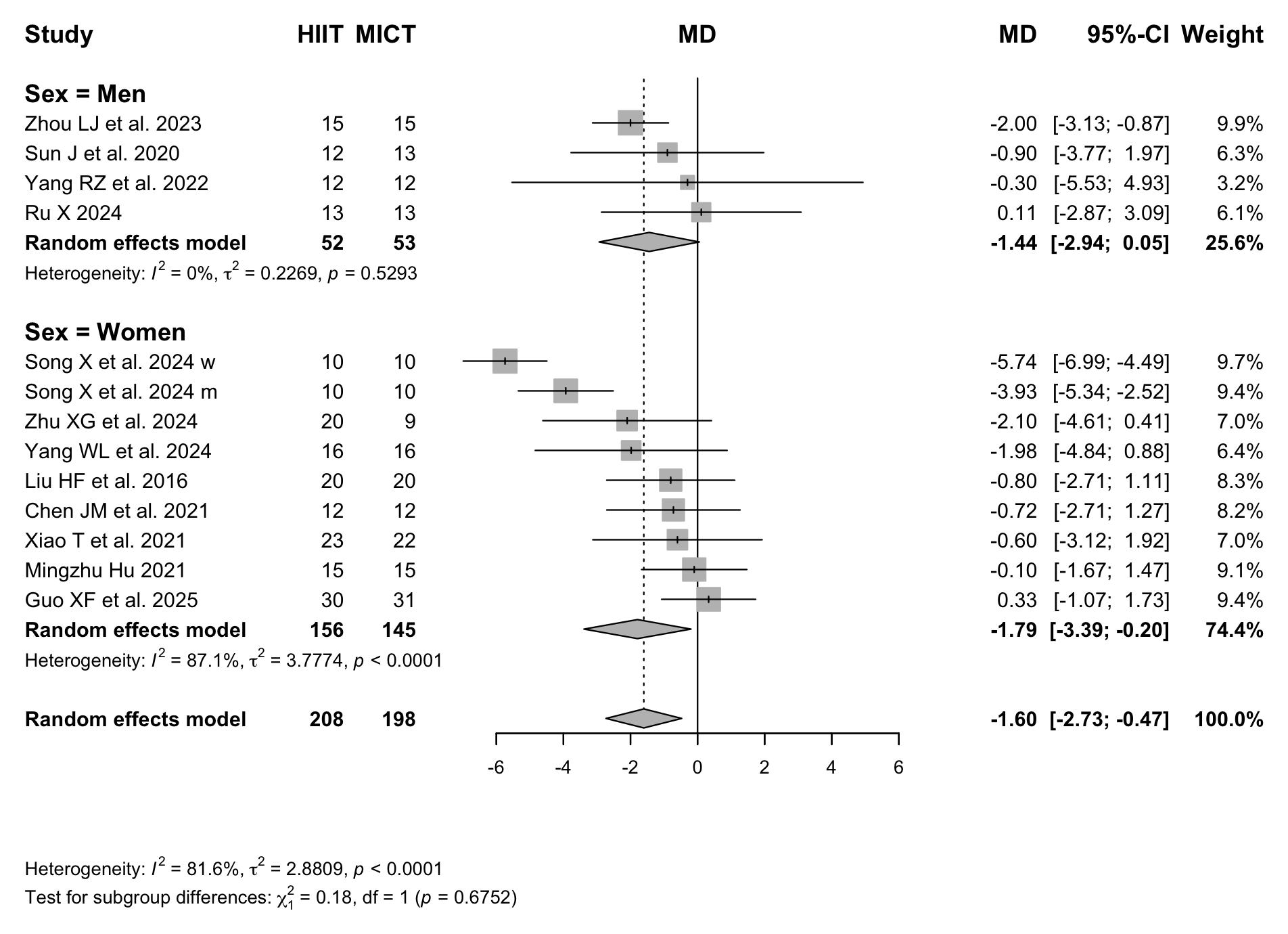


**Supplementary Figure S24. Subgroup analysis of the effect of HIIT on body fat percentage by weight status.**


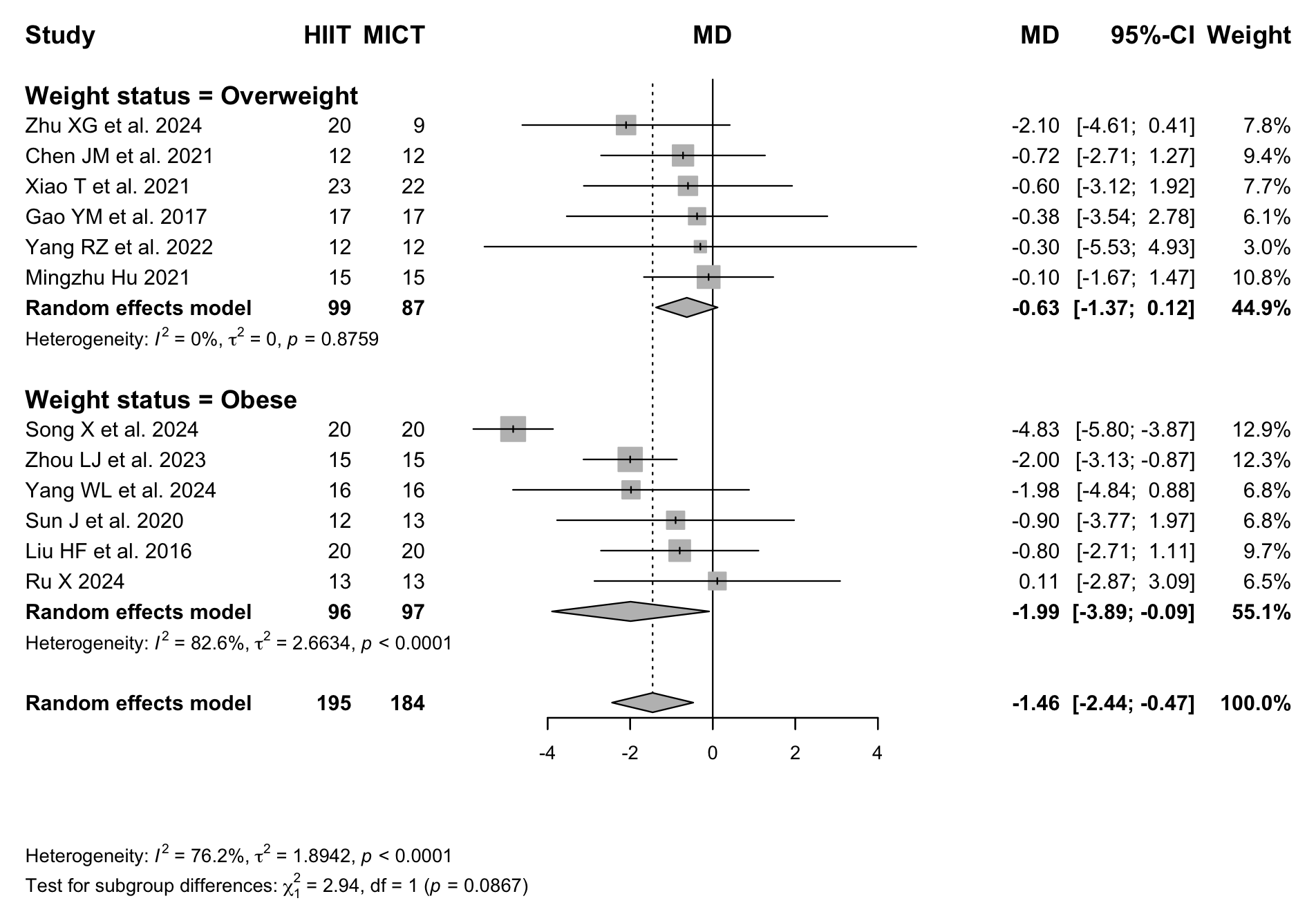


**Supplementary Figure S25. Subgroup analysis of the effect of HIIT on body fat percentage by intervention duration.**


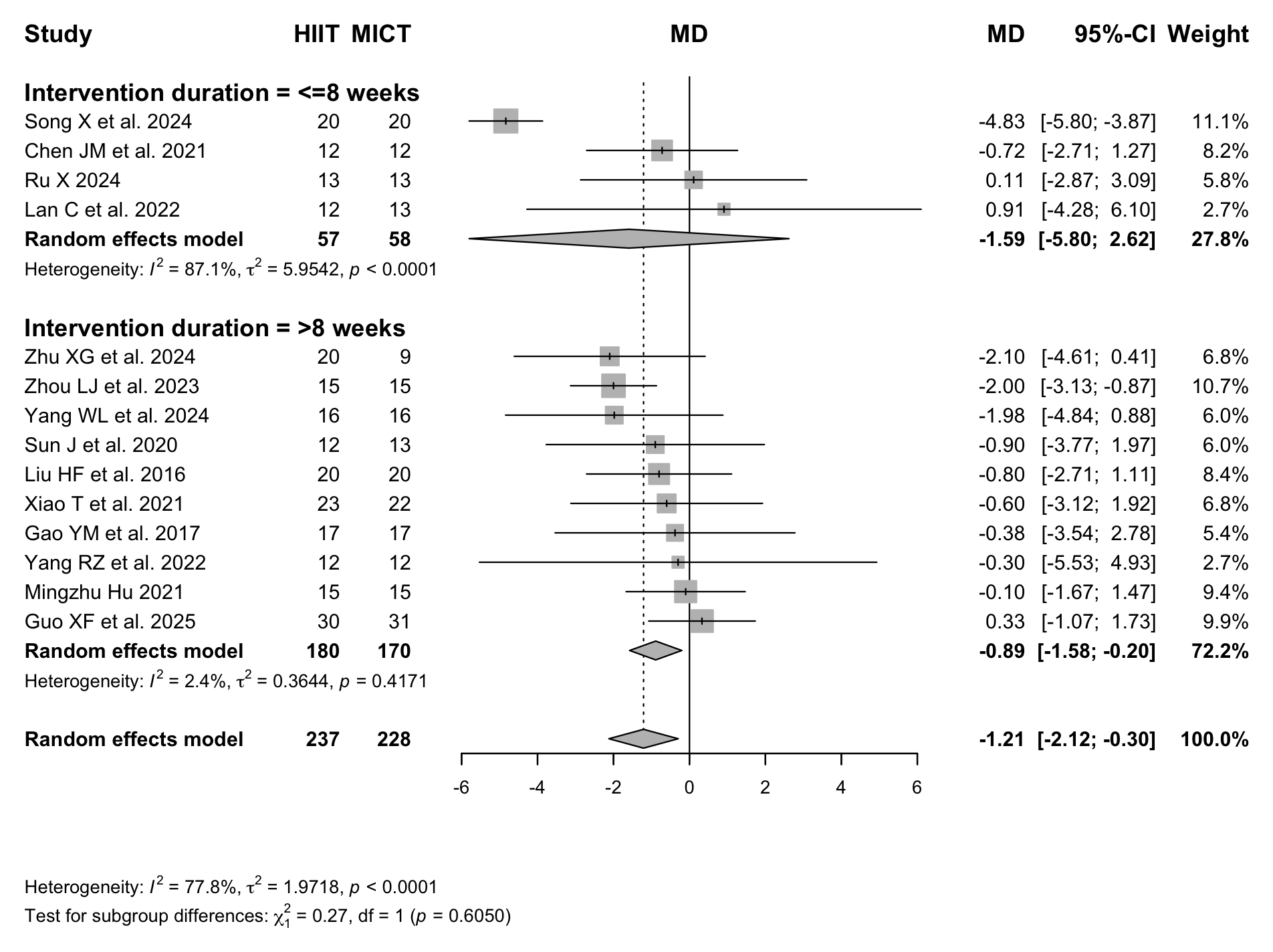


**Supplementary Figure S26. Subgroup analysis of the effect of HIIT on body fat percentage by training frequency.**


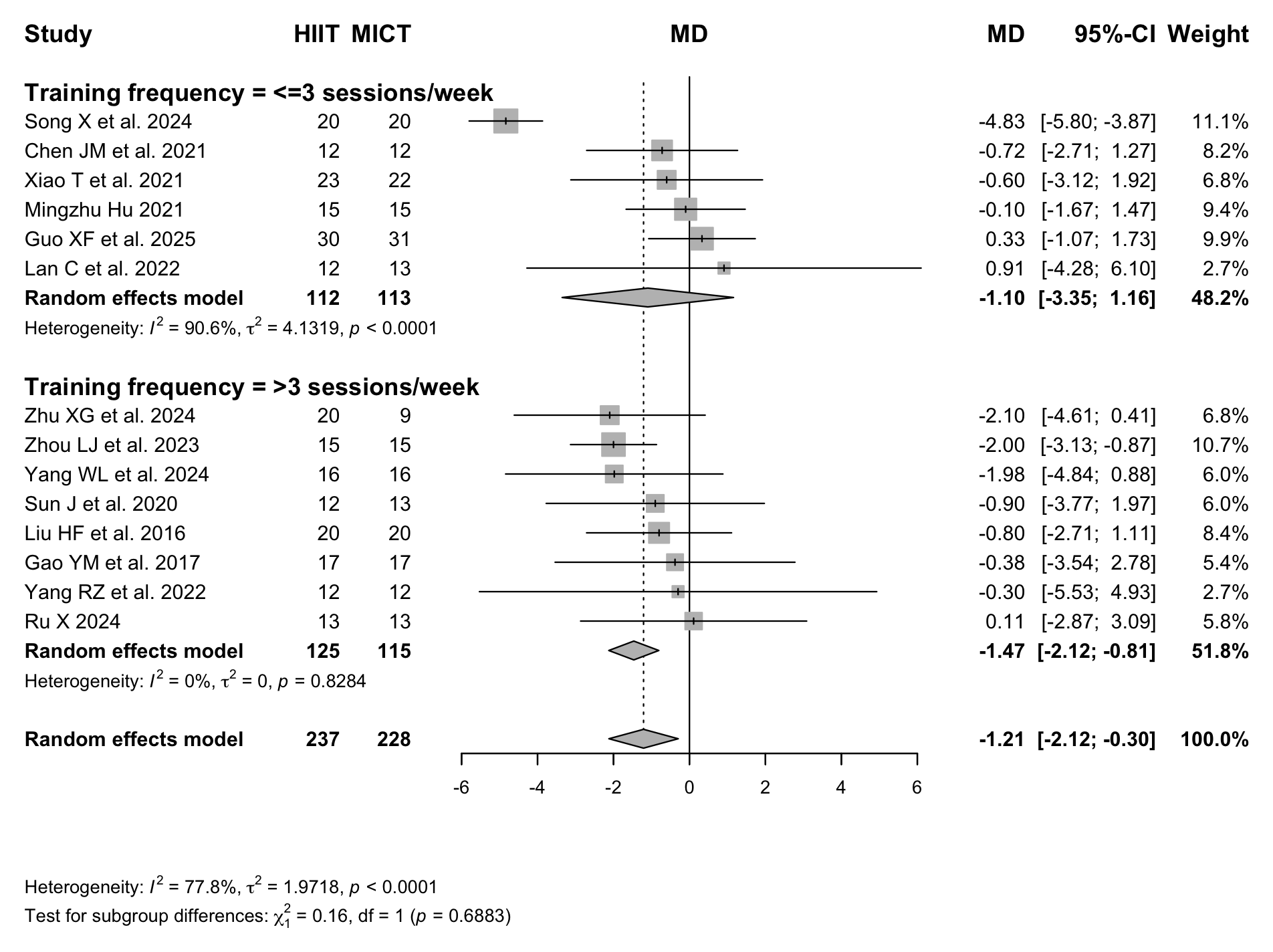


**Supplementary Figure S27. Subgroup analysis of the effect of HIIT on fat mass by sex.**


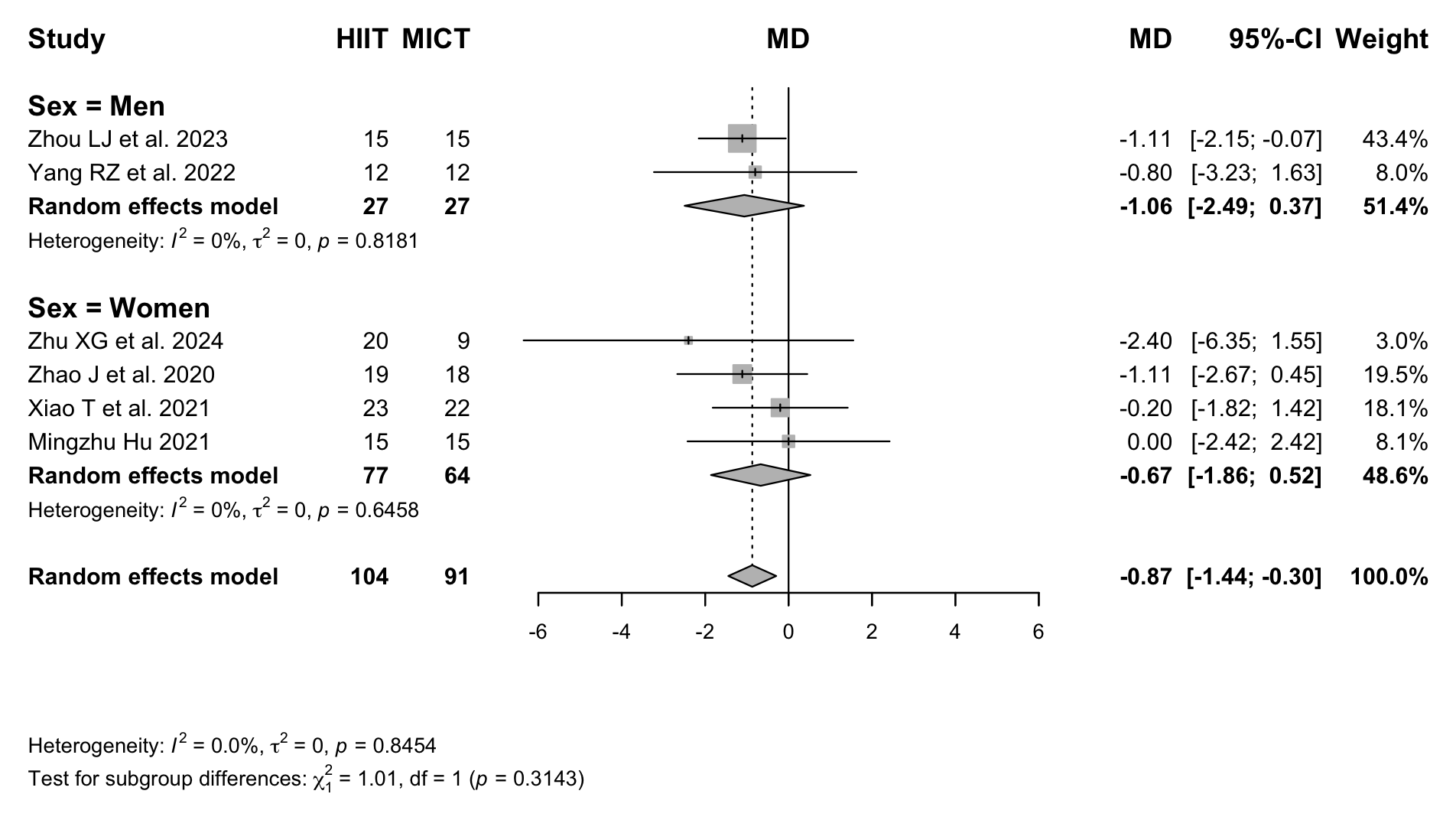


**Supplementary Figure S28. Subgroup analysis of the effect of HIIT on fat mass by weight status.**


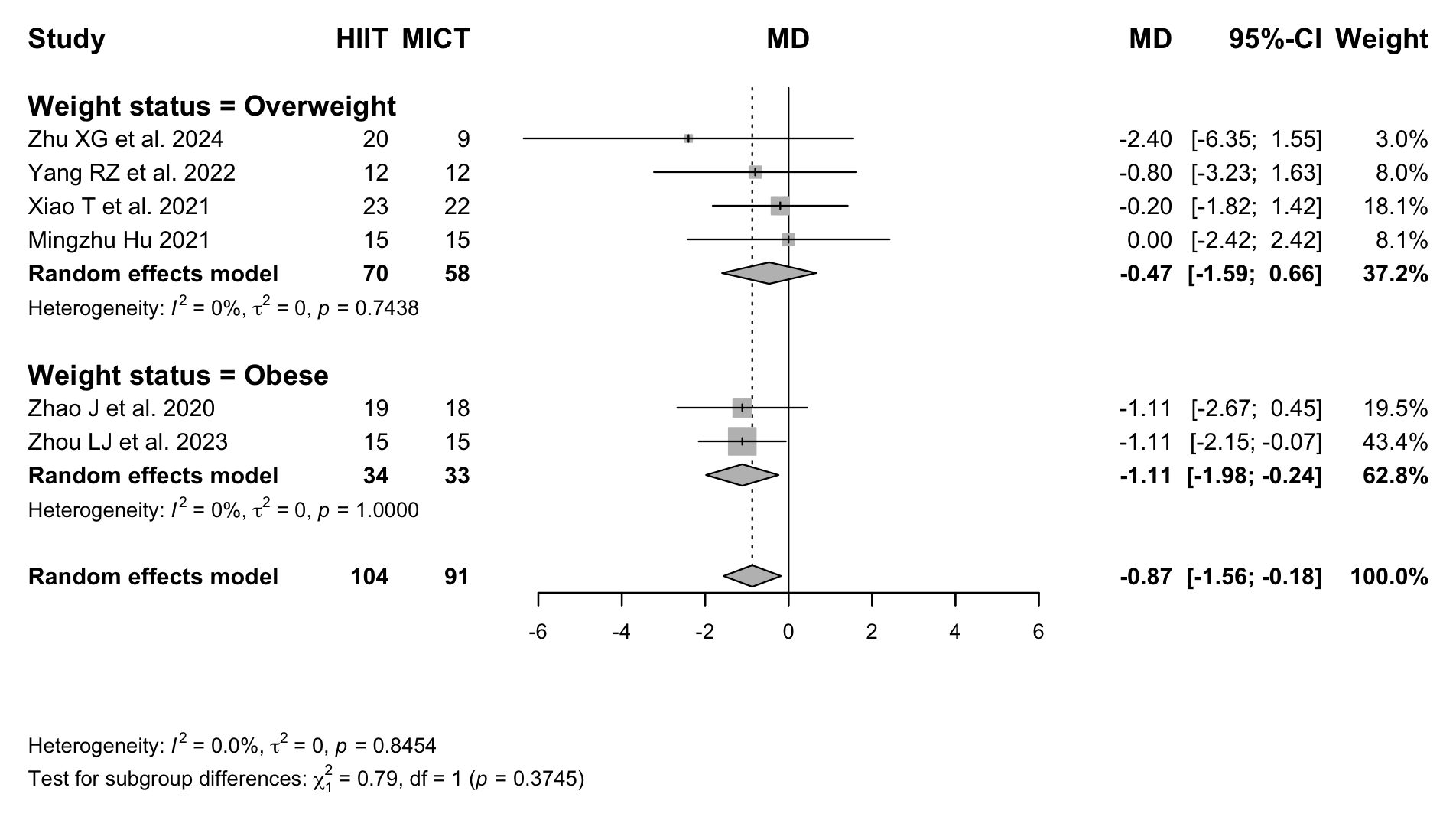


**Supplementary Figure S29. Subgroup analysis of the effect of HIIT on fat mass by intervention duration.**


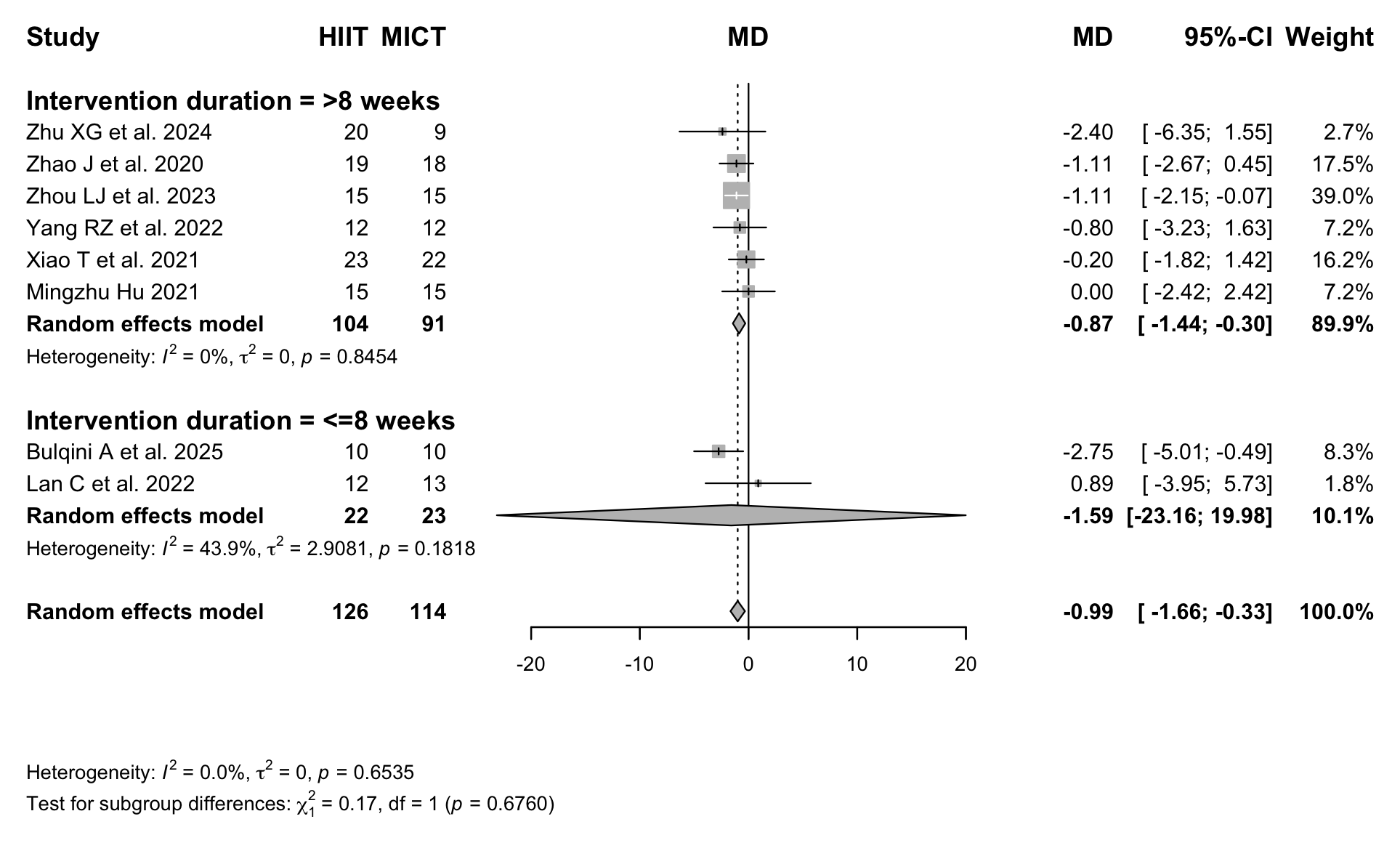


**Supplementary Figure S30. Subgroup analysis of the effect of HIIT on fat mass by training frequency.**


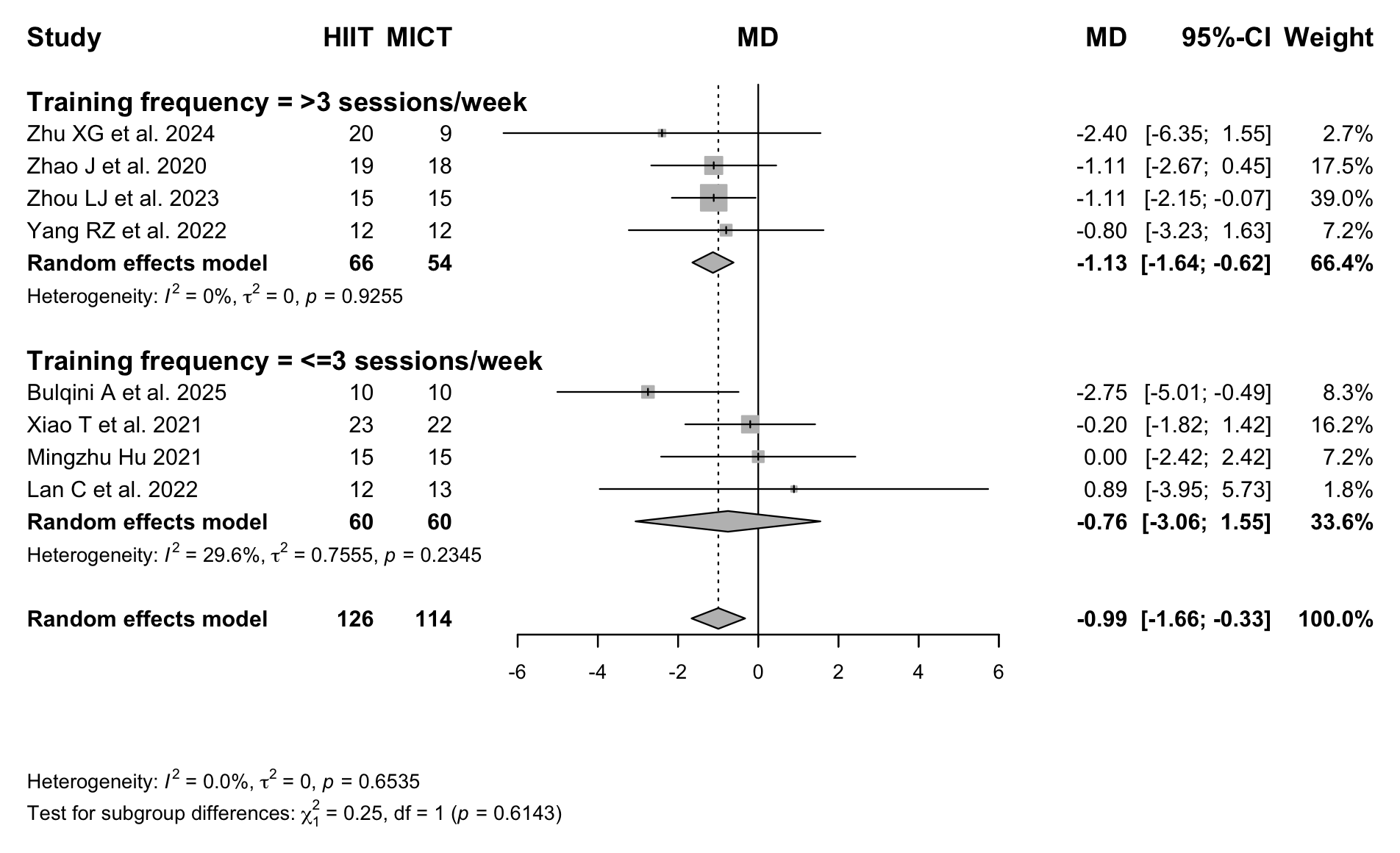


**Supplementary Figure S31. Subgroup analysis of the effect of HIIT on waist circumference by sex.**


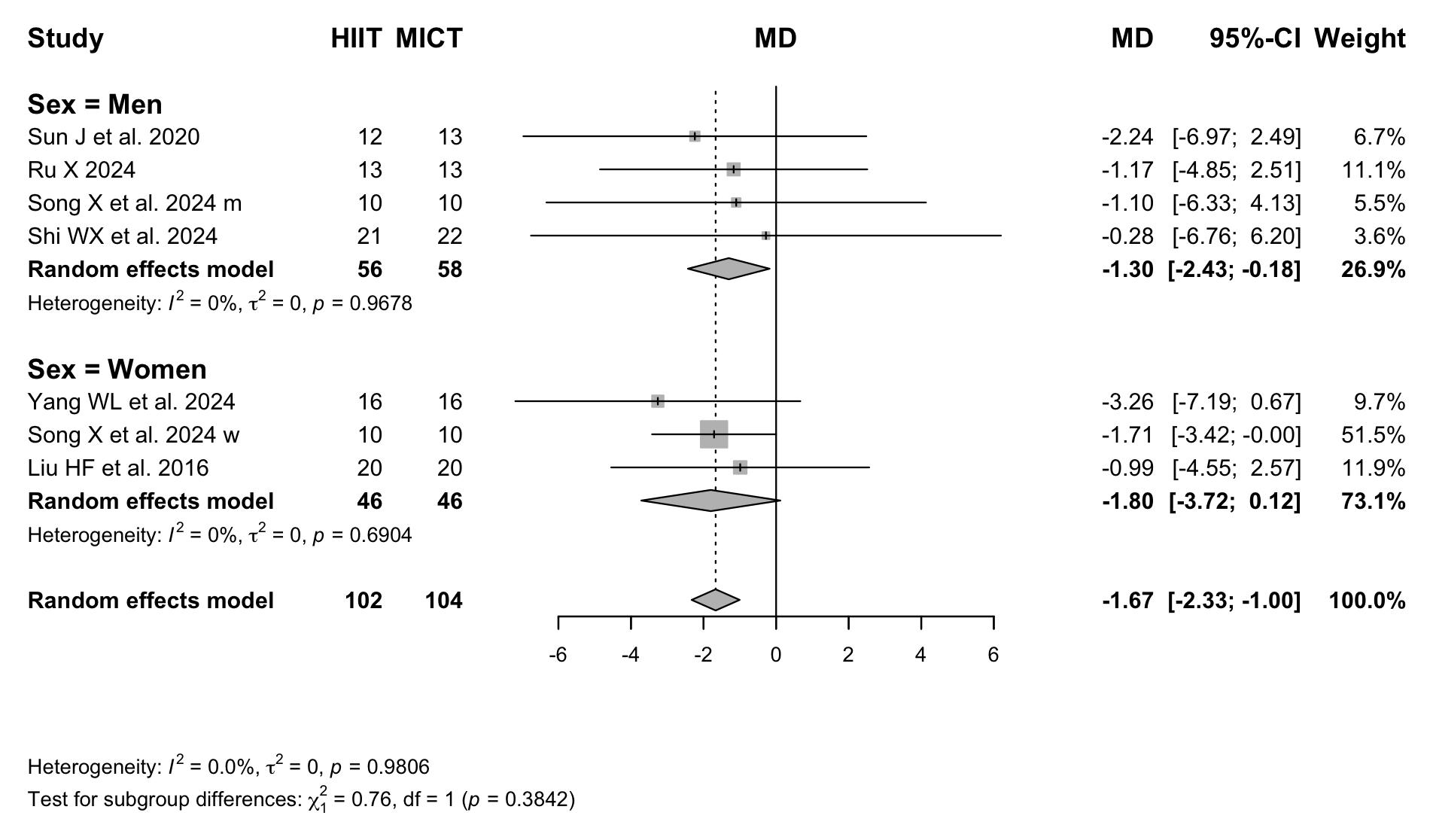


**Supplementary Figure S32. Subgroup analysis of the effect of HIIT on waist circumference by intervention duration.**


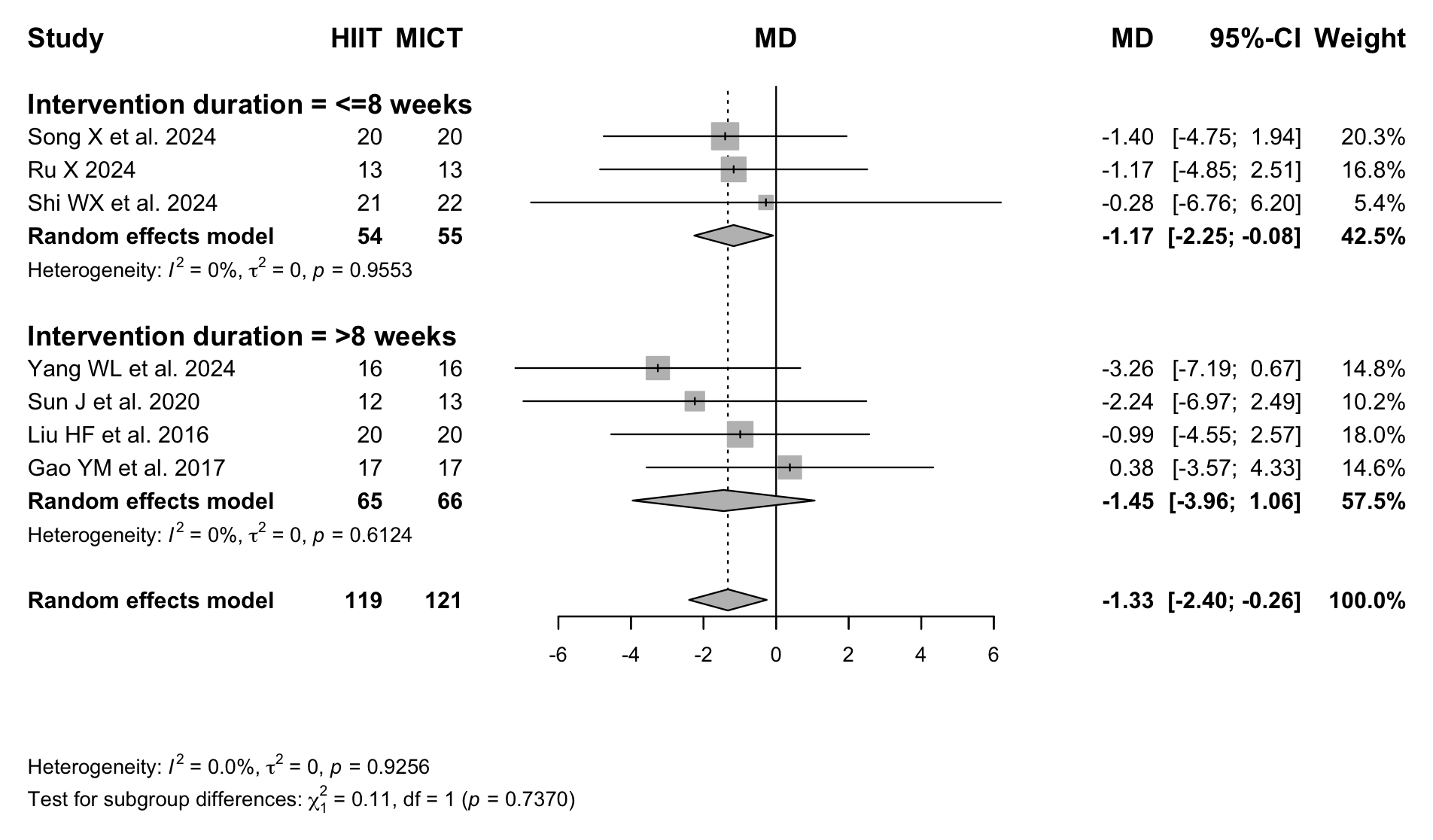


**Supplementary Figure S33. Subgroup analysis of the effect of HIIT on hip circumference by sex.**


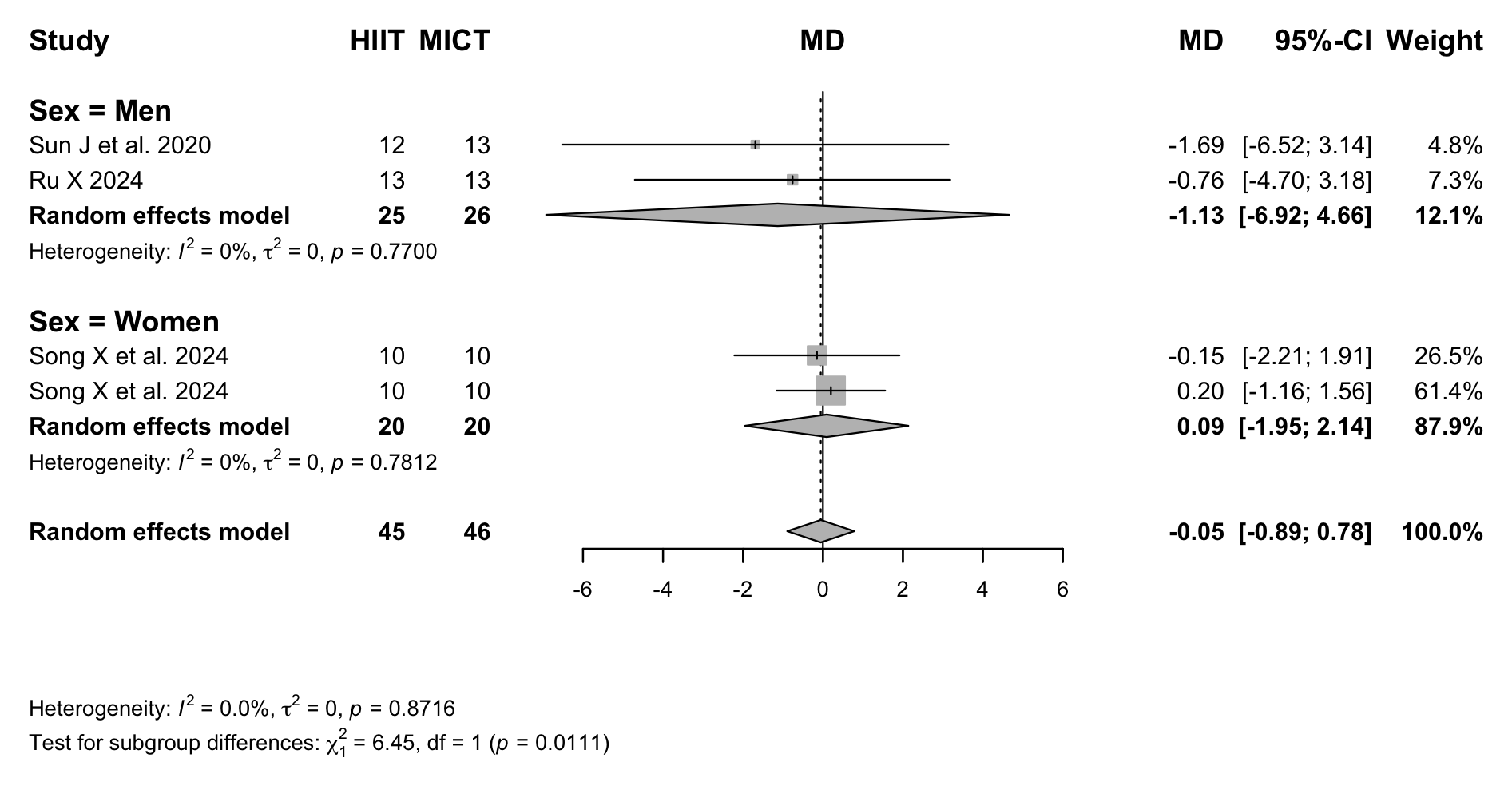


**Supplementary Figure S34. Subgroup analysis of the effect of HIIT on hip circumference by intervention duration.**


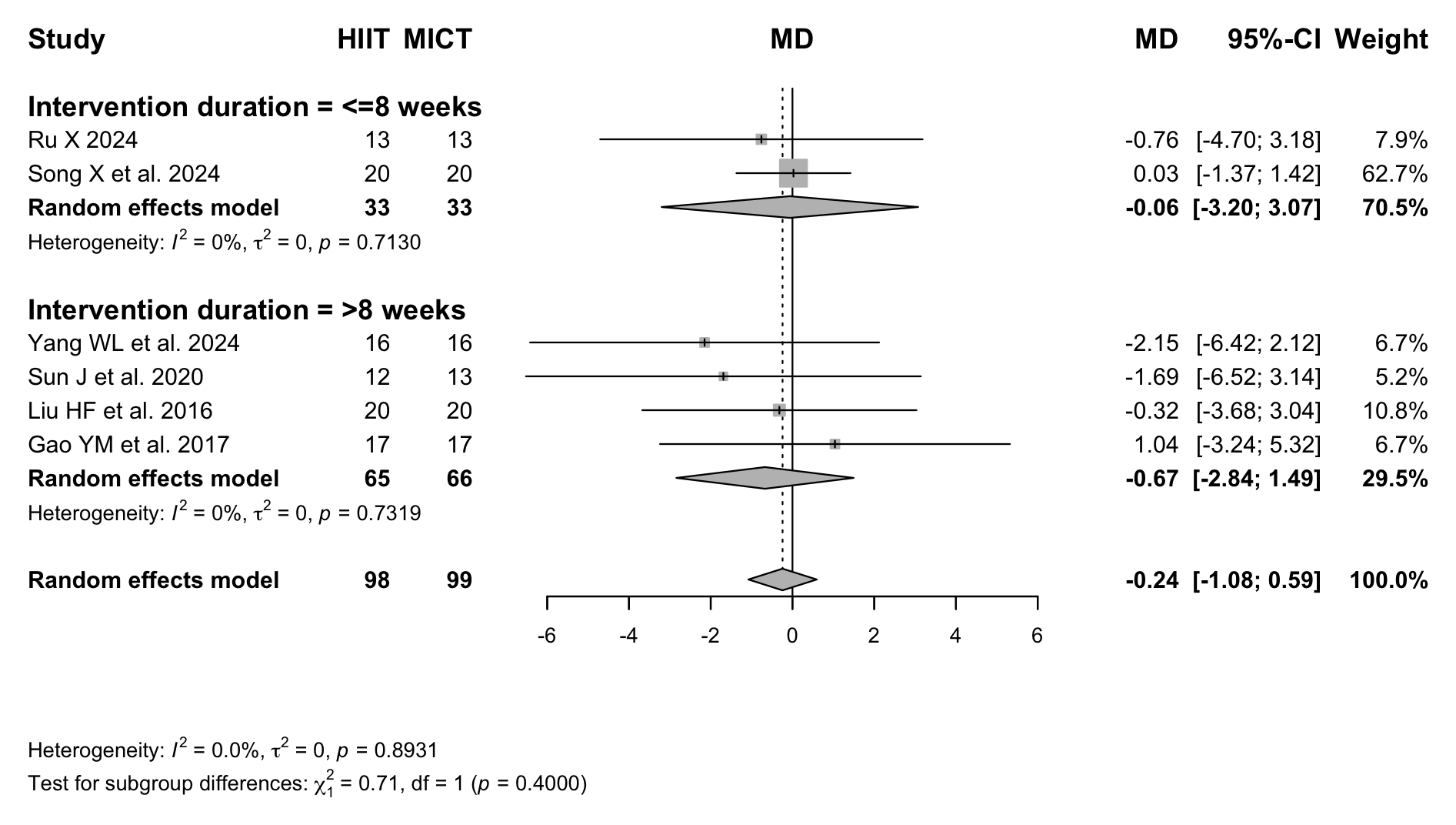


**Supplementary Figure S35. Subgroup analysis of the effect of HIIT on waist-to-hip ratio (WHR) by sex.**


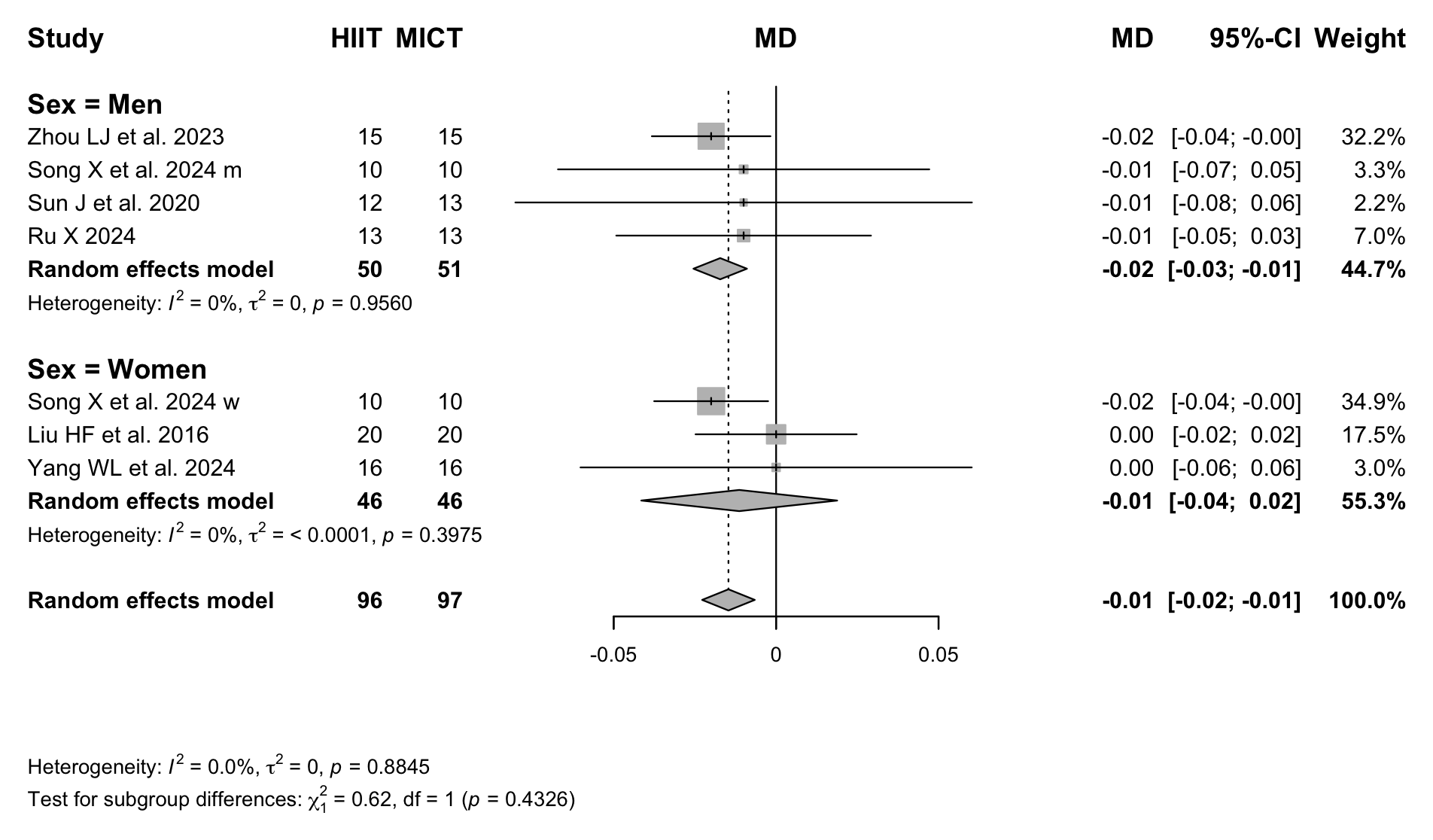


**Supplementary Figure S36. Subgroup analysis of the effect of HIIT on waist-to-hip ratio (WHR) by intervention duration.**


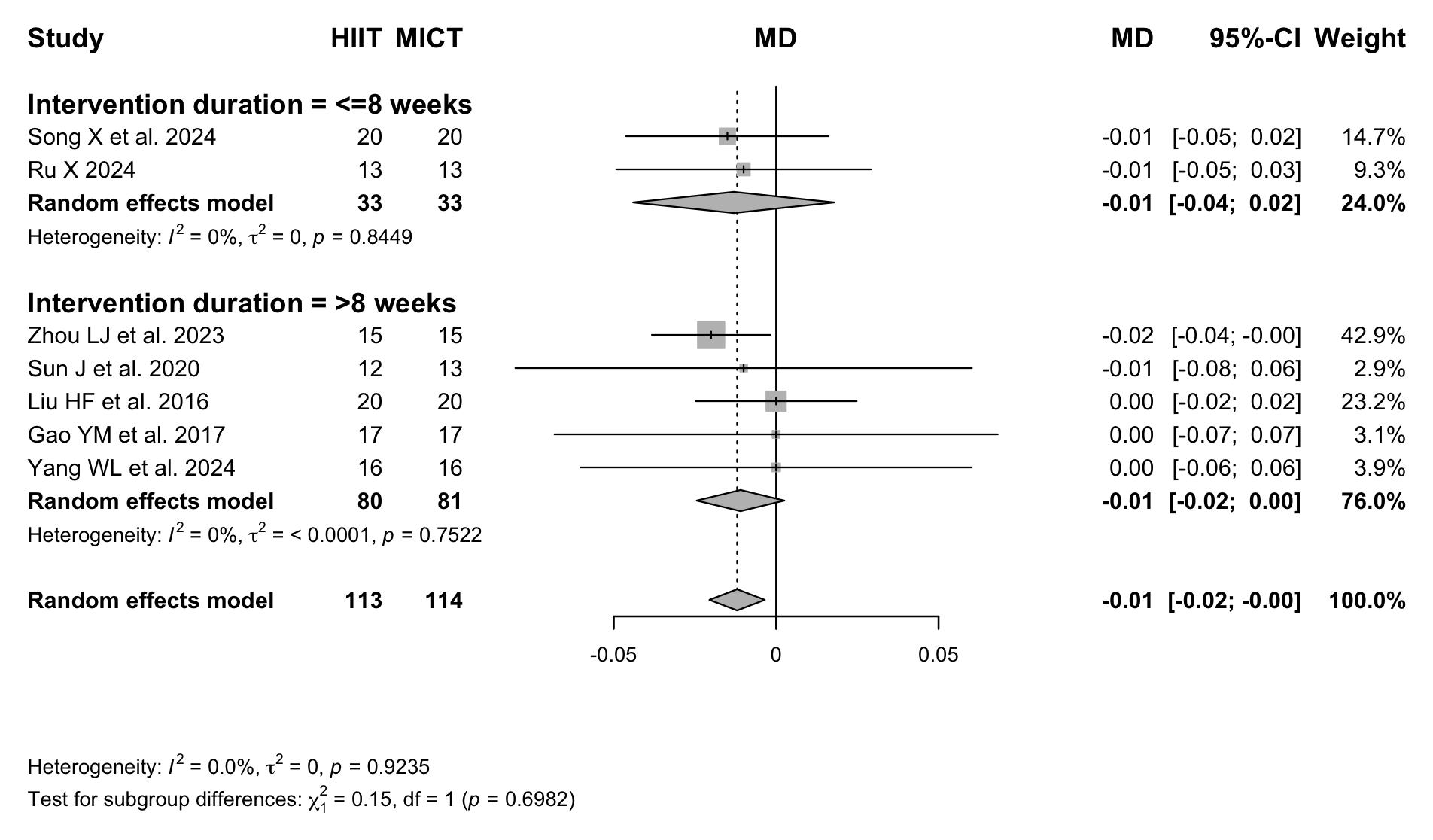


**Subgroup analyses: glycolipid metabolism outcomes (S37-S46)**

**Supplementary Figure S37. Subgroup analysis of the effect of HIIT on fasting blood glucose (FBG) by sex.**


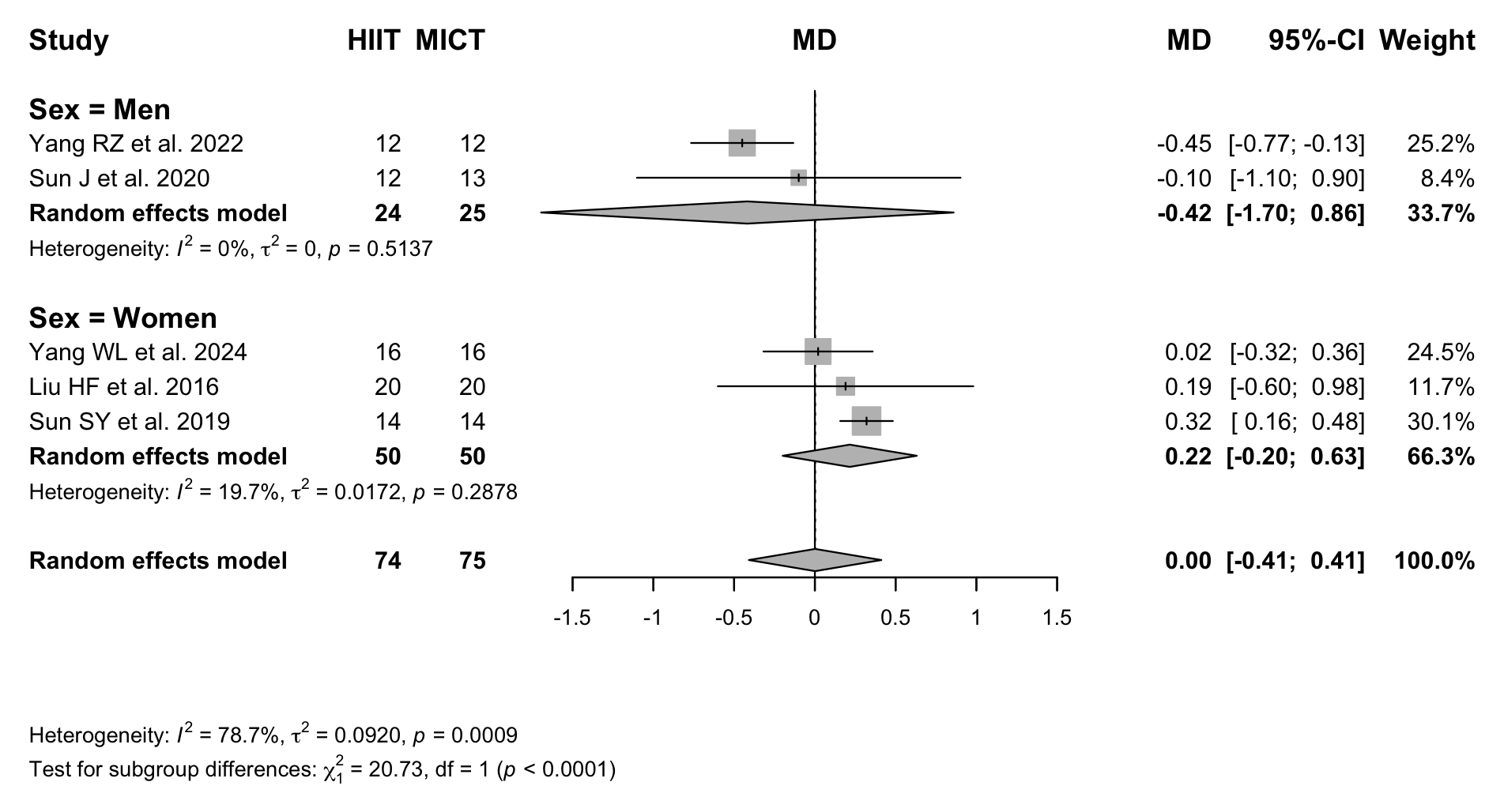


**Supplementary Figure S38. Subgroup analysis of the effect of HIIT on fasting blood glucose (FBG) by weight status.**


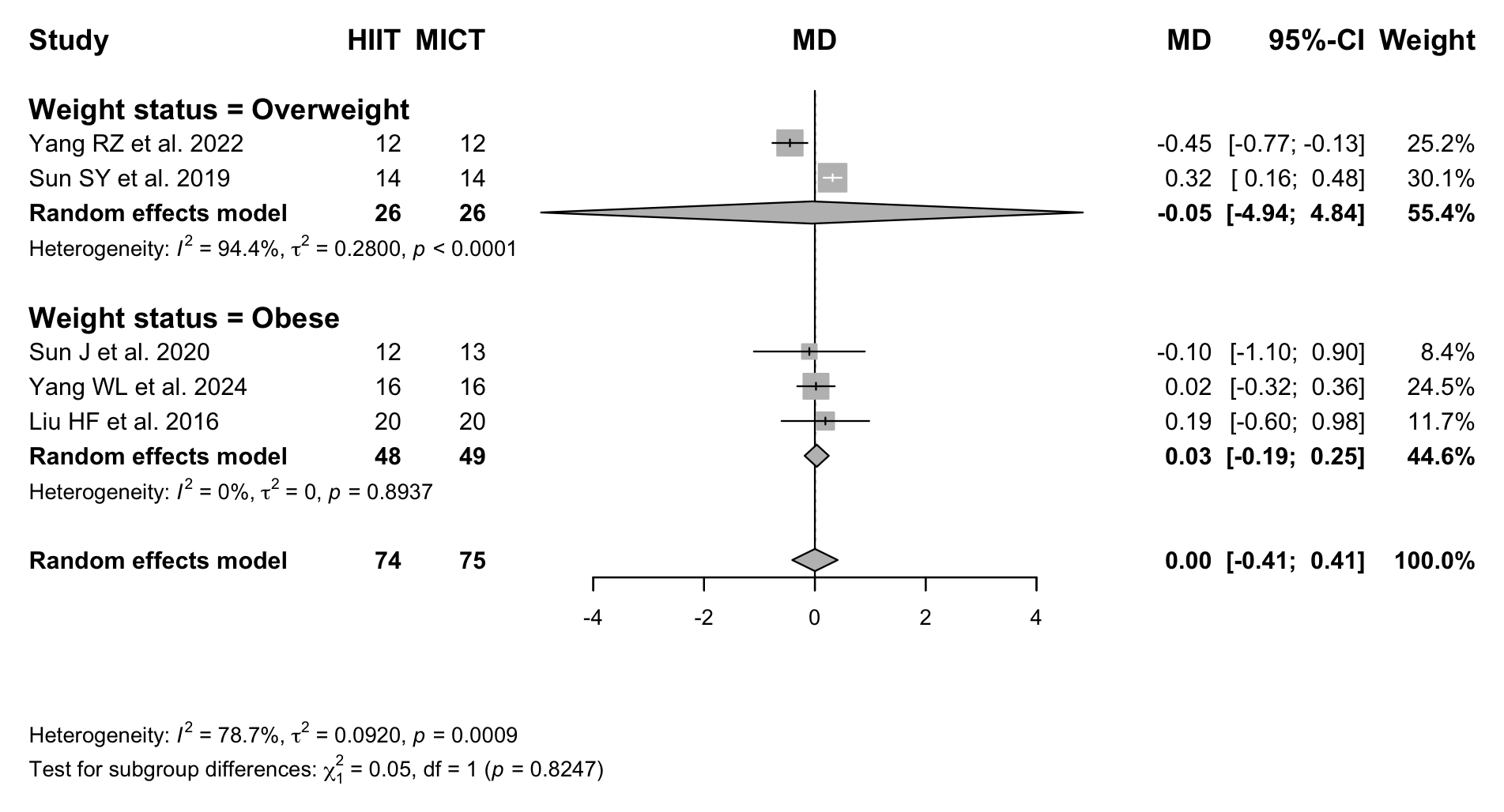


**Supplementary Figure S39. Subgroup analysis of the effect of HIIT on fasting insulin (FINS) by sex.**


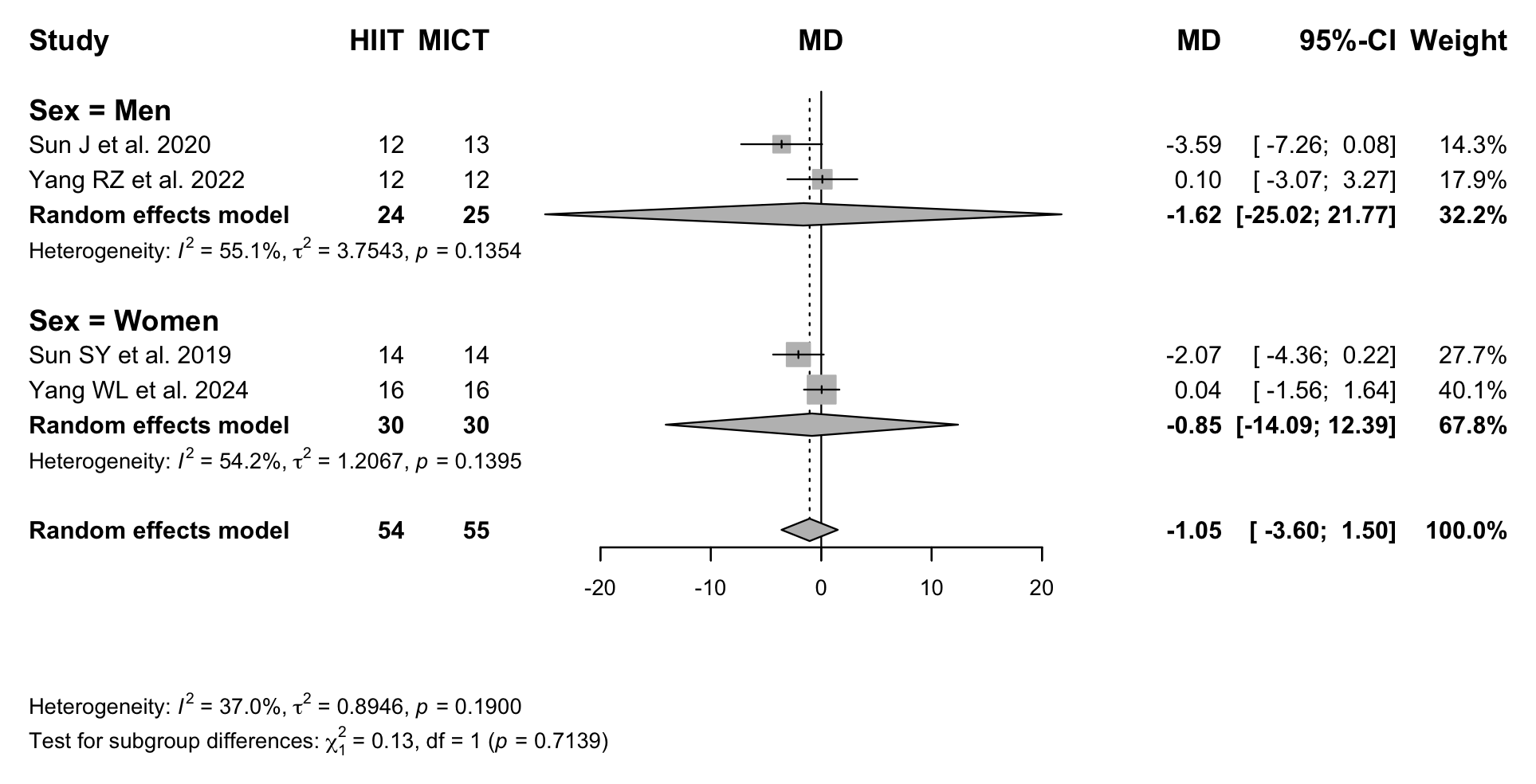


**Supplementary Figure S40. Subgroup analysis of the effect of HIIT on fasting insulin (FINS) by weight status.**


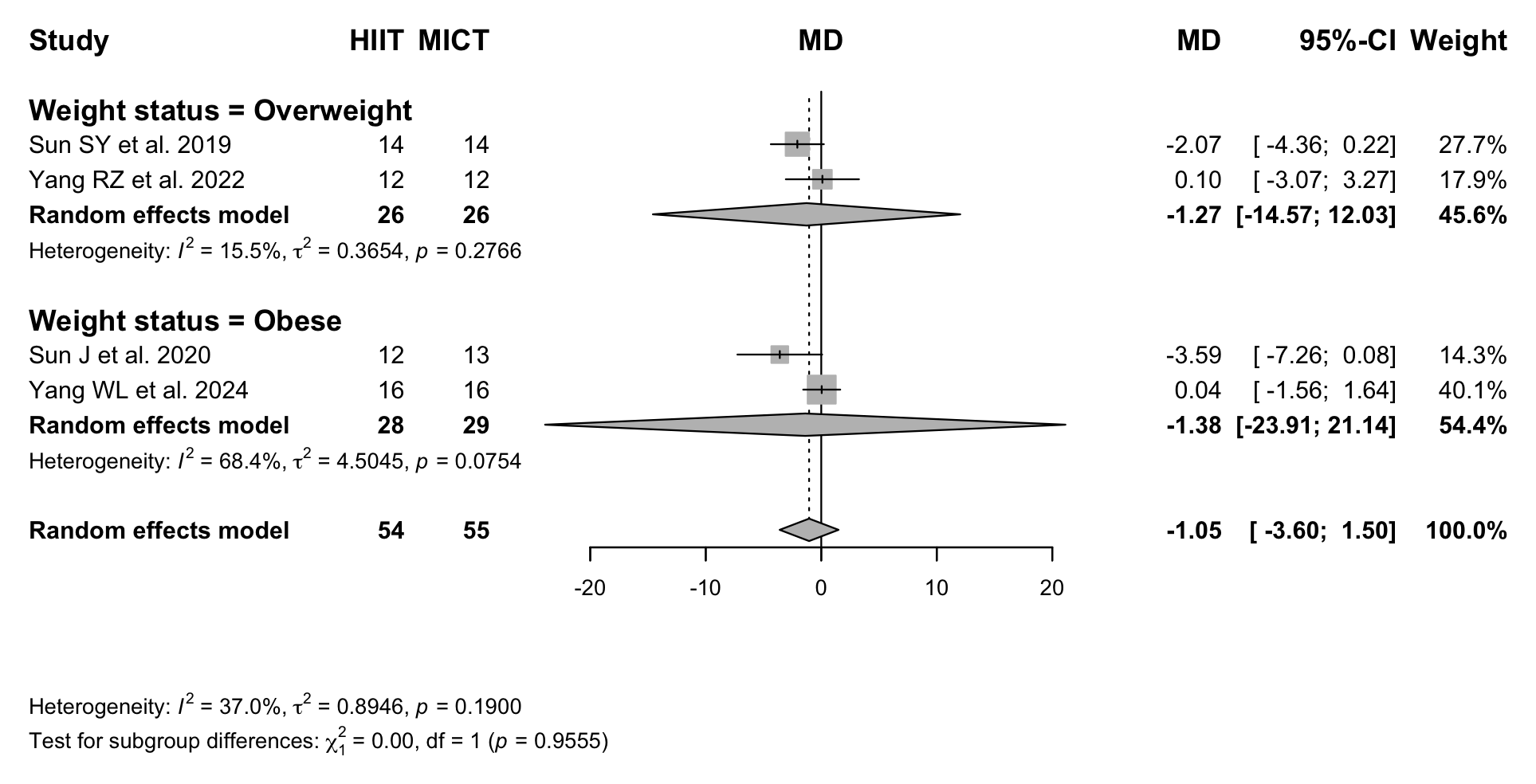


**Supplementary Figure S41. Subgroup analysis of the effect of HIIT on total cholesterol (TC) by sex.**


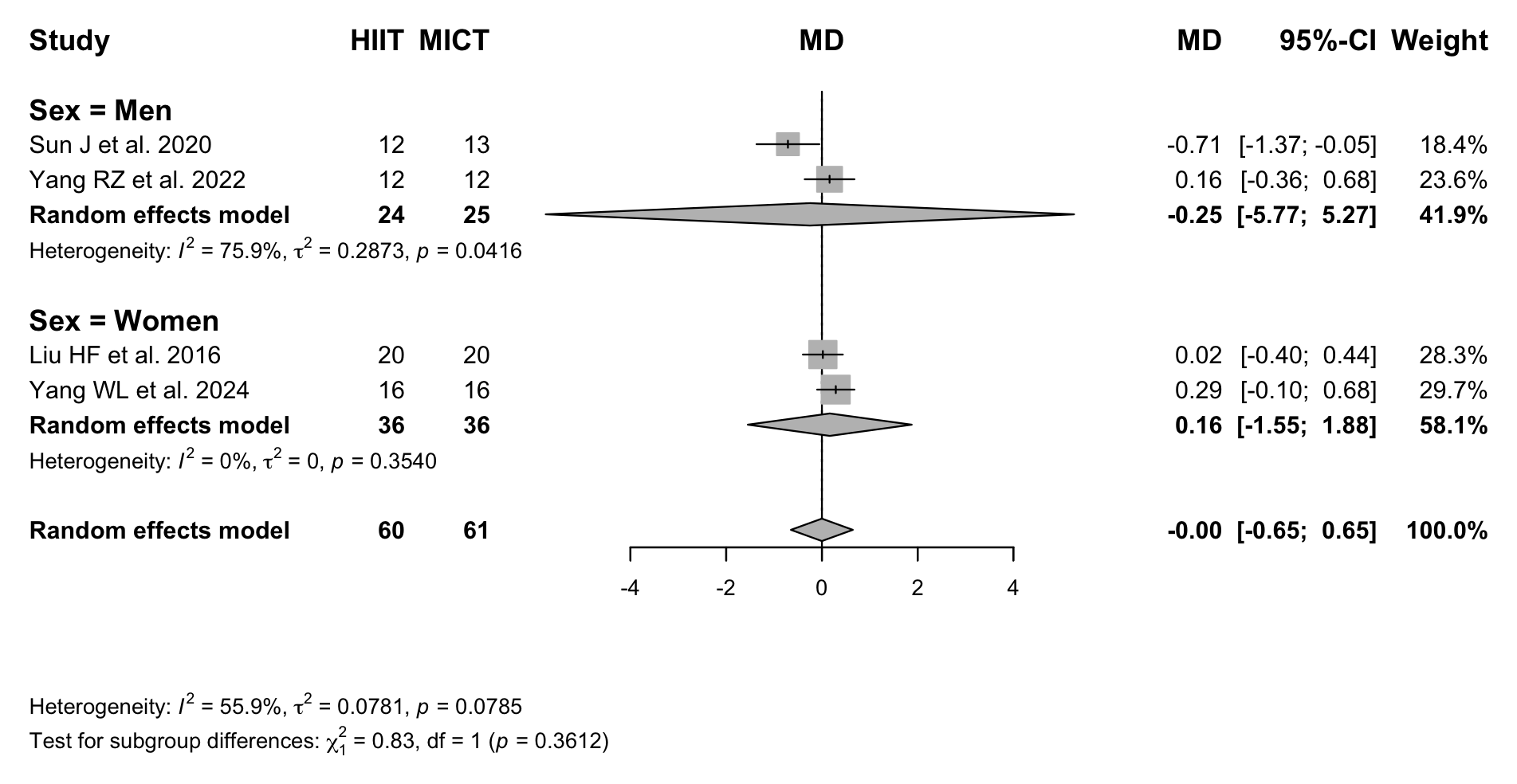


**Supplementary Figure S42. Subgroup analysis of the effect of HIIT on total cholesterol (TC) by weight status.**


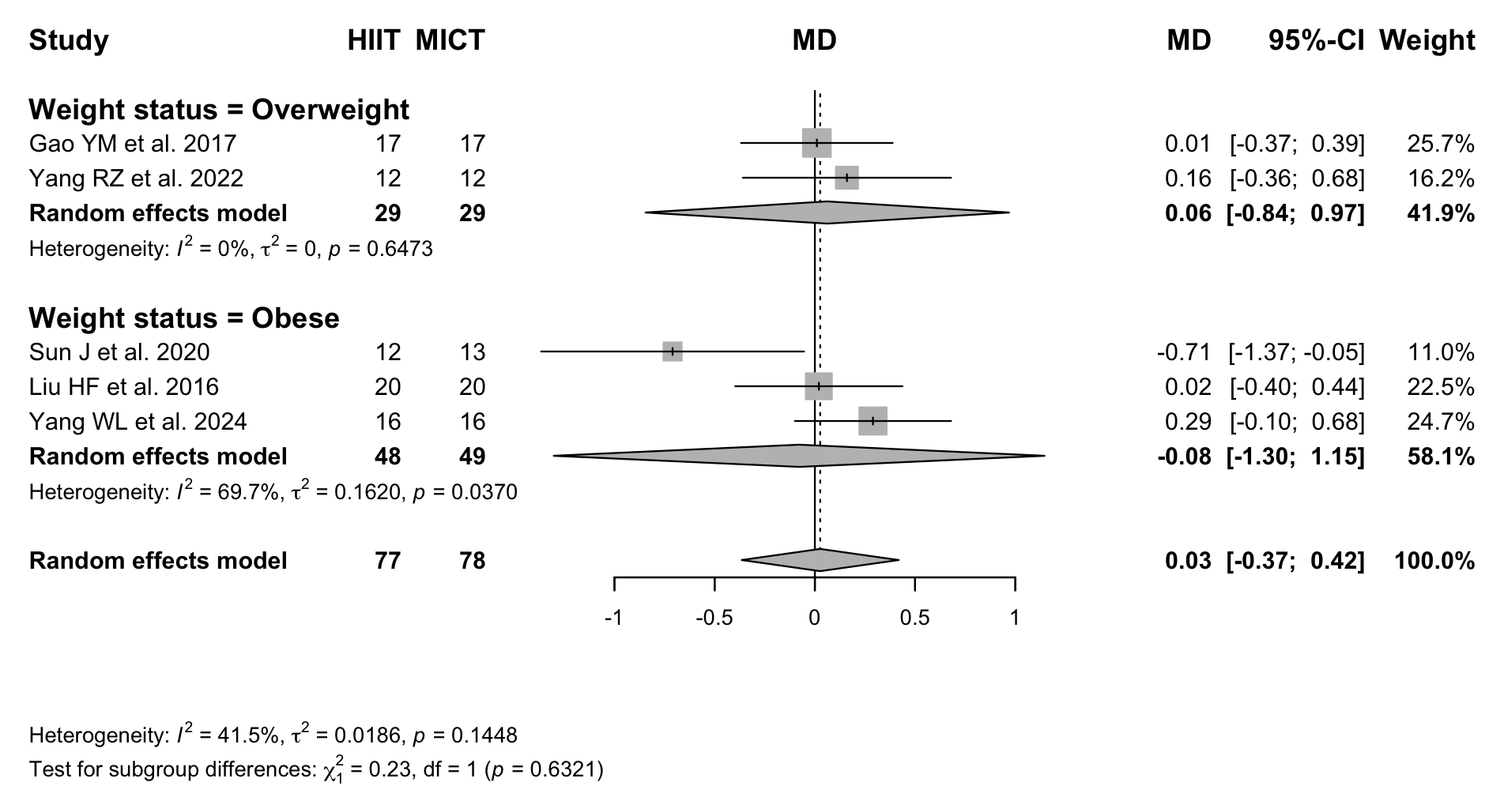


**Supplementary Figure S43. Subgroup analysis of the effect of HIIT on high-density lipoprotein cholesterol (HDL-C) by sex.**


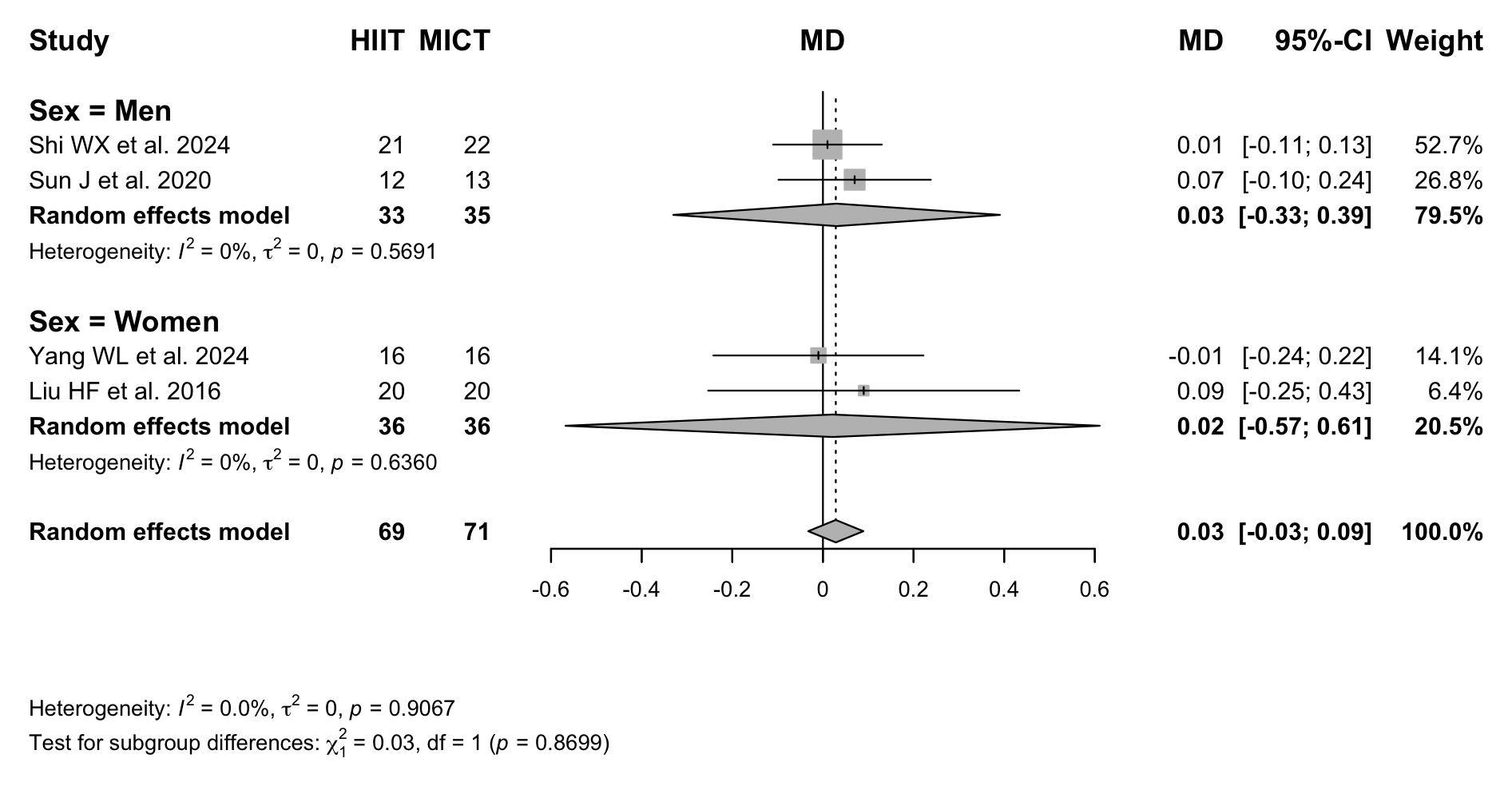


**Supplementary Figure S44. Subgroup analysis of the effect of HIIT on low-density lipoprotein cholesterol (LDL-C) by sex.**


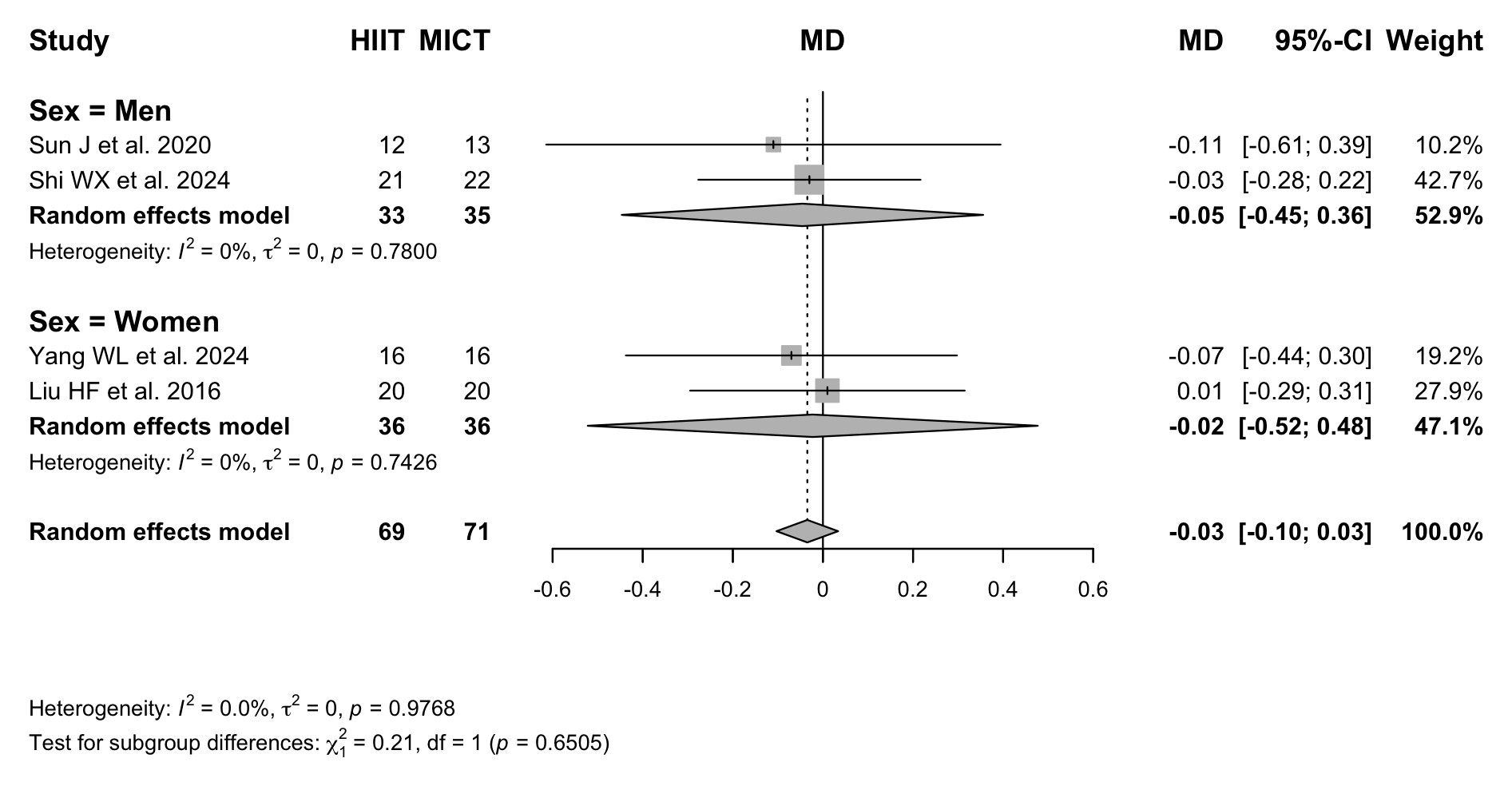


**Supplementary Figure S45. Subgroup analysis of the effect of HIIT on triglycerides (TG) by sex.**


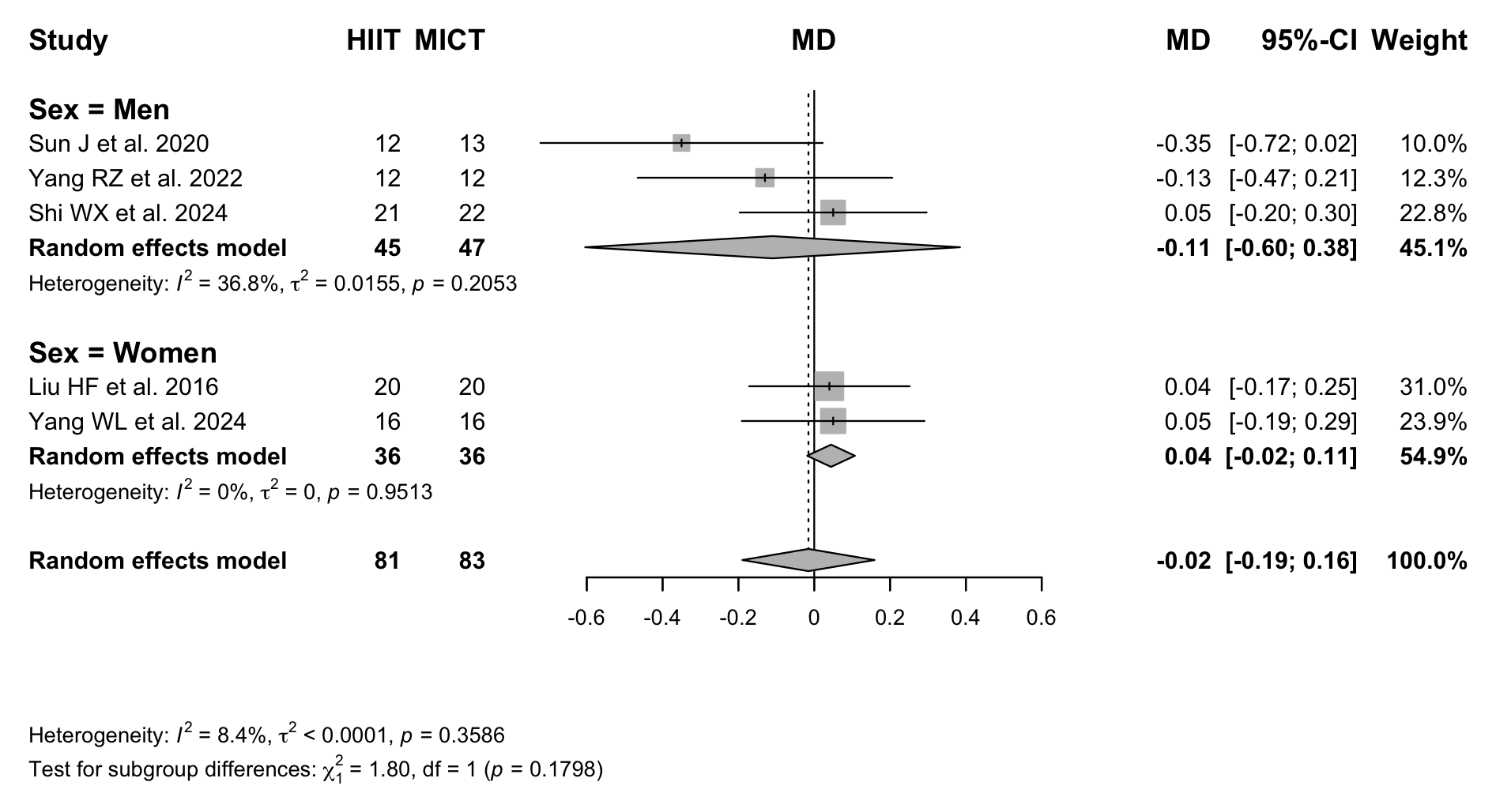


**Supplementary Figure S46. Subgroup analysis of the effect of HIIT on triglycerides (TG) by weight status.**


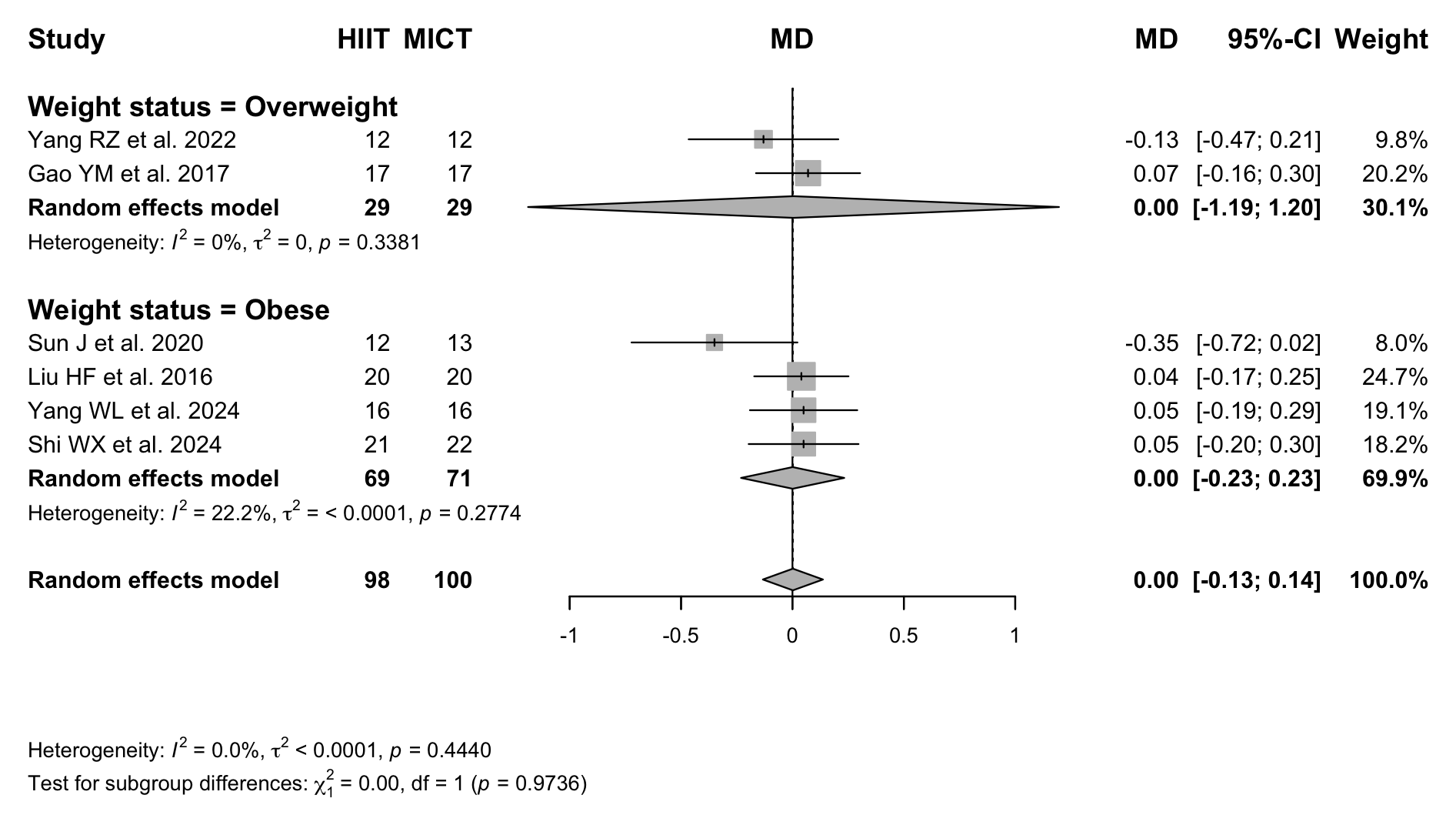


**Leave-one-out sensitivity analyses**

**Supplementary Figure S47. Leave-one-out sensitivity analysis for body mass index (BMI).**


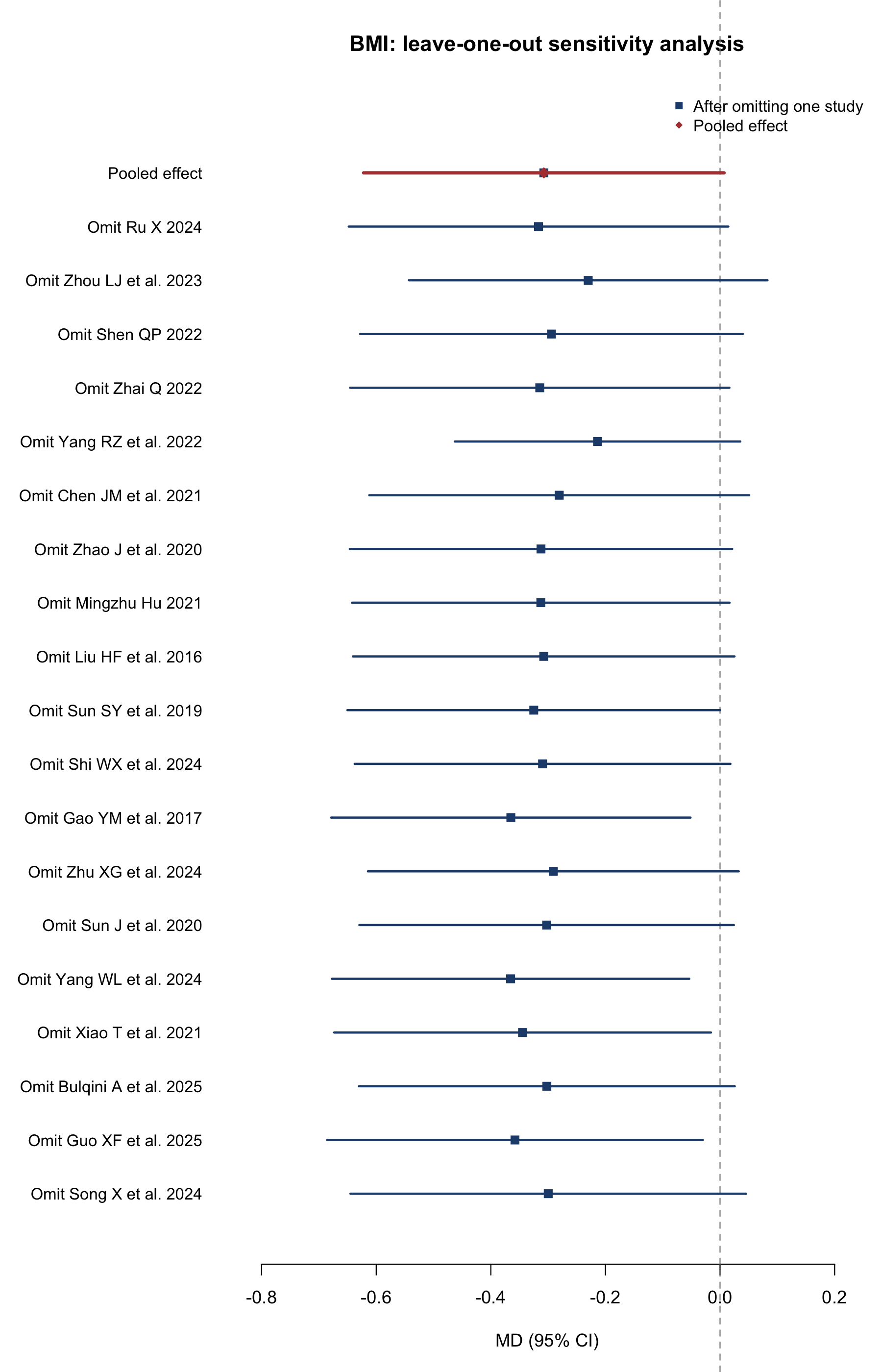


**Supplementary Figure S48. Leave-one-out sensitivity analysis for body fat percentage.**


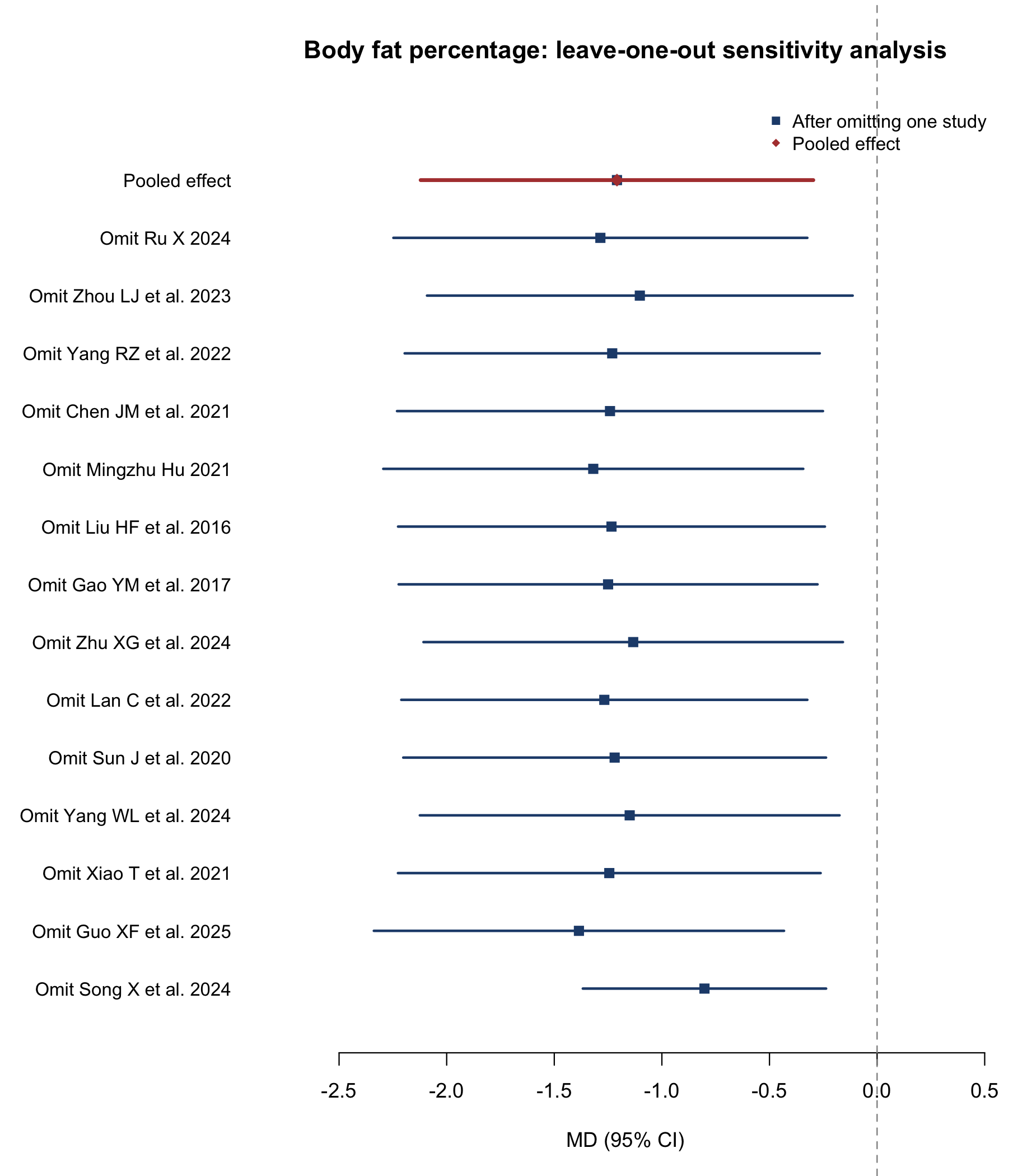


**Supplementary Figure S49. Leave-one-out sensitivity analysis for muscle mass.**


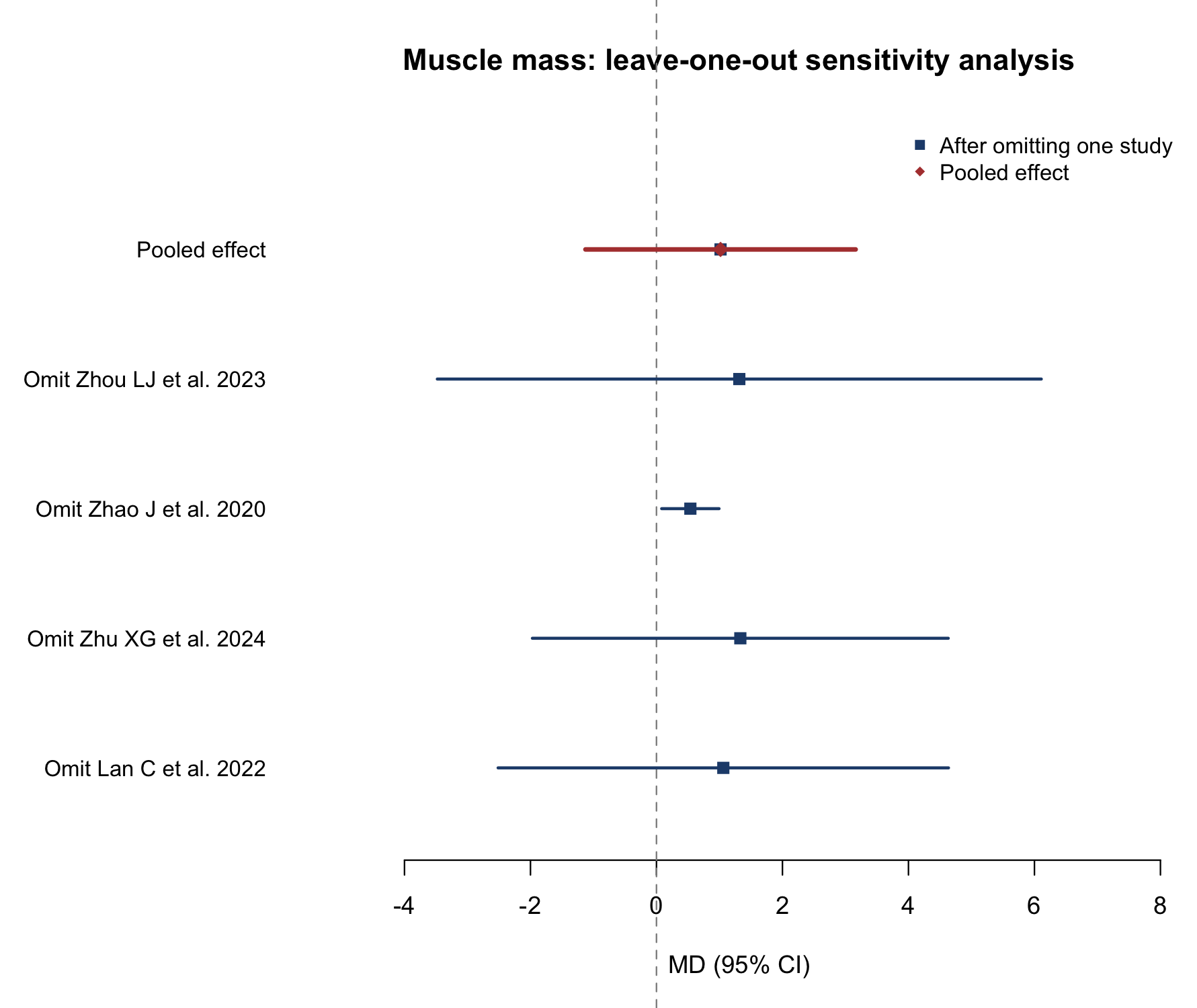


**Supplementary Figure S50. Leave-one-out sensitivity analysis for fasting blood glucose (FBG).**


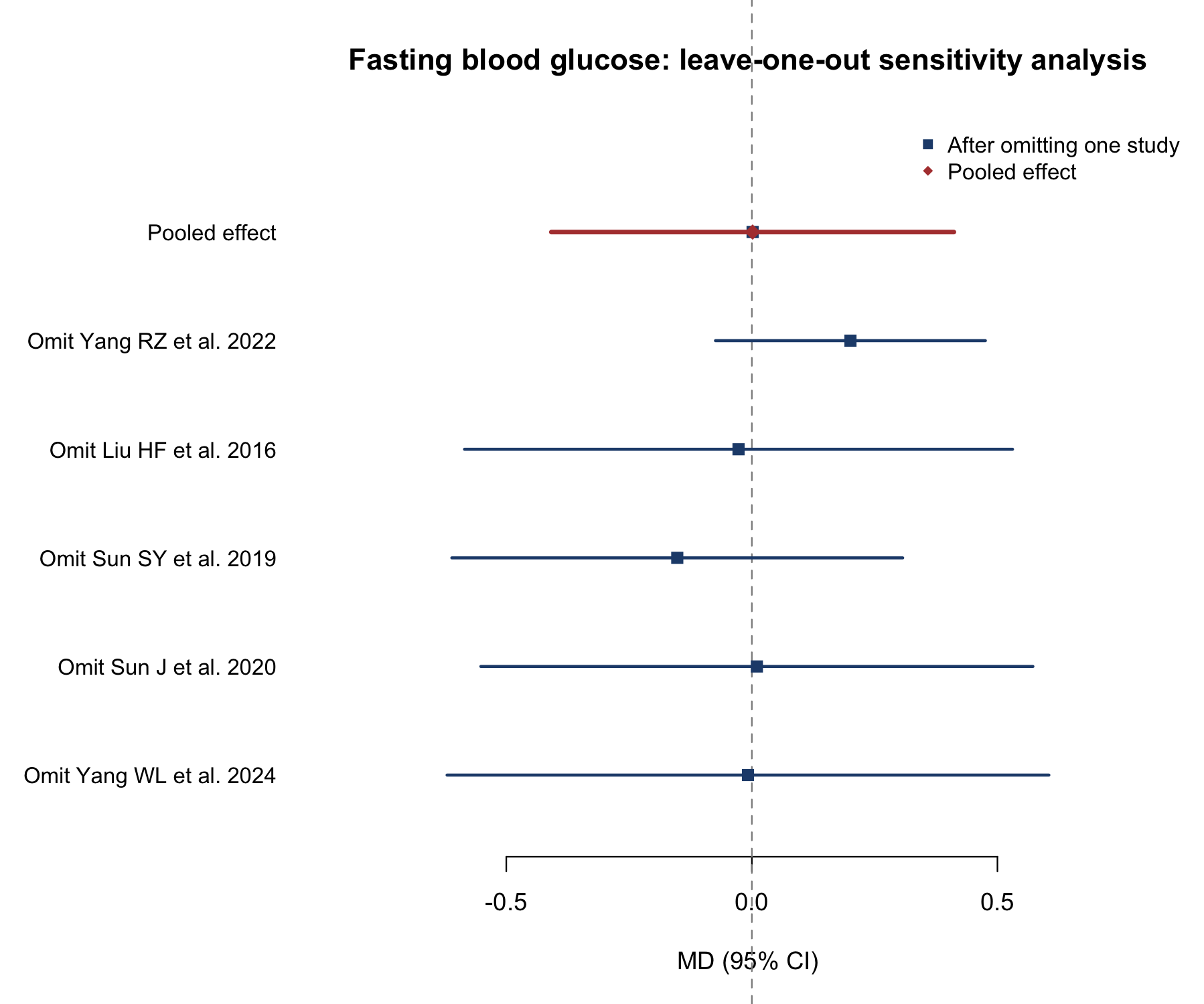


**Supplementary Figure S51. Leave-one-out sensitivity analysis for fasting insulin (FINS).**


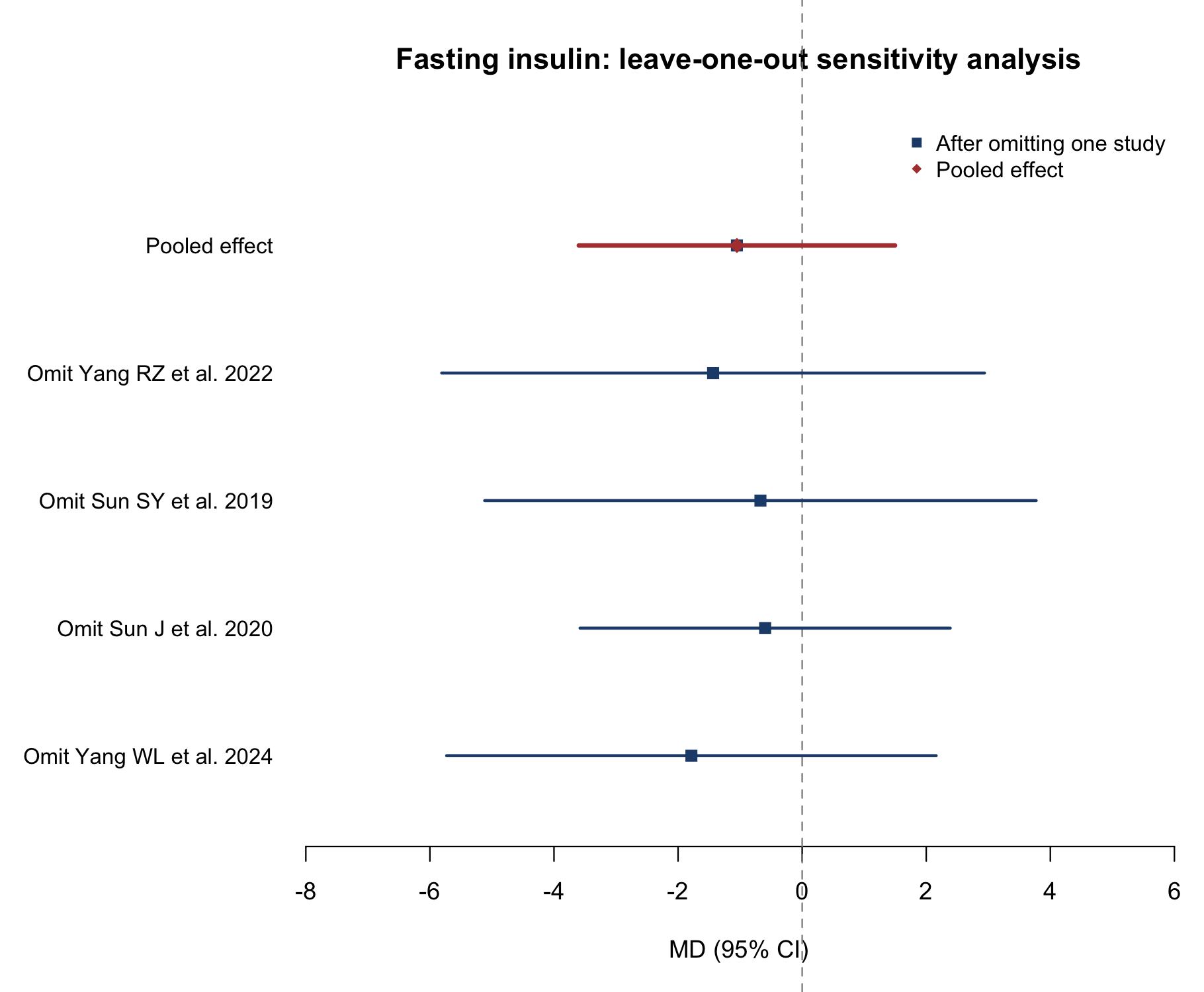


**Supplementary Figure S52. Leave-one-out sensitivity analysis for total cholesterol (TC).**


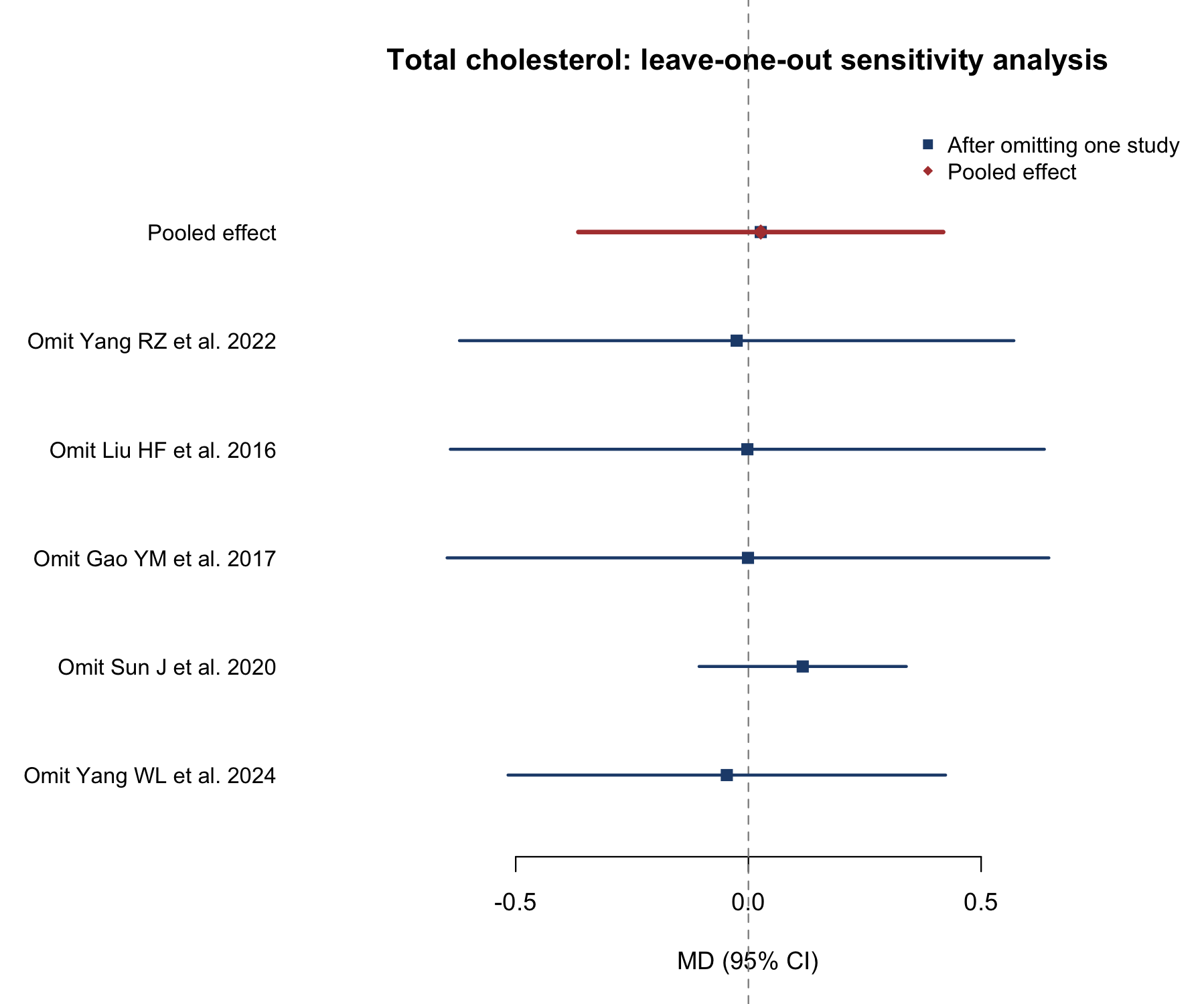


**Funnel plots and publication-bias assessment**

Supplementary Figure S53. Funnel plots with Egger’s regression for body morphology and glycolipid metabolism outcomes.


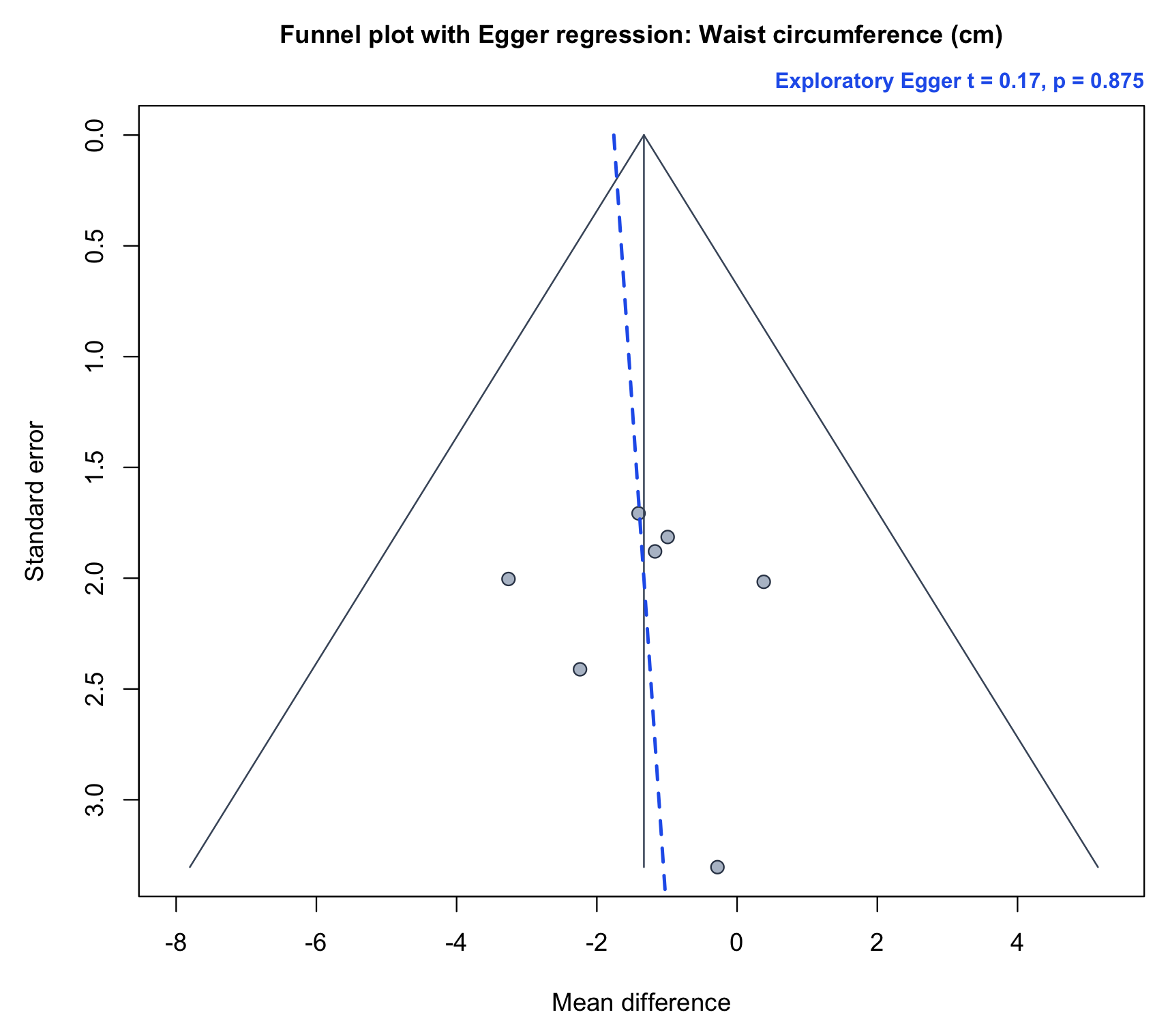

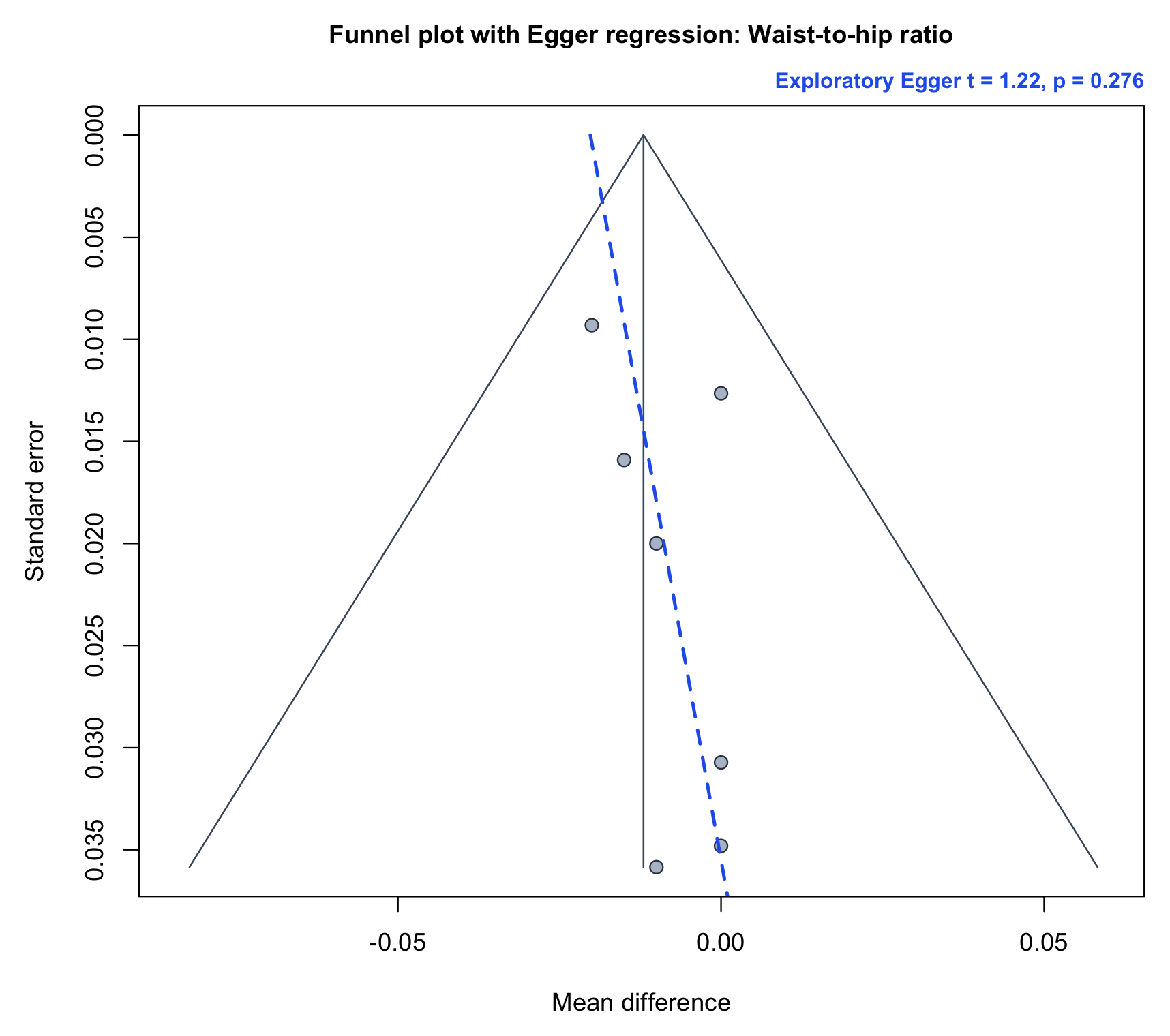


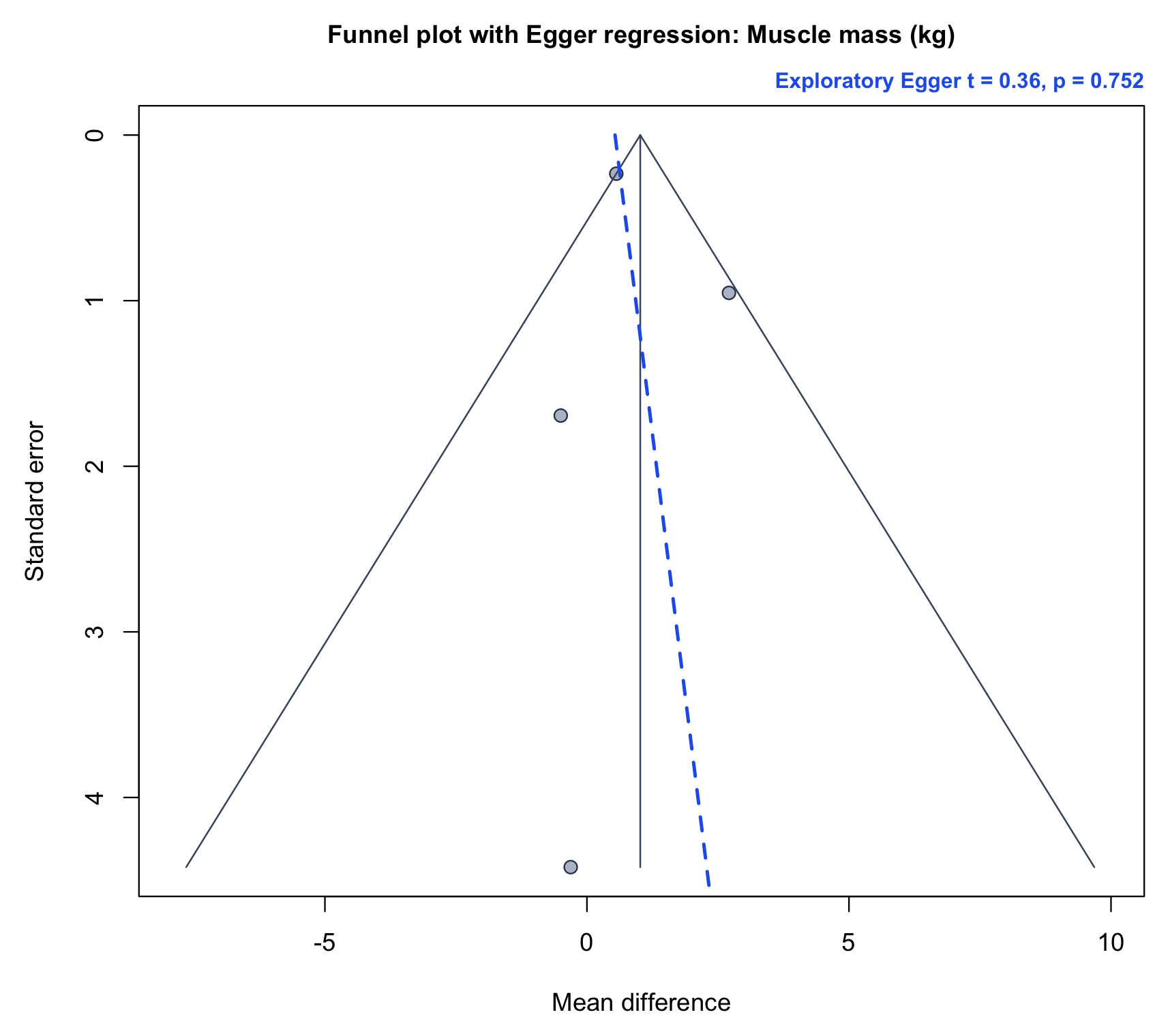

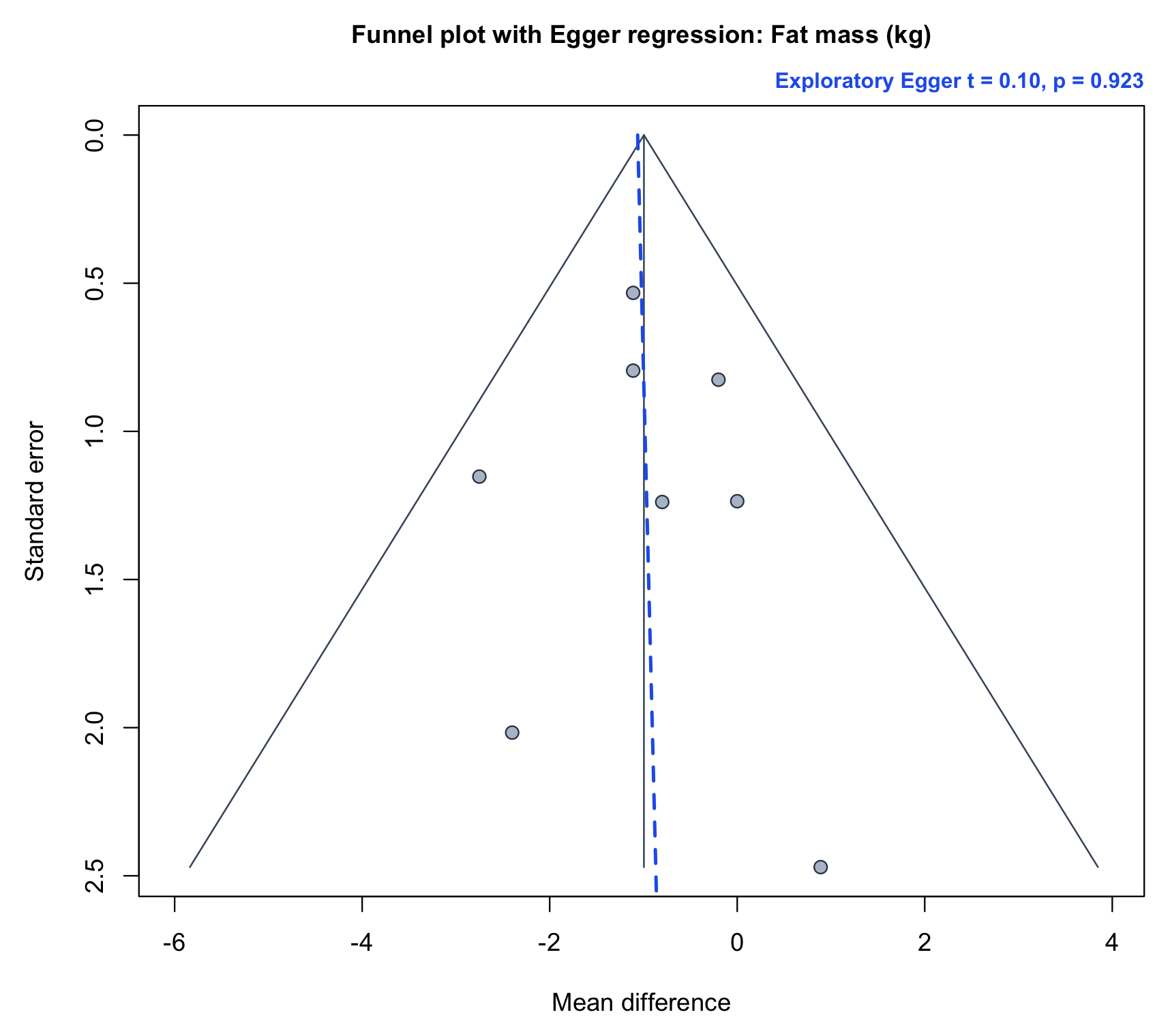

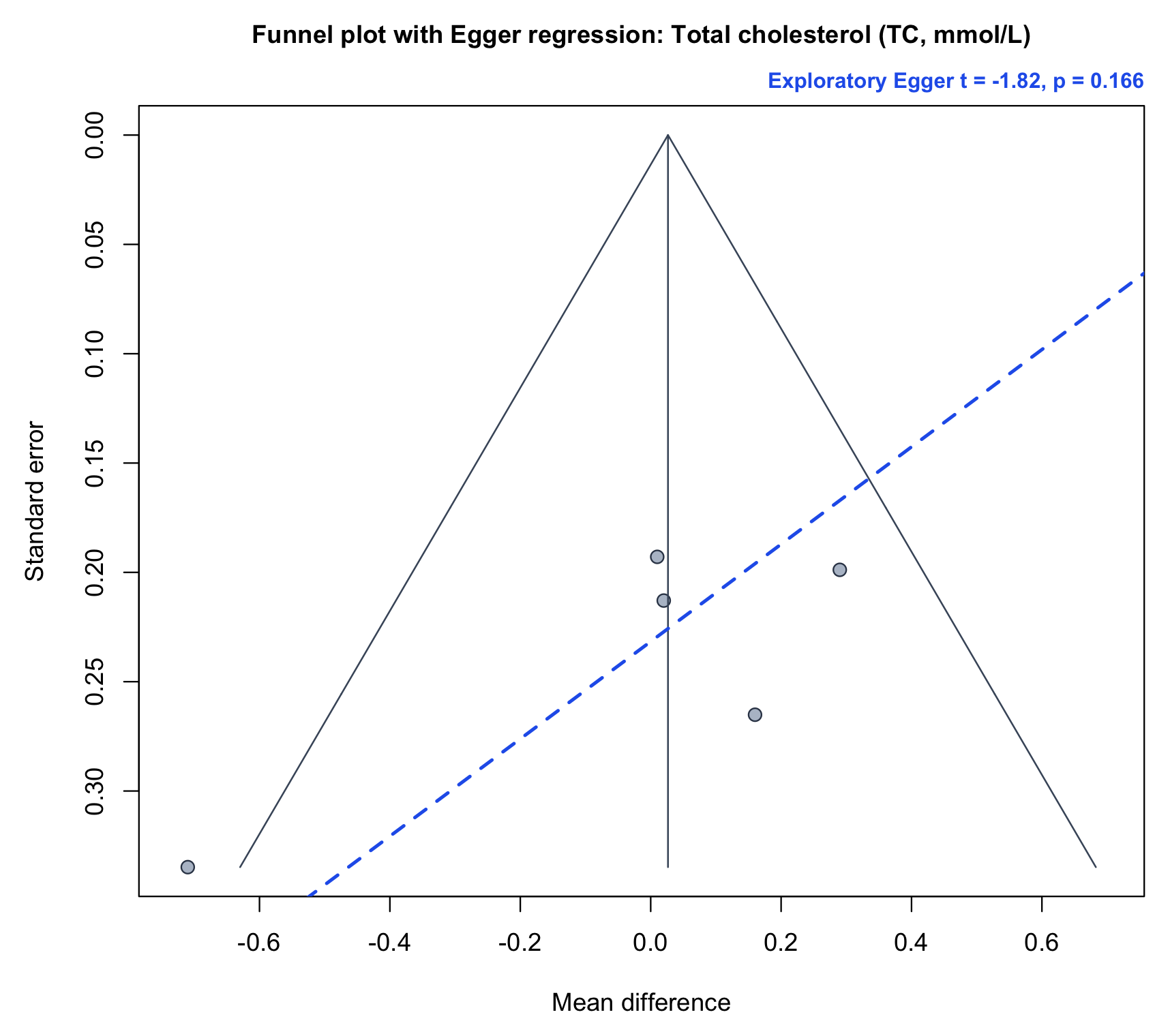

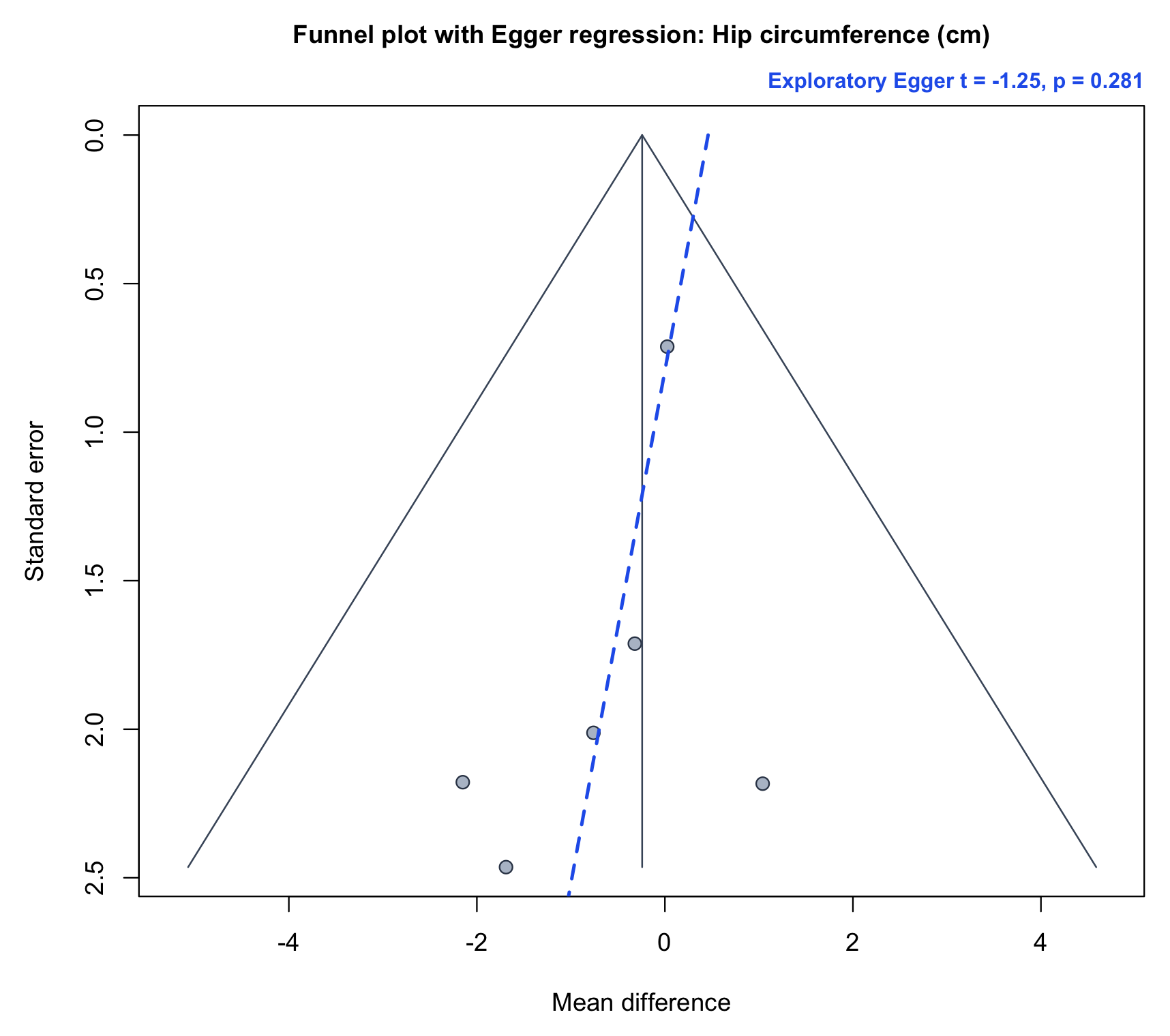


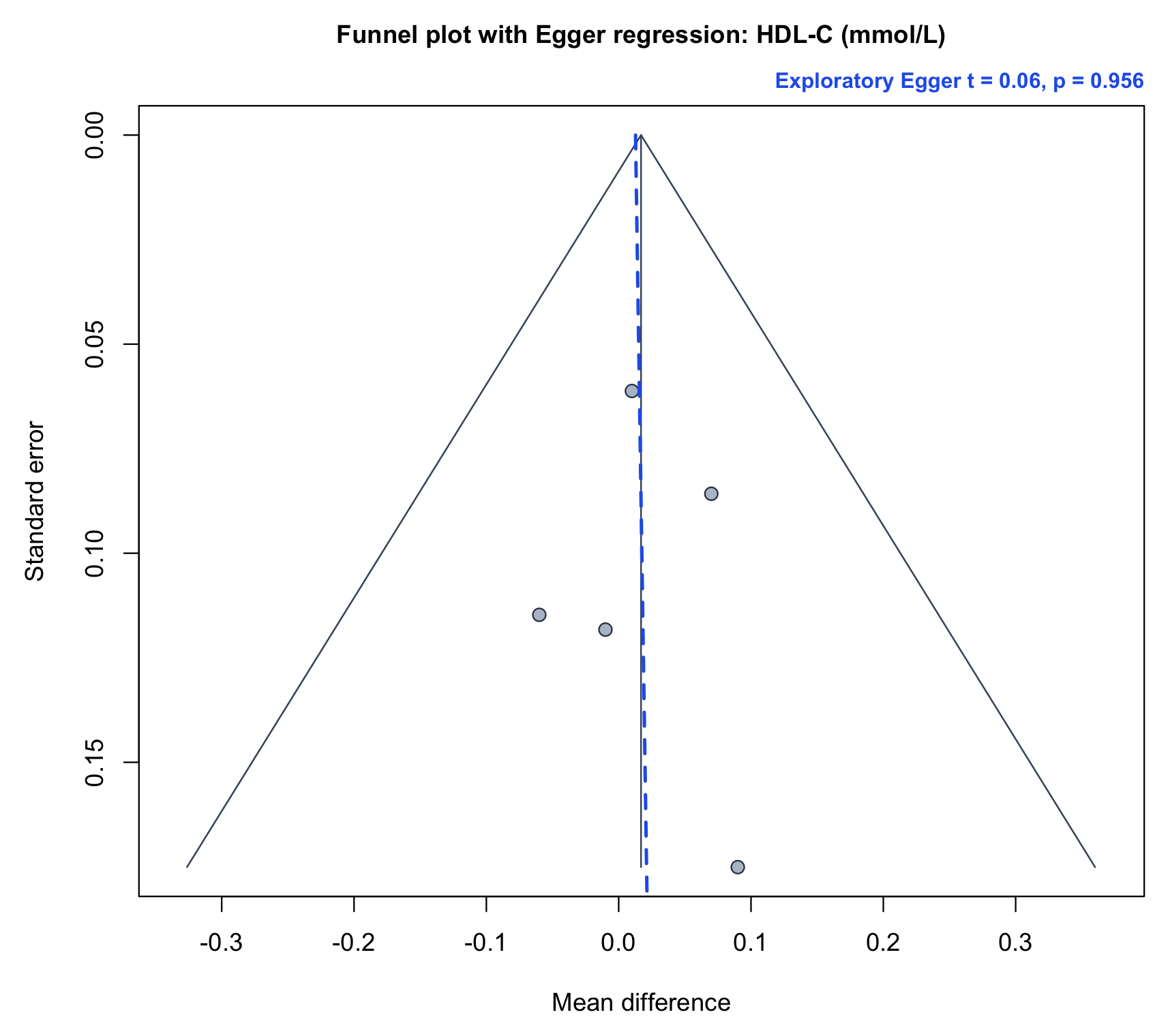

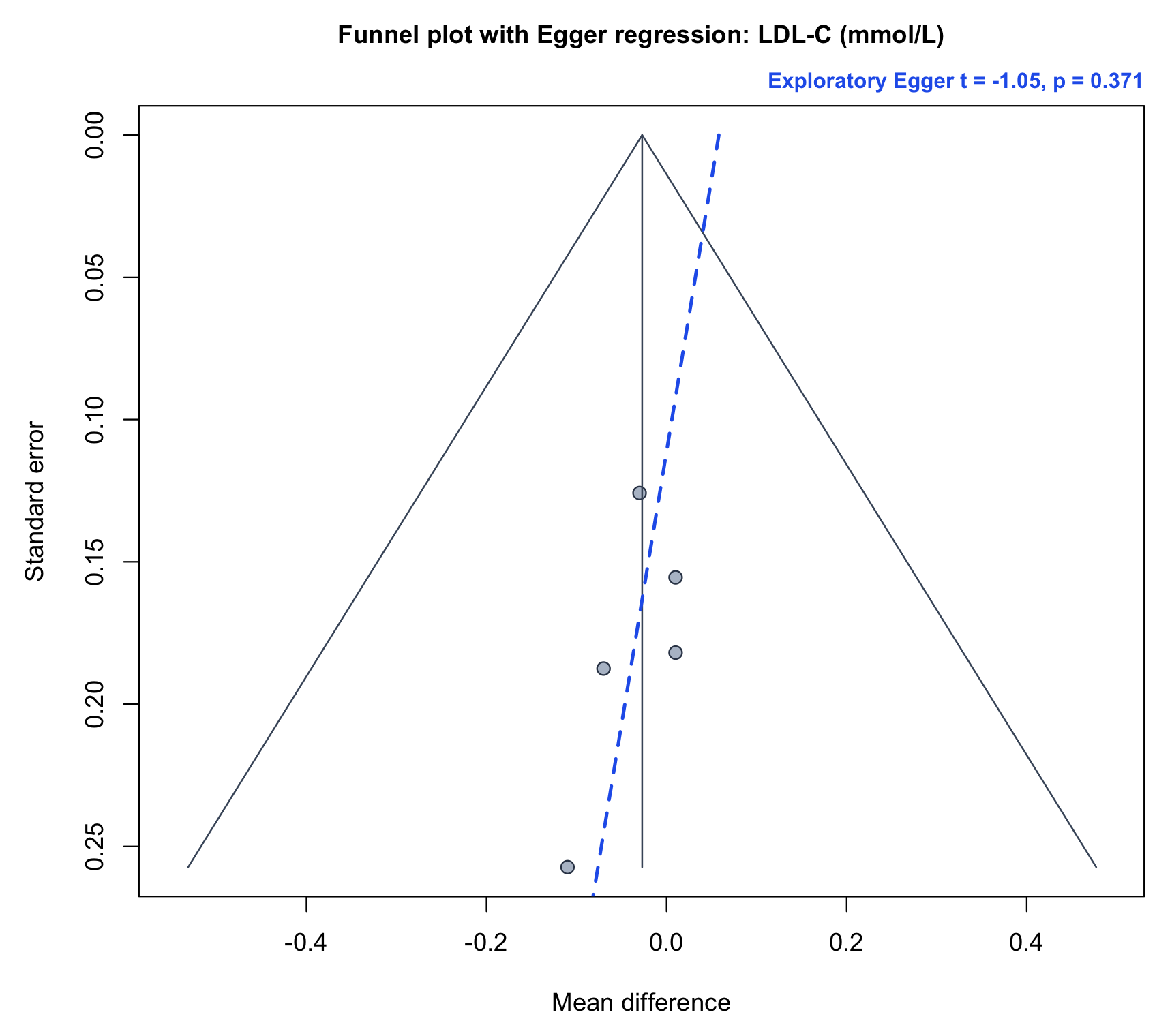


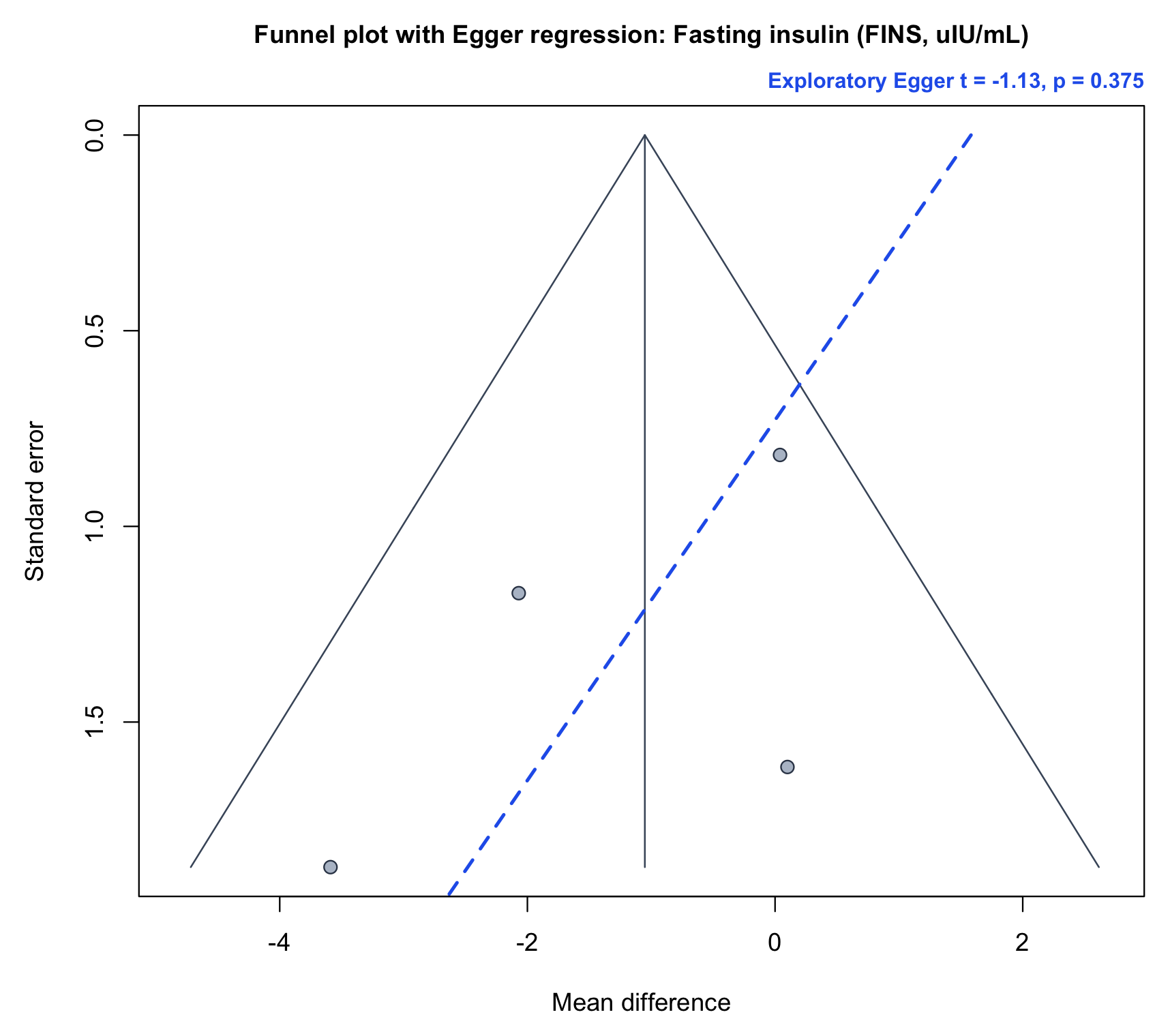


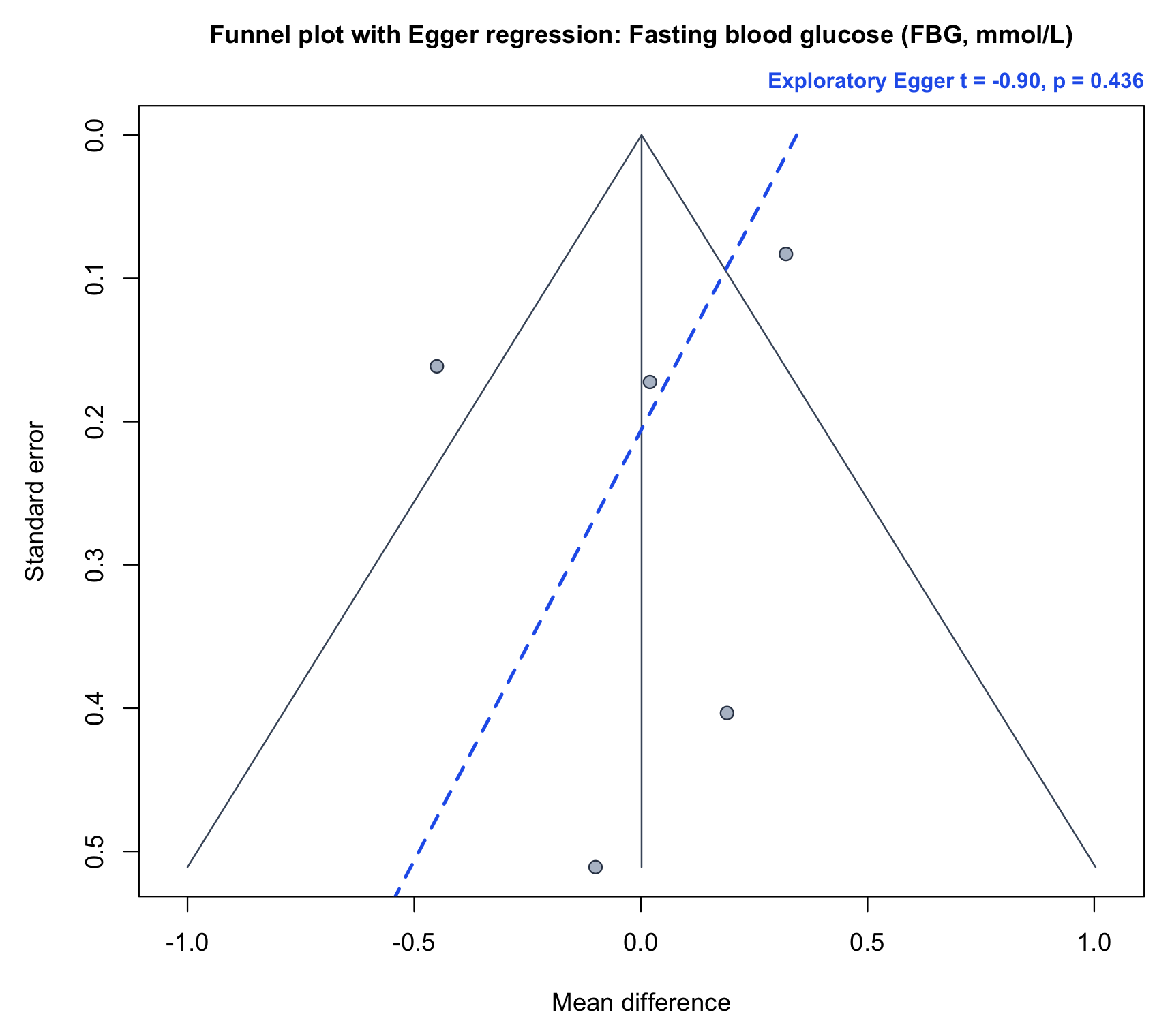


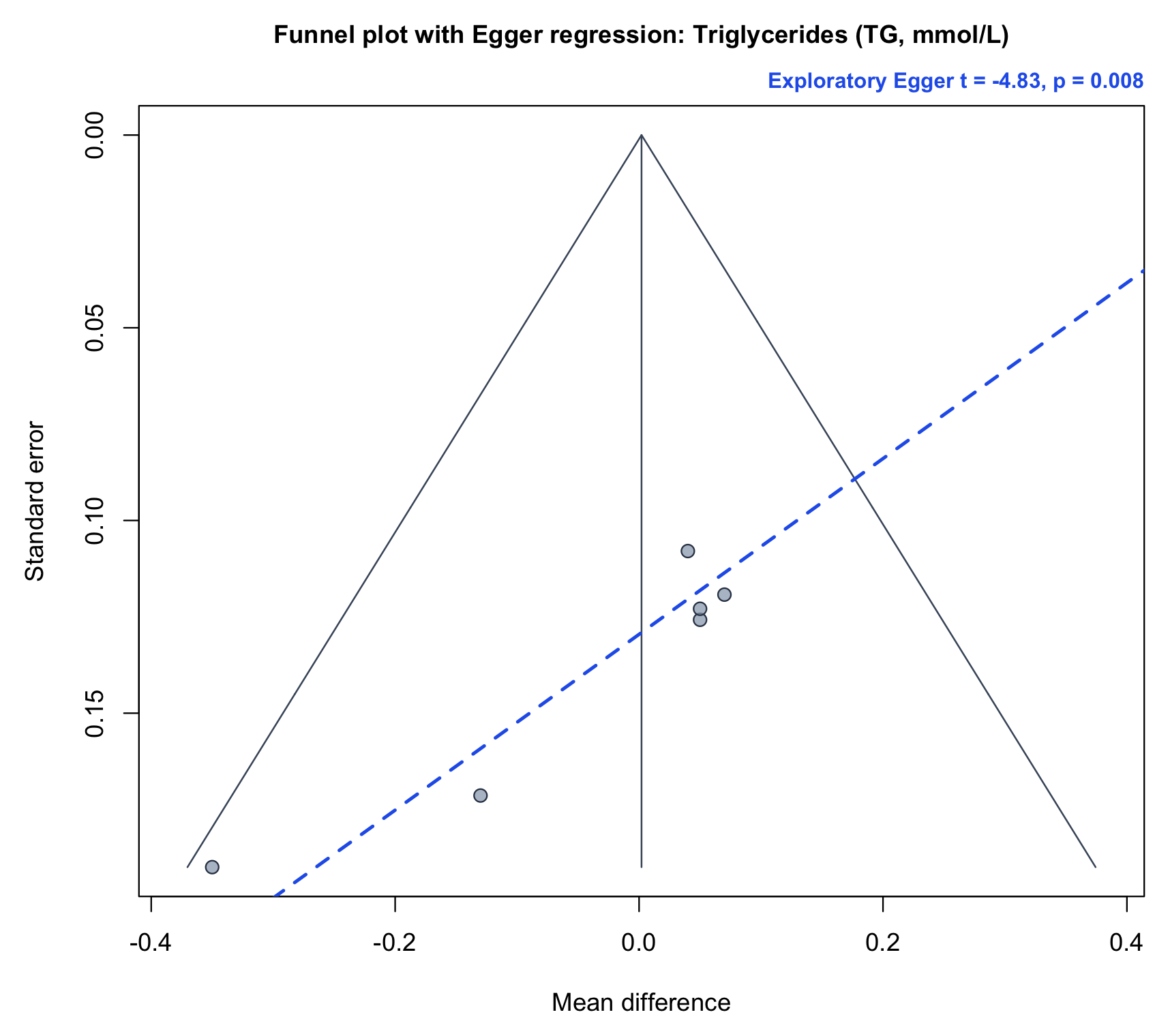

Supplement: Supplementary file 1 [file SupplementaryFile1.docx]
